# Parasite–gut microbiota associations in wild wood mice (*Apodemus sylvaticus*)

**DOI:** 10.3389/fmicb.2024.1440427

**Published:** 2024-11-18

**Authors:** Kirsty J. Marsh, Aura R. Raulo, Joanne P. Webster, Sarah C. L. Knowles

**Affiliations:** ^1^Department of Pathobiology and Population Sciences, The Royal Veterinary College, Hatfield, Hertfordshire, United Kingdom; ^2^Department of Biology, University of Oxford, Oxford, Oxfordshire, United Kingdom

**Keywords:** microbiota, parasite, wildlife, observational, rodent

## Abstract

The mammalian gastrointestinal tract provides a habitat for multiple commensal and pathogenic organisms spanning all three domains of life. Both positive and negative interactions occur between gut inhabitants, with potential consequences for host health. Studies of parasite–microbiota associations in natural systems remain scarce, yet are important for understanding how parasite communities and commensal microbiota shape each other, and how these interactions influence host health. Here, we characterize associations between helminth and coccidial infections and gut microbiota profiles in a wild population of wood mice (*Apodemus sylvaticus*) over 3 years, using two complementary approaches. We first examined parasite–microbiota associations along the length of the gastrointestinal tract through destructive sampling. Then, in a larger non-invasive capture mark-recapture study, we assessed whether gut parasitic infections detected in feces predicted fecal microbiota diversity and composition. We found that while overall microbiota composition was not associated with infection by any common gut parasite species, microbiota richness was associated with gut parasitism in two ways: (i) infection by the trematode *Corrigia vitta* in the small intestine predicted higher microbiota diversity in the caecum; (ii) there was a negative relationship between gut parasite richness and fecal microbiota richness in the non-invasive study. As our results identified associations between gastrointestinal parasites and microbiota alpha diversity, a future experimental study in this tractable wild mammalian system would be valuable to definitively test the directionality of these interactions.

## Introduction

It is becoming increasingly recognized that eukaryotic parasites and bacteria can interact within the host’s gastrointestinal (GI) tract, with potentially important consequences for host health and disease (Reynolds et al., 2015). For instance, parasite–microbiota interactions may help to explain how one or both types of gut inhabitant influence host nutrition (Million et al., 2017; Subramanian et al., 2014), context-dependency in gut symbiont pathogenicity (Leung et al., 2018a; Leung et al., 2018b) and may also help predict non-target effects of drug treatment programs that may impact their effectiveness (Easton et al., 2019). Gut parasites could interact with the microbiota through a wide range of mechanisms including direct physical interaction (e.g., predation; White et al., 2018), altering the physical, chemical, or nutritional environment within the gut, and/or through effects on the host immune system (Leung et al., 2018b; Loke and Harris, 2023). Therefore, parasites and gut bacteria need not co-inhabit in the same region of the GI tract to affect one another but instead may have up- or downstream effects on one another through indirect mechanisms of interaction.

Perhaps the most well-studied mechanisms of parasite–microbiota interactions are those involving the host immune system, as both gut microbes and certain parasites are known to have immunomodulatory abilities (Honda and Littman, 2016; Mcsorley et al., 2013), which together have shaped the evolution of the mammalian immune system (Gause and Maizels, 2016). These complex interactions, involving both innate and adaptive immunity, can occur in both directions, with microbiota effects on immunity affecting a parasite and vice versa. For instance, the intestinal nematode *Heligmosomoides polygyrus* and Lactobacillus bacteria are thought to promote each other’s persistence by inducing tolorogenic T regulatory cell (Treg) responses (Ohnmacht et al., 2015; Reynolds et al., 2014), while *Lactobacillus* are thought to also increase susceptibility to the nematode *Trichuris muris* through modulation of host Th2 responses (Dea-ayuela et al., 2008). Conversely, the gut microbiota is thought to provide some protection against infection by the apicomplexan parasite *Toxoplasma gondii* through priming innate immune responses, as shown in experimentally infected laboratory mice (Benson et al., 2009).

Gut parasites and the microbiota may also interact via physical changes they cause in the gut environment. For instance, laboratory-based experiments have shown that the helminth *T. muris* and protozoa *Eimeria* spp. both induce higher mucus production and alter mucus composition in the gut, which is thought to subsequently promote the growth of mucin-utilizing gut bacteria such as Clostridiales (Collier et al., 2008; Ramanan et al., 2016). Parasite-induced tissue damage can also allow for bacterial translocation across the gut epithelial barrier, with potential impacts on host health (Egan et al., 2012). Parasites and gut bacteria can even engage in direct physical interactions, as, for example, occurs during the hatching of *T. muris* eggs, which require the attachment of commensal bacteria to their polar egg caps (Hayes et al., 2010). Furthermore, *T. muris* ingest bacteria from the gut environment, restructuring cecal microbiota in a way that limits further *T. muris* colonization (White et al., 2018).

Whilst controlled experiments in laboratory animals can enable a better understanding of the mechanisms underlying parasite-microbiota interactions, they do not tell us how parasites and gut microbes may interact in complex natural settings. In nature, both the internal gut and external habitat of the host are highly diverse and dynamic, where hosts are exposed to diverse sets of microbes and parasites, are frequently coinfected, and experience strong selection pressures from multiple sources. As such, many more factors shape gut community variation in the wild compared to laboratory animals. Wild systems therefore provide useful model systems in which to further our fundamental understanding of within-host community ecology, particularly in well-characterized systems where experimentation is possible (Ezenwa and Jolles, 2015; Johnson et al., 2015; Knowles et al., 2013).

Only a handful of studies have, however, examined GI parasite–microbiota associations in wild mammal populations (Scotti et al., 2020) and as such we currently do not have a consistent picture of the implications gut parasite–microbe interactions may have in natural settings. The movement of laboratory mice into semi-natural enclosures, for instance, has been shown to induce parallel changes in host immune responses, gut microbiota composition, and susceptibility to gut nematode infection (Leung et al., 2018a). Another study by Kreisinger et al. (2015) examined helminth–microbiota associations along the length of the GI tract in a population of yellow-necked mice (*Apodemus flavicollis*) and identified a broad range of gut helminth–microbiota associations, the most pronounced occurring between the tapeworm *Hymenolepis* in the small intestine and the Bacteroidales family S24-7 (Muribaculaceae) in the stomach. In the closely related wood mouse (*Apodemus sylvaticus*), analysis of faecal samples suggested nematode infection (largely *H. polygyrus*) was associated with an increased abundance of *Escherichia* and decreased abundance of Lachnospiraceae (Maurice et al., 2015). Finally, in longitudinally sampled mouse lemurs (*Microcebus rufus*), *Eimeria* spp. infection was associated with increased gut microbial diversity, while two species of *Hymenolepis* had varying associations with different bacterial taxa (Aivelo and Norberg, 2018).

Inferring ecological interactions among host-associated symbionts can be done in multiple ways, using either longitudinal or cross-sectional study designs. Longitudinal studies tracking within-host covariance of symbionts over time can be more powerful than cross-sectional studies for inferring real interactions between co-infecting organisms (Fenton et al., 2014). However, this type of longitudinal monitoring requires non-invasive sampling (e.g., from fecal samples), which is not sufficient to detect covariation in parasite and microbiota at different points along the GI tract. Understanding the spatial distribution of parasites and microbes in the gut is important, as it would facilitate further insight into potential mechanisms of interaction. As such, both destructive cross-sectional and non-invasive longitudinal studies have merit in studying parasite-microbiota associations, and the use of both approaches in a single system allows for simultaneous exploration of both parasite–microbiota associations along the GI tract, as well as over time.

Here, we combine both destructive cross-sectional and non-invasive longitudinal sampling methods to examine parasite–microbiota associations in an intensively monitored wild population of wood mice (*Apodemus sylvaticus*). This host species provides an excellent study system for examining parasite–microbiota associations in the wild, as both their parasite and gut microbial communities have been previously well-characterized (Marsh et al., 2022; Maurice et al., 2015; Pedersen and Fenton, 2019; Sweeny et al., 2023). Seasonal dynamics in the gut microbiota of our study population were found to be driven largely by bacterial taxa in the Ruminococcaeae, Muribaculaceae, Lactobacillaceae, and Bifidobacteriaceae families (Marsh et al., 2022).

Furthermore, wild wood mice tend to be co-infected with multiple species of GI parasitic helminths and protozoa, which in some cases (*H. polygyrus*, *T. muris*) have been adopted as laboratory mouse models for human infections. Seasonal dynamics in parasitic infections have been documented for several of these parasite species (Abu-Madi et al., 2000; Higgs and Nowell, 2000; Montgomery and Montgomery, 1988). We, therefore, first examined whether local and/or distant associations between gut parasites and microbiota can be detected along the GI tract (occurring within or across different GI sections), which may hint at potential mechanisms of interaction. We then asked whether gut parasites and microbiota show detectable covariation over time, and in particular, whether changes in parasite infection levels could explain the seasonal microbiota dynamics previously characterized (Marsh et al., 2022).

## Methods

### Fieldwork

#### Non-invasive study

Regular trapping (approximately every 2–4 weeks) was carried out on a single 2.4 ha grid between October 2015 and October 2018 in Wytham Woods, Oxfordshire (51°46’N, 1°20’W), as previously described (Marsh et al., 2022). Briefly, sterilized small Sherman traps baited with six peanuts, a slice of apple, and sterile cotton wool for bedding were set at dusk and collected at dawn the following day. Individuals were PIT-tagged at first capture for identification and the sex, reproductive status, and body mass (g) were recorded for each capture. After tagging and measurements, mice were released at their point of capture to be subsequently followed as part of a longitudinal capture–mark–recapture (CMR) study. From the traps, up to 300 mg of feces was collected for molecular work (including gut microbiota profiling) using sterilized tweezers and stored at −80°C within 10 h of trap collection. Whenever possible, additional feces was collected, weighed, and stored in 10% formalin for later gut parasite screening using fecal flotation (see below).

#### Dissections

Between October 2017 and October 2018 (on the same trapping nights as the non-invasive longitudinal study), trapping was conducted at 8 additional locations each 40–300 m from the main trapping location within Wytham Woods, to perform the dissection study (see Supplementary Figure S1 for a map of grid locations). These grids were sufficiently far from the main grid that no PIT-tagged mice were ever captured there. All trapping grids were located in mixed deciduous woodland with the dominant tree species of oak, ash, and beech. Traps were pre-baited for 2–3 nights to encourage occupancy when set. Any non-target individuals and species were immediately excluded and released at their point-of-capture, as were any juvenile, pregnant, or lactating wood mice. Across 13 trapping occasions throughout the year at these additional grids, a total of 50 wood mice were humanely euthanized by intraperitoneal injection of pentobarbital followed by cervical dislocation to confirm death. Although traps were set on multiple occasions at each dissection grid, for most grids animals were only successfully captured and sampled on one occasion. The same set of morphometric measurements collected in the non-invasive CMR study (see above) were recorded (Marsh et al., 2022), and fecal samples were collected from traps and stored exactly as described above. Euthanized mice were dissected, and the GI tract was divided into five sections that were used for both microbiota and helminth infection characterization: the small intestine was divided into thirds, which approximated the duodenum, jejunum, and ileum, and the caecum and colon constituted the final two sections. For each of these five gut sections, an approx. 1 cm section of tissue and contents was removed from the middle and immediately put on dry ice before storage at −80°C for microbiota characterization. All remaining material for each section was stored in 70% ethanol for later visual examination under a dissecting microscope to quantify helminth burdens. For cestodes, absolute counts (worm burden) were not possible as scolexes (the point of attachment of an individual worm to the host) were typically not found, and therefore only their presence/absence in the whole gut was recorded using the presence of proglottids (which may have come from one or multiple individual tapeworms with unknown attachment site).

### Fecal flotations

Gut parasites were detected from fecal samples using fecal flotation with sodium nitrate solution (Vetlab Supplies Ltd., specific gravity 1.2 ± 0.005). This flotation solution is expected to allow detection of most nematode and cestode species, as well as *Eimeria* spp. oocysts, but not trematode eggs, whose larger eggs would require the use of a flotation solution of a different density. Common pinworm (*Syphacia*) species are also expected to have poor detection using this method due to their mode of reproduction, in that the eggs are directly deposited around the anus and may not be present in feces (Sousa et al., 2016). Formalin-stored fecal samples were sieved and placed in 15 mL falcon tubes before a flotation solution was added to form a meniscus. A microscope cover slip was placed on top and samples spun for 10 min at 1000 rpm in a swinging bucket centrifuge. The coverslip was then removed and placed on a microscope slide for parasite identification. Each coverslip was visually divided into 12 columns for systematic counts of eggs/oocysts under a light microscope at 10x magnification. 40x magnification was used for more accurate species identification where necessary. If the egg/oocyst load was too high for accurate counts across the whole slide, counts were conducted on a smaller section (usually half) of the slide and scaled up to represent the whole slide. Eggs/oocysts per gram (EPG) of feces for each uniquely identified parasite were calculated with the following equation: (count × (12/scale))/sample weight(g).

### 16S rRNA gene sequencing

Genomic DNA was extracted from wood mouse fecal samples and gut tissue samples from the dissection study using Zymo Quick-DNA Fecal/Soil Microbe 96 kits according to the manufacturer’s instructions. Negative controls (water) were included in each extraction batch, with subsequent quality control tests showing no detectable DNA concentrations (using Qubit® high sensitivity assays) or amplification (visualized using gel electrophoresis). The V4 region of the bacterial 16S rRNA gene was amplified with the primers 515F/806R (Caporaso et al., 2011) as detailed previously (Marsh et al., 2022). Since small intestine samples were harder to amplify due to lower concentrations of bacterial DNA (Gu et al., 2013), PCRs for small intestine samples were optimized by eluting extractions in a smaller volume (40 μL rather than 80 μL), the DNA template in the first round PCR reaction was increased to 5.25 μL, primer concentration was increased to 20 μM, and the number of cycles was increased to 25. Library preparations followed a two-step (tailed-tag) approach with dual-indexing (D’Amore et al., 2016) and sequencing was performed on an Illumina® MiSeq with 250 bp paired-end reads. Four MiSeq runs were performed: runs 1 and 2 contained samples from the two 2 years of the non-invasive study, and runs 3 and 4 contained samples from the last year of the non-invasive study plus samples from the dissections. Full details of the microbiota amplification and sequencing methodology can be found in Marsh et al. (2022).

### Bioinformatics

Raw sequence reads derived from samples in both the dissection and non-invasive studies were processed together as part of a larger set of samples (Marsh et al., 2022), using the DADA2 v1.6 pipeline in R to infer amplicon sequence variants (ASVs; Callahan et al., 2016). In brief, reads were trimmed and filtered for quality, and ASVs inferred and putative chimeras were removed before taxonomic assignment using the v128 SILVA reference database. A phyloseq object (McMurdie and Holmes, 2013) was created for further processing of the microbiota data. This included filtering taxa to remove ASVs assigned as mitochondria and chloroplast, and analysis of rarefaction and sample completeness curves in package ‘iNEXT’ to filter out samples with low total read counts (less than 8,000 reads). Putative contaminant ASVs (*n* = 159) belonging to 35 bacterial families (Supplementary Table S1) were identified by their prevalence in five negative extraction controls using the R package ‘decontam’ with default parameters (Davis et al., 2018). For alpha diversity analyses, ASV filtering was limited to the removal of those ASVs we could confidently assign as ‘non-gut’ taxa (mitochondria or chloroplast) or ASVs identified as contaminants by decontam, and abundance filtering was not performed as the removal of rare ASVs has a significant influence on richness estimation (Willis et al., 2017). Frequency ratio-based inference in the R package ‘breakaway’ (Willis and Bunge, 2015) was used to produce non-integer richness estimates per sample. Prior to beta diversity analyses, to guard against potential artefacts or contaminants influencing results, we performed abundance filtering in which very rare ASVs (those with less than 1 read found in less than 1% samples) were also removed from the dataset. ASV read counts were transformed to relative abundance prior to the calculation of Bray–Curtis dissimilarity (McKnight et al., 2019).

### Statistical analyses

All statistical analyses were performed in the R software package (version 4.3.1; R Core Team, 2023). Bayesian linear regression models were performed with the R package ‘brms’ and model fit was assessed by leave-one-out cross-validation with the R package ‘loo’ (Bürkner, 2017; Vehtari et al., 2017). Terms were interpreted as ‘significant’ if the Bayesian 95% credible intervals did not overlap with 0. Estimated richness per sample was used to test for associations between parasite infection and microbiota alpha diversity. Bray–Curtis dissimilarity (variation in presence and abundance of taxa among samples) was used to test for associations between parasite infection and microbiota beta diversity. Correlations among covariates were examined in each dataset prior to modelling (Supplementary Figure S2).

#### Dissection study analyses

To explore the effects of parasite infection on microbiota alpha diversity, we used a series of Bayesian mixed models with estimated microbiota richness as the response variable fitted with Gaussian model family. First, to test whether overall infection status for each helminth (presence/absence across the entire GI tract) predicted gut microbiota richness, we modelled gut section-specific microbiota richness as a function of overall helminth infection status, and infection status*gut section interaction terms. Mouse ID was included as a random factor to control for multiple samples per individual, and both MiSeq sequencing run and read depth (*z*-transformed) were included as technical covariates. To more explicitly test whether helminths might affect the microbiota either up- or downstream of their infection site, we then tested whether microbiota richness was predicted by the presence of each helminth and if this depended on whether helminths were in the same gut section (local) to that from which the richness estimate was derived. To do this, we constructed a series of models, one per gut section, predicting gut-section-specific microbiota richness with the local (in the same gut section) and non-local (summed across all other sections) presence of each helminth species. Covariates included trapping location (/date), MiSeq run and (z-transformed) read depth. Most trapping locations only had data from a single trapping occasion, so trapping location was confounded with trapping date. We therefore included trapping location as a covariate in our models to control for both spatial and temporal variation. Some parasites were never found in a particular gut section (e.g., *H. polygyrus* was never found in the caecum or colon), and in these cases, a local infection term for that parasite was not included when modelling that section’s microbiota richness. For *Hymenolepis* sp., only presence/absence throughout the entire gut was used as a predictor as scolexes were not found and therefore we could not infer this helminth’s exact infection site or burden.

To assess whether helminth infection predicted microbiota composition, we used partial redundancy analysis (RDA, on Hellinger-transformed community data; Legendre and De Cáceres, 2013). To test for overall effects of gut parasite infection status (presence/absence) on microbiota composition, an RDA was performed including all samples with mouse ID as a condition term to control for repeated measures per mouse. The gut section was included as a factor together with interactions term per parasite species with gut section. Read depth and MiSeq run were again included as covariates. To test whether helminth infection status predicted microbiota composition in each gut section and how this depended on whether those helminths were in the same section (local) or a different one to the sampled microbial community, we ran separate RDAs predicting microbiota composition in each gut section, including local (in the same gut section) and non-local (summed across all other sections) presence of each helminth species as predictors. Covariates included trapping location (which is equivalent to trapping day as mice were only caught at each location once), MiSeq run and (*z*-transformed) read depth. We corrected for multiple testing across gut-section-specific models using false discovery rate (FDR). The significance of the overall model and marginal significance of each term was tested by permutations (*n* = 999).

To validate microbiota profiles obtained from fecal samples (as used in the non-invasive study), we used the dissection dataset to compare microbiota alpha and beta diversity estimates from feces with those obtained from different gut sections in the same mice. A Bayesian mixed model was used to assess the differences in microbial richness between fecal samples and gut section samples, with trap location, MiSeq run, and read depth as covariates, and animal ID as a random term (*n* = 6 samples per mouse). Compositional differences in microbial composition between fecal and gut samples of the same mice were modelled with permutational multivariate analyses of variance (PERMANOVA) with the gut section as the main predictor; MiSeq run, trap location, and read depth as covariates; and individual ID as the strata (*n* = 6 samples per mouse). To test whether fecal microbiota profiles were representative of the lower GI tract of an individual, we tested whether the compositional similarity between the colon and feces within an individual was greater than the similarity among the same sample type (colon–colon and feces–feces) across different individuals caught on the same day using permutational Wilcoxon tests with 1,000 permutations on pairwise Bray–Curtis dissimilarities. To directly test how similar beta diversity patterns among individuals were when using either fecal samples or colon samples, we performed a Procrustes analysis on Bray–Curtis dissimilarity.

#### Non-invasive study analyses

To test whether binary infection status for each parasite predicted fecal microbial alpha diversity (estimated richness), we used a Bayesian linear mixed model with Gaussian model family structure. We then constructed a model to test whether the overall richness of parasites detected in feces predicted fecal microbiota richness (multiple infections with 2 or more parasites were grouped). Both models included collection date, MiSeq run, and read depth as covariates and individual ID as a random factor to control for repeated measures for some mice. A redundancy analyses (RDA) on Hellinger-transformed data (similar to that used to analyze the dissection dataset) was used to test whether gut parasite burdens detected in feces predicted fecal microbiota composition. Parasite burdens were fitted as log-transformed variables (log(1 + egg/oocyst count)), mouse ID was included as a conditional term, and read depth and MiSeq run were included as covariates. Significance was tested by permutations (*n* = 999). A Mantel test was used to test whether fecal parasite community dissimilarity (variation in parasite presence and abundance among fecal samples as detected via flotation) correlated with fecal microbiota dissimilarity (variation in microbiota presence and abundance among fecal samples as detected via 16S sequencing), using Bray–Curtis dissimilarities for both parasites and microbes and including only samples in which at least one parasite was detected (*n* = 142 samples, 999 permutations).

To examine whether previously documented seasonal shifts in the gut microbiota (Marsh et al., 2022) might be influenced by gut parasitic infection status, we used a generalized additive mixed model (GAMM, R package ‘mgcv’) to model values from the first axis of a principle coordinate analysis (PCoA) performed on Bray–Curtis dissimilarity (PC1 values) as a function of both season (day of the year fitted as a cyclic cubic spline term) and parasite burden variables (*z*-scores of eggs/oocysts per gram). PC1 values were taken from an ordination performed on a larger longitudinal set of 448 fecal samples (of which the 223 samples analyzed here are a subset, Supplementary Figure S10), as we previously showed that values on this axis show a consistent pattern of seasonal variation (Marsh et al., 2022). Year and MiSeq run were included as fixed factors, read depth as a covariate, and individual ID as a random factor to control for repeated measures.

## Results

### Gut parasites and their infection location

**Figure 1:**
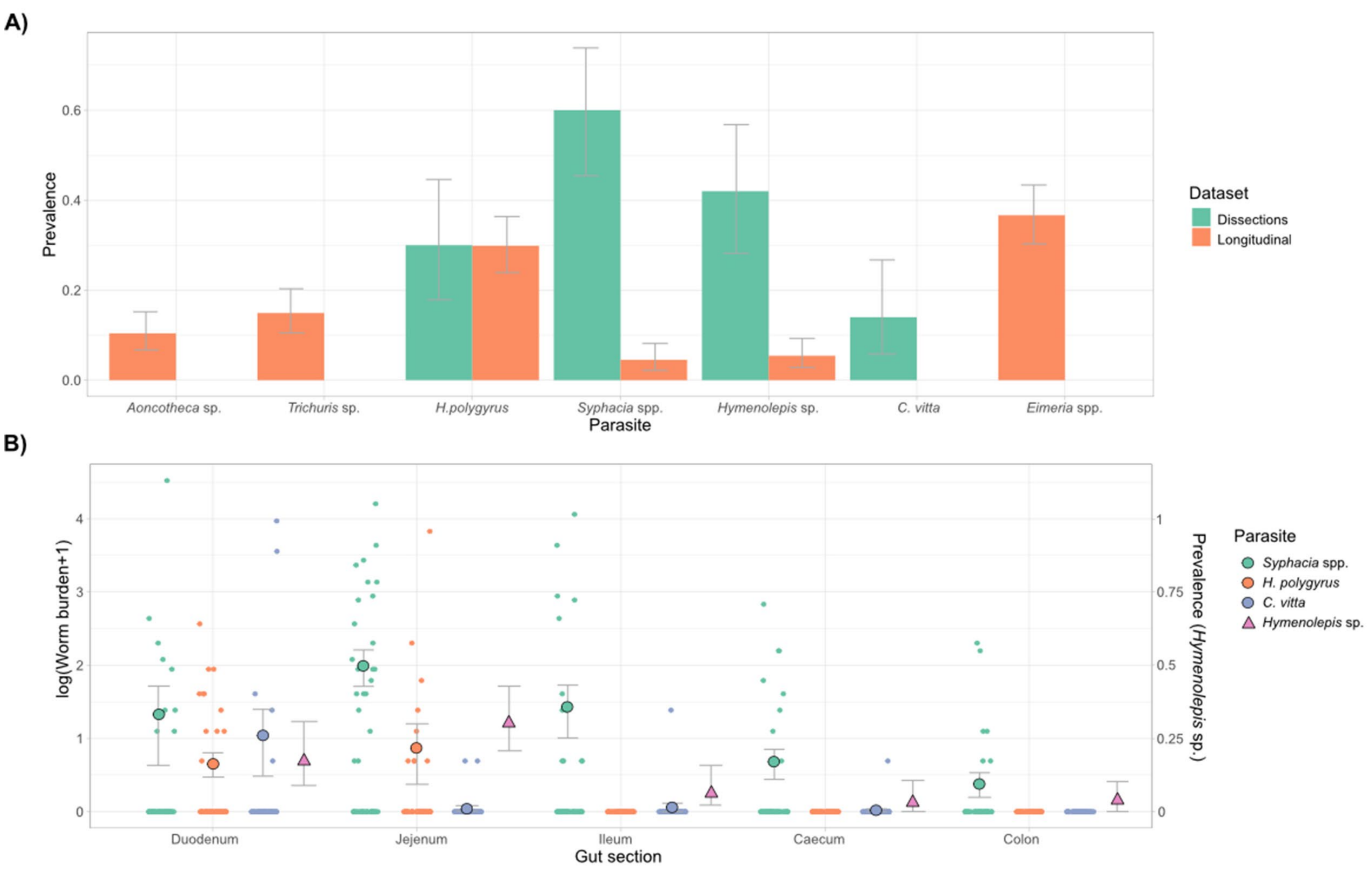
Summary of gut parasites found in the wood mice of Wytham woods. **(A)** Parasite prevalence and 95% confidence intervals for all six species of parasite detected, according to dataset/diagnostic method. The non-invasive dataset was collected through fecal flotations on 221 fecal samples collected from live-trapped mice over a 3-year period (2015–18), and the dissection dataset included 50 mice destructively sampled in 2017–2018 from a nearby area within the same woodland. **(B)** Detection of gut parasites in the dissection study across different sections of the GI tract (duodenum, jejunum, ileum, caecum, and colon). Worm burdens of *Syphacia* spp., *H. polygyrus* and *C. vitta* are shown in relation to the y-axis on the left-hand side, while the prevalence of *Hymenolepis* sp. proglottids per gut section is shown in relation to the *y*-axis on the right-hand side (as burdens could not be quantified for this species). Mean and standard error of worm burdens are shown, as well as prevalence (95% CI’s) of *Hymenolepis* sp.

Overall, four types of parasitic nematode were detected in this population: *Heligmosomoides polygyrus*, *Syphacia* spp., *Aoncotheca* sp., and *Trichuris muris*. Additionally, one cestode (*Hymenolepis* sp.), one trematode (*Corrigia vitta*), and *Eimeria* spp. protozoa were also detected (Figure 1A). Differences in our ability to detect these parasites by dissection and fecal flotation were apparent for several species (Figure 1A). For example, *Syphacia* pinworm were rarely detected in feces but were common in dissections, *C. vitta* was only detected in dissections (likely because their eggs were too heavy to float with our flotation solution), while *Aoncotheca* sp., *T. muris* and *Eimera* spp. were only detected in fecal flotation but not in dissections. These two diagnostics methods therefore had variable, and species-specific sensitivity. *Aoncotheca* sp. may have been present in the stomach lining, which was not inspected during dissections. Of the dissected mice, 90% harbored at least one parasite, with 36% co-infected by more than one parasite and most co-infected individuals (83%) showing co-infection within a gut section. Fecal flotations for the non-invasive study showed a lower overall infection rate, with 66% faecal samples containing at least one type of parasite and 24% containing two or more (mean = 1.02 ± 0.064 parasites per sample, range 0–4).

Of the four helminths detected internally, *H. polygyrus* was the most localized and found in the duodenum and jejunum, with similar burdens in each (Figure 1B). *Hymenolepis* sp. burdens could not be measured as we did not find scolexes, though their proglottids were mostly found in the jejunum (Figure 1B). The trematode *C. vitta* was most abundant in the duodenum, but also occasionally found further along the GI tract (Figure 1B). *Syphacia* spp. were found all along the GI tract but burdens were higher in the small intestine than the caecum and colon (Figure 1B). We identified two species of *Syphacia*, with *S. stroma* mostly found in the small intestine and *S. frederici* largely confined to the caecum and colon, consistent with previous studies on these species (Stewart et al., 2018), though both species were detected to some extent in all gut sections (Supplementary Figure S3). As *Syphacia* were too abundant to identify every individual to species level, they were grouped as *Syphacia* spp. for subsequent statistical analyses. There were no strong correlations in the counts of each parasite across gut sections (Supplementary Figure S4) or among parasites within each gut section (Supplementary Figure S5).

### Local and distant effects of helminth infection on the gut microbiota

**Figure 2:**
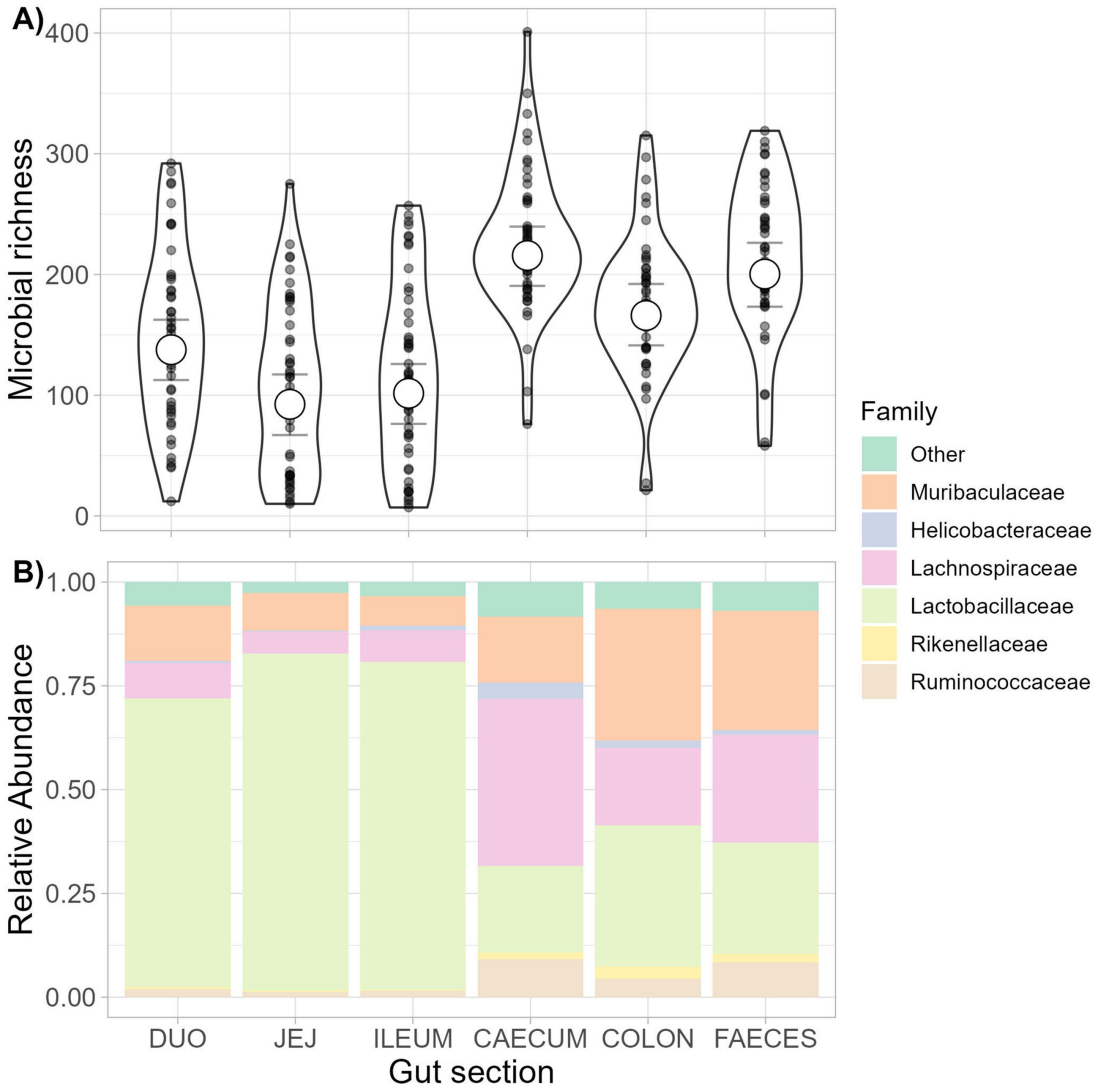
Richness and composition of the microbiota along the gastrointestinal tract. The richness and composition of the gut microbiota in different sections of the gastrointestinal (GI) tract were estimated from a dissection study of wild wood mice (*n* = 50). Samples were taken from the 5 gut sections (duodenum, jejunum, ileum, caecum, and colon) and fecal samples from the trap of the same mice for comparison. **(A)** Posterior means and 95% credible intervals from a Bayesian mixed model assessing variation in community richness along the GI tract and feces, with raw data points shown. **(B)** The family-level composition of the bacterial community along the GI tract and feces. Relative abundance of the top 5 most abundant families is shown, with less abundant families grouped as ‘other’.

Estimated microbiota richness varied across gut sections (Figure 2A and Supplementary Figure S6), with richness highest in the large intestine (especially the caecum), and lower in the small intestine (Supplementary Table S2). However, average microbiota richness was not predicted by infection status for *Syphacia* spp., *Hymenolepis* sp., *H. polygyrus,* or *C. vitta* within any gut section or across the whole gut (no gut section: parasite presence interaction terms were significant; Supplementary Table S2). Furthermore, helminth infection status did not predict duodenal or colonic microbial richness, irrespective of whether parasites were present in the same gut section or not (Supplementary Figure S7). However, estimated microbiota richness was higher in both the caecum [78.59 more ASVs, 95% CI 11.06 to 146.34] and ileum [102.52 more ASVs, 95% CI 47.47 to 158.12] when *C. vitta* was present in other, mostly upstream, gut sections (Supplementary Figure S7). Microbial richness in the jejunum was lower when *Hymenolepis* sp. was detected anywhere in the whole gut (61.04 fewer ASVs, 95% CI 10.60 to 112.47, Supplementary Figure S7). For those helminths whose variation in abundance allowed (*Syphacia* spp. and *H. polygyrus*), the inclusion of a logged helminth burden variable instead of presence/absence did not change the results (parameter estimate 95% CIs still included 0).

Although microbiota composition varied among gut sections (Supplementary Table S3 and Figure 2B), it was not predicted by overall infection status with any of the four helminth parasites found in the dissection study (Supplementary Table S3). Similarly, neither local nor non-local helminth presence predicted microbiota composition within any one gut section (Supplementary Table S3), and this remained unchanged when including the logged burden of *Syphacia* spp. and *H. polygyrus* instead of presence/absence (*p* > 0.05).

### Gut microbiota composition and comparison across sample types

The most abundant bacterial families in the dissection dataset were Lactobacillaceae (52.92%), Lachnospiraceae (17.72%), and Muribaculaceae (17.25%). The small intestine showed lower richness and was dominated by Lactobacillaceae compared to the large intestine, where Muribaculaceae and Lachnospiraceae were more abundant (Figure 2).

Before analyzing associations between parasites and the gut microbiota as assessed from faecal data in the non-invasive study, we first tested how well fecal microbiota profiles reflected internal microbiota profiles among dissected mice. Fecal microbiota richness was similar to that observed in the caecum and colon and higher than in the small intestine (Figure 2A and Supplementary Figure S6). Fecal microbiota composition was broadly similar to that of the colon at family level, while the caecum showed higher relative abundances of Lachnospiraceae (Figure 2B). At the ASV level, microbiota composition varied according to sample type within individuals [PERMANOVA; Gut section *F*_(5,267)_ = 11.93, *p* = 0.001, marginal *R*^2^ = 0.158]. However, differences in mean dissimilarity, dispersion, or both could contribute to this result as sample types varied in compositional dispersion [*F*_(5,277)_ = 19.15, *p* = 0.001].

As fecal samples appeared similar to the microbial composition of the colon (Figure 2B), we further assessed whether, despite being collected non-invasively, fecal samples can provide a reliable individual-specific gut microbiota profile. To do this, we tested whether within-individual microbiota similarity between the colon and feces outweighed microbiota similarity among samples of the same type (colon or feces) collected from different individuals. This was indeed the case with significantly lower Bray–Curtis dissimilarity between colon and fecal samples taken from the same individual than among samples of the same type (colon or feces) taken from different individuals (permutational Wilcoxon tests, colon-colon vs. colon–feces; *U* = 15,672, *p* < 0.001, feces–feces vs. colon–feces; *U* = 2,416, *p* < 0.001, Supplementary Figure S8). Individual ID also explained much more variance in microbiota composition than sample type in a PERMANOVA including paired colon and fecal samples for each individual [Gut section; *F*_(1,88)_ = 2.73, *p* = 0.001, marginal *R*^2^ = 0.014, Animal ID; *F*_(49,88)_ = 3.08, *p* = 0.001, marginal *R*^2^ = 0.766]. Differences in group dispersions were significant for both animal ID [*F*_(49,39)_ = 4.69, *p* = 0.001] and sample type [*F*_(1,87)_ = 6.16, *p* = 0.014], with colon samples showing higher dispersion than fecal samples. Procrustes analysis showed a high concordance in microbiota beta diversity patterns analyzed using fecal and internal colon samples (m_12_^2^ = 0.839, *p* = 0.001; Supplementary Figure S8), indicating that non-invasive fecal samples provide a good representation of the beta diversity patterns that would be obtained using invasive colonic samples.

### Parasite–microbiota associations in the non-invasive study

Of the longitudinal non-invasive samples, we analyzed 221 samples from 105 mice captured between 2015 and 2018 that had paired fecal microbiota profiles and parasite fecal flotation data. These mice were sampled between 1 and 8 times, with 54 mice sampled more than once and an average of 2.1 samples per mouse. Fecal samples had a mean of 44,828 reads after filtering (± 979 standard error), with an average of 207 (± 4.2 standard error) ASVs per sample. The dominant bacterial families were Muribaculaceae (37.83%), Lactobacillaceae (23.37%), and Lachnospiraceae (18.26%), with similar overall relative abundances to those found in fecal samples from the dissection study (Supplementary Table S4).

**Figure 3:**
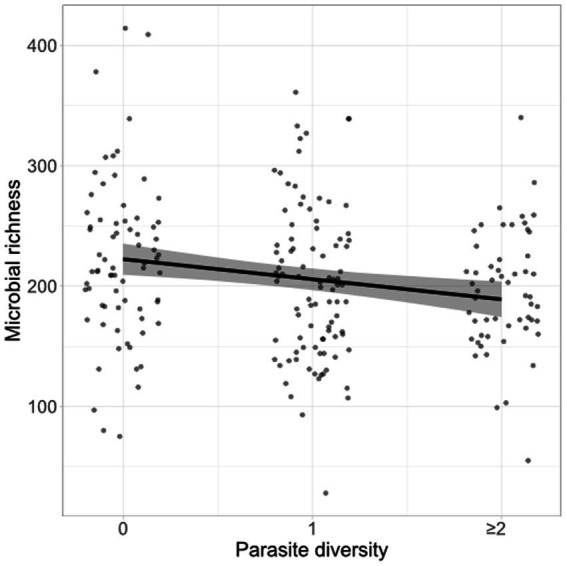
Fecal microbiota richness decreases with increased gut parasite richness. Fecal flotations and 16S sequencing were conducted to profile gut parasite burdens and the gut microbiota of wood mice from a longitudinal, non-invasive study (*n* = 221). A Bayesian mixed model was used to assess the relationship between the number of parasite species (0, 1, or > = 2) detected per fecal sample and the richness of the microbial community, after controlling for MiSeq run, read depth, and repeated measures per individual. Raw data points (jittered) as well as the conditional effect of parasite richness and its 95% credible intervals (shaded area) from the model are shown.

Microbiota richness was not predicted by infection status for any single parasite, though individuals infected with *T. muris* showed a tendency towards lower fecal microbiota diversity (posterior mean = −17.74, 95% CI -36.95 to 1.09; Supplementary Figure S9). While microbiota richness was not strongly associated with infection by any one parasite, it was negatively associated with the total number of parasite species detected in a fecal sample (posterior mean = −16.72, 95% CI –27.31 to −6.32, *n* = 221 mice; Figure 3). For comparison, fecal microbial richness in the dissection dataset was not found to be significantly predicted by the total number of parasite species detected, though the sample size was much smaller for this analysis (posterior mean = 0.85, 95% CI –18.42 to −20.33, *n* = 42 mice, Supplementary Table S5).

Microbiota composition was not associated with the burden of any one parasite detected in feces, though *Eimeria* infection showed a near significant association [*F*_(1, 102)_ = 1.73, *p* = 0.065; Supplementary Table S6]. There was also no association between overall parasite community composition and microbiota composition (Mantel test *r* = 0.019, *p* = 0.126, *n* = 148).

### Seasonal dynamics of parasites and the fecal microbiota

The burden of *Eimeria* spp. oocysts and *T. muris* eggs in feces both significantly predicted microbiota PC1 (values on the seasonally shifting primary axis of a Bray–Curtis PCoA, from Marsh et al., 2022; Supplementary Figure S10) in a GAMM modelling seasonality in this variable (Supplementary Table S7). Despite this, the explanatory power of the seasonal (day of year) term in this model did not change appreciably when parasite variables were included in the model (day of year adjusted R^2^ = 0.485 vs. *R*^2^ = 0.462 in models including and excluding parasite variables respectively), and neither *T. muris* nor *Eimeria*. spp. showed any significant pattern of seasonal variation in burdens [*T. muris* GAMM, s(day of year) *F* = 0.00, *p* = 0.458; *Eimeria* spp. GAMM, s(day of year) *F* = 0.00, *p* = 0.847; Supplementary Figure S11], suggesting changes in gut parasitic infections cannot explain previously documented seasonal variation in the gut microbiota in this population.

## Discussion

Interactions between gut parasites and the microbiota are known to occur through a variety of mechanisms and can have important implications for host health and disease (Leung et al., 2018b). Much of this mechanistic understanding has been gained from laboratory-based studies often using mice and model helminths, with few observational studies on wild mammalian populations conducted so far. Here, we combined two approaches to examine correlational evidence for parasite-microbiota interactions in a natural population of wild wood mice. A dissection study examined local and distant parasite–microbiota associations along the GI tract, and a longitudinal, non-invasive study examined the relative importance of parasite infection as a potential driver of microbiota community dynamics. Overall, we found that natural parasite infections in our study population showed some association with gut microbiota alpha diversity, but we found very little evidence for parasite associations with composition (beta diversity).

### No strong associations between parasite infection and microbiota composition

We found no associations between overall fecal microbiota composition and infection by any of the parasites detected, though *Eimeria* spp. infection and *T. muris* infection did predict the major compositional axis of variation (PC1), independent of previously documented seasonal variation in this variable (Supplementary Table S7). Our finding of a lack of association between parasitic infections and overall microbiota composition differs from previous studies on related host species. For instance, Kreisinger et al. (2015) detected a variety of local and distant associations between gut helminths and microbiota beta diversity along the GI tract in a closely related mouse species (*Apodemus flavicollis*), the strongest being between infection with the tapeworm *Hymenolepis* spp. and increased Bacteroidetes S24-7 (Muribaculaceae) in the stomach. A previous non-invasive study on wood mice also detected associations between nematode infection (largely *H. polygyrus*) increased relative abundance of a Lachnospiraceae genus and decreased relative abundance of *Escherichia* in feces (Maurice et al., 2015). Several differences exist between the studies which could explain these discrepancies. Notably, wood mice in the current study harbored comparatively few parasites overall (Behnke et al., 1999; Kreisinger et al., 2015), and the stomach region, where effects were detected previously in *Apodemus* (Kreisinger et al., 2015), was not included in this study.

In the longitudinal non-invasive study, infection with *Eimeria* spp. and to a lesser extent, *T. muris* was associated with variation in the first axis of a PCA (PC1), which explained 12.89% of the variation in fecal microbiota composition and has previously been shown to display reproducible seasonal dynamics (Marsh et al., 2022; Maurice et al., 2015). However, these parasite effects were independent of seasonal variation in PC1, and neither *T. muris* nor *Eimeria* spp. showed consistent seasonal patterns in our 3-year dataset. These two observations together that infections by these parasites are very unlikely to be key drivers of the previously documented repeatable seasonal shifts in gut microbiota composition among UK wood mice.

### Microbiota richness is associated with specific parasite infections and coinfections

We found stronger evidence for associations between parasite infection and microbiota alpha diversity (richness) in both the dissection and non-invasive datasets. Microbial richness in the ileum and caecum was positively associated with the abundance of the trematode *C. vitta* elsewhere in the GI tract, suggesting an indirect mechanism of interaction. *C. vitta* was mostly found in the duodenum and more rarely further downstream. In fact, this is a parasite of the interlobary canals of the pancreas where in heavy infections it can disrupt the flow of pancreatic juices into the GI tract (Lewis et al., 2021; Magee et al., 1993). This may provide a potential mechanism underlying the association between *C. vitta* infection and increased bacterial diversity in the caecum, as pancreatic juices show antimicrobial activity to prevent overgrowth of pathogenic bacteria (Zhang et al., 2022) and bile has myriad impacts on the microbiota (Collins et al., 2023) that could influence diversity if bile flow into the gut was affected by *C. vitta* infection. We also detected a weaker negative association between infection by the tapeworm *Hymenolepis* sp. and microbial diversity in the jejunum. Previous studies have detected associations between *Hymenolepis* sp. infection and the abundance of specific bacterial taxa (Kreisinger et al., 2015; Aivelo and Norberg, 2018), yet it remains unclear what the mechanism underlying such associations might be. Infection with the nematode *T. muris* was associated with a (non-significant) reduction in fecal microbial richness in the non-invasive study. Although *T. muris* was not detected in the dissection study and therefore we cannot say if this association occurred locally or not, this finding is consistent with two previous *T. muris* experimental infection studies, which detected an infection-driven decline in microbiota diversity in lab mice (Holm et al., 2015; Houlden et al., 2015).

In addition to these specific associations with individual parasite species, an overall association between coinfection with multiple parasites (mostly helminths) and reduced microbial diversity was found in the non-invasive study (Figure 3). The relationship between co-infection with multiple species of parasites and the microbiota has rarely been considered, though Cooper et al. (2013) found reduced human fecal microbiota diversity in mixed infections of *Trichuris trichiura* and *Ascaris lumbricoides* compared to single species infections, and a recent meta-analysis also identified a trend towards reduced gut microbial diversity in infected vs. uninfected rodents (Scotti et al., 2020). The overall negative association of gut parasitism with microbiota diversity found here could arise through various mechanisms, including the secretion of antimicrobial compounds by gut parasites (Cotton et al., 2012), increased susceptibility to parasitic infection of mice with less diverse gut microbiota (Buffie and Pamer, 2013), or both. Since our data are purely correlational, further study would be needed to determine whether these associations reflect causal relationships, and if so what their direction and underlying mechanisms might be.

### Combining methodological approaches can provide valuable insight

We provided validation of trap-collected feces as a representative, non-invasive sample of the microbiota in the large intestine which captures reliable individual-specific gut microbiota profiles. Colon samples were more variable among individuals than fecal samples, suggesting a slight loss of this individual specificity with non-invasive methods. This is consistent with previous studies which have highlighted that signatures of host evolution are better retained when studying intestinal mucosal samples vs. fecal samples (Ingala et al., 2018), and that the fecal microbiota represents a subset of the endogenous microbiota (Kohl et al., 2015). Despite this slight loss of individuality, fecal samples were still highly representative of individual differences in gut microbiota composition, with individual identity explaining the majority of variation among fecal microbiota samples.

Study to understand how common gut parasitic infectious might shape the gut microbiota and vice versa is still very much in its infancy, and synthesis and consensus across studies are yet to emerge (Cortés et al., 2019; Scotti et al., 2020). Some study designs are likely to yield more fruitful inference than others in the future study in this area. Longitudinal study designs where individuals are repeatedly sampled over time and key covariates are controlled for should offer more power than cross-sectional studies to detect important interactive effects between parasites and the gut microbiota. They can also enable a better understanding of specific ecological interactions among gut inhabitants, by investigating the temporal covariation in abundance among pairs of taxa to infer positive or negative associations (Roche et al., 2023). Additionally, experimental approaches (e.g., parasite treatment experiments) in tractable wild systems could increase our understanding of causal relationships further (Davidson et al., 2020; Fenton et al., 2014). Such an experimental approach was recently used effectively to test how helminthic infection altered the gut microbiota in African buffalo, revealing complex co-infection-dependent impacts (Sabey et al., 2021). Future studies of this nature in other tractable systems like rodents would be highly valuable to understand what underpins patterns of association between parasites and the microbiota such as those detected here, as well as what implications they may have for host physiology, immunity, and ultimately fitness.

## Data Availability

All raw sequence data associated with this study is deposited in the European Nucleotide Archive under the accession PRJEB49639. In addition, the processed ASV count and taxonomy tables along with the metadata for the longitudinal and dissection datasets are available as supplementary material. The R code used for the analyses presented here are also available as Supplementary material.
