# Supplementary material for: Parasite–gut microbiota associations in wild wood mice (Apodemus sylvaticus)
Source: Front Microbiol. 2024 Nov 18;15:1440427. doi: 10.3389/fmicb.2024.1440427 (PMC11608965; doi:10.3389/fmicb.2024.1440427)
Supplement: Supplementary file 1 [file Data_Sheet_1.zip › 1440427_Knowles_Rcode.DOCX]

Mouse_parasite_long_daticrobe_interactions_analyses

K Marsh

24/11/2023

This Markdown contains all the statistical analyses associated with the manuscript Marsh et al. Parasite-Microbe associations in wild wood mice (in prep).

# 1. Prep data and libraries

library(phyloseq)
library(microbiome)
library(tidyverse)
library(iNEXT)
library(forcats)
library(plyr);library(dplyr)
library(reshape2)
library(DescTools)
library(gridExtra)
library(cowplot)
library(ggpubr)
library(breakaway)
library(MuMIn)
library(lme4)
library(lmerTest)
library(vegan)
library(brms)
library(loo)
library(pairwiseAdonis)
library(decontam)

##### load data
#ASV table
seqtab <- readRDS('ASV_count_table.rds')#ASV table
row.names(seqtab)<-sapply(strsplit(row.names(seqtab), "_"), `[`, 1) #change WE names
row.names(seqtab)<-sapply(strsplit(row.names(seqtab), "-"), `[`, 1) #change WD names
#tax table
taxtab <- readRDS('ASV_taxonomy_table.rds')#taxonomy
#metadata per dataset
dissection_dat <- read.csv('Wytham_dissection_sample_data.csv')#full metadata for dissection study
long_dat <- read.csv('Wytham_longitudinal_sample_data.csv')#full metadata for longitudinal study


master_phy <- phyloseq(otu_table(seqtab, taxa_are_rows = F), tax_table(taxtab))#
Sequence_sample_code <- sample_names(master_phy)
master_meta <- as.data.frame(Sequence_sample_code)
master_meta$readDepth <- sample_sums(master_phy)# add sample-wise read depth to metadata
rownames(master_meta) <- master_meta$Sequence_sample_code
master_phy <- phyloseq(otu_table(otu_table(master_phy)), tax_table(tax_table(master_phy)), sample_data(master_meta))

#change ASV names to something nicer to read, and store original full sequences for each ASV in ref.seq slot
dna <- Biostrings::DNAStringSet(taxa_names(master_phy))
names(dna) <- taxa_names(master_phy)
master_phy<- merge_phyloseq(master_phy, dna)
taxa_names(master_phy) <- paste0("ASV", seq(ntaxa(master_phy)))

Phyloseq preprocessing

Filter out non-gut microbial taxa and contaminants

##### Filter non-gut microbial taxa
master_phy <- subset_taxa(master_phy, Kingdom!="Eukaryota" & Kingdom!="Archaea" &
 Phylum!="NA" & Phylum!="Cyanobacteria" &
 Class!="Chloroplast" &
 Family!="Mitochondria")
#get rid of taxa not found in any of these samples
master_phy <- prune_taxa(taxa_sums(master_phy) > 0, master_phy)#9807 taxa 1263 samples

##### remove contaminants using negative controls
#Indicator for Negative Samples
neg_samps <- c("WD192", "WD288", "WD384", "WD96", "WD480")
sample_data(master_phy)$is.neg <- ifelse(sample_data(master_phy)$Sequence_sample_code%in%neg_samps,TRUE,FALSE)

contamdf.prev <- isContaminant(master_phy, method="prevalence", neg="is.neg", threshold=0.1)#default threshold
#table(contamdf.prev$contaminant) # 159 identified

# Clean Contaminants Out
master_phy <- prune_taxa(contamdf.prev$contaminant==FALSE, master_phy)#9648 taxa

Sample-wise filtering using iNEXT analyses

##
#what does read depth across samples look like before filtering?
# summary(sample_data(master_phy)$readDepth)
# taxa_are_rows(master_phy)
#otu_tab <- as.data.frame(otu_table(master_phy))
#otu_tab <- t(otu_tab) #make sure sample are columns and species are rows

#run iNEXT
#master_phy_q0 <- iNEXT(otu_tab, q=0, datatype="abundance")#takes a long time - save output!
#saveRDS(out0, 'Mouse_microbiome_iNEXT_output.rds')
#master_phy_q0 <- readRDS('Mouse_microbiome_iNEXT_output.rds')
#
# #plot curves
# ggiNEXT(master_phy_q0, type = 1, color.var = "none") +
# theme(legend.position="none") +
# ggtitle("iNEXT sample-sized based rarefaction/extrapolation curve \n Full 16S dataset") +
# xlim(0,15000)
#
# ggiNEXT(master_phy_q0, type = 2, color.var = "none") +
# theme(legend.position="none") +
# ggtitle("iNEXT sample completedness curve \n Full 16S dataset") +
# xlim(0,8300)

#prune samples where read depth < 8000
master_phy.prune <- prune_samples(sample_sums(master_phy)>=8000, master_phy)
master_phy.prune <- prune_taxa(taxa_sums(master_phy.prune) > 0, master_phy.prune)
#master_phy.prune #### Use for alpha diversity analyses

## OTU prevalence/abundance filtering
#criteria; keep taxa with > 1 copy in at least 1% samples (removes any OTUs that weren't present in at least than 1% samples with a copy number more than 1 - which will remove singletons)
master_phy.prune.filt = filter_taxa(master_phy.prune, function(x) sum(x > 1) > (0.01*length(x)), prune=TRUE)
#master_phy.prune.filt #### Use for beta diversity analyses

Create separate phyloseq objects for each dataset

master_meta <- as(sample_data(master_phy.prune.filt), 'data.frame')

#dissection data
dissection_dat$Sequence_sample_code[dissection_dat$Sequence_sample_code==''] <- NA
dissection_dat <- subset(dissection_dat, !is.na(Sequence_sample_code))
dissection_dat$Tapeworm_present[dissection_dat$Tapeworm_present==""] <- NA
dissection_dat$Tapeworm_present[dissection_dat$Tapeworm_present=="Y"] <- 1
dissection_dat$Tapeworm_present[dissection_dat$Tapeworm_present=="N"] <- 0
dissection_dat$Syphacia_pa <- ifelse(dissection_dat$Syphacia_count>0, 1, 0)
dissection_dat$Hpoly_pa <- ifelse(dissection_dat$Hpoly_count>0, 1, 0)
dissection_dat$Trematode_pa <- ifelse(dissection_dat$Trematode_count>0, 1, 0)
dissection_dat$Gut_section <- as.factor(dissection_dat$Gut_section)
dissection_dat$Sex <- as.factor(dissection_dat$Sex)
dissection_dat$Sex[dissection_dat$Sex==""] <- NA
dissection_dat$Trap_location <- as.factor(dissection_dat$Trap_location)
dissection_dat$Animal_number <- as.factor(dissection_dat$Animal_number)
#summary(as.factor(dissection_dat$Collection_date))

dissection_dat2 <- merge(dissection_dat, master_meta, by="Sequence_sample_code")
#rescale read depth to avoid scaling issues
dissection_dat2$readDepth_z <- (dissection_dat2$readDepth - mean(dissection_dat2$readDepth))/sd(dissection_dat2$readDepth)
dissection_dat2$Miseq_run <- as.factor(dissection_dat2$Miseq_run)
rownames(dissection_dat2) <- dissection_dat2$Sequence_sample_code

dissection_phy.alpha <- phyloseq(sample_data(dissection_dat2),
 tax_table(tax_table(master_phy.prune)),
 otu_table(otu_table(master_phy.prune)))
dissection_phy.alpha <- prune_taxa(taxa_sums(dissection_phy.alpha) > 0, dissection_phy.alpha)

dissection_phy.beta <- phyloseq(sample_data(dissection_dat2),
 tax_table(tax_table(master_phy.prune.filt)),
 otu_table(otu_table(master_phy.prune.filt)))
dissection_phy.beta <- prune_taxa(taxa_sums(dissection_phy.beta) > 0, dissection_phy.beta)


#longitudinal data
long_dat <- long_dat[,c(2:30,79:107)]
long_dat$XPIT_tag <- as.factor(long_dat$XPIT_tag)
levels(long_dat$XPIT_tag)[levels(long_dat$XPIT_tag)==''] <- NA
#length(levels(long_dat$XPIT_tag))
long_dat$Sex[long_dat$Sex%in%c("F?", "M?", "")] <- NA
long_dat$Reprod <- long_dat$Reprod%>%
 fct_collapse(active = c("IMP_NIPP","LACT", "NIPP", "PERF",
 "Perf, plug","PREG", "PREG/LACT",
 "Preg?","PREG?", "Preg? Imp",
 "TL", "TL+", "NIPP_PREG", "Perf, plug",
 "PERF_NIPP"),
 inactive = c("TA", "TA?","TS", "IMP", "IMP?",
 "TS?"))
levels(long_dat$Reprod)[levels(long_dat$Reprod)==''] <- NA
long_dat$Body_condition <- as.factor(long_dat$Body_condition)
long_dat$Age <- long_dat$Age %>% fct_collapse(J = c("J", "J?"), SA=c("SA", "SA?"))
levels(long_dat$Age)[levels(long_dat$Age)==''] <- NA
long_dat$Collection_date <- as.Date(long_dat$Collection_date, format= "%d/%m/%Y") #now collection date is recognised as in date format
long_dat$Day_of_year <- format(long_dat$Collection_date, "%j")#now get day of the year
long_dat$Day_of_year <- as.numeric(long_dat$Day_of_year)
long_dat <- subset(long_dat, Species=="AS" & Grid=="Main")

#get presence/absence per parasite
long_dat$Capillaria_pa <- ifelse(long_dat$Capillaria_EPG>0,1,0)
long_dat$Trichuris_pa <- ifelse(long_dat$Trichuris_EPG>0,1,0)
long_dat$Hpoly_pa <- ifelse(long_dat$Hpolygyrus_EPG>0,1,0)
long_dat$Syphacia_pa <- ifelse(long_dat$Syphacia_EPG>0,1,0)
long_dat$hymenolepid_pa <- ifelse(long_dat$Hymenolepid_EPG>0,1,0)
long_dat$Eimeria_EPG_sum <- long_dat$Eapionodes_EPG + long_dat$Ehungariensis_EPG +
 long_dat$Eimeria_sp_EPG + long_dat$Euptoni_EPG
long_dat$Eimeria_pa <- ifelse(long_dat$Eimeria_EPG_sum>0,1,0)
long_dat$parasite_div <- long_dat$Capillaria_pa + long_dat$Trichuris_pa + long_dat$Hpoly_pa +
 long_dat$Syphacia_pa + long_dat$Eimeria_pa + long_dat$hymenolepid_pa

long_dat2 <- merge(long_dat, master_meta, by="Sequence_sample_code")
rownames(long_dat2) <- long_dat2$Sequence_sample_code
long_dat2$readDepth_z <- (long_dat2$readDepth - mean(long_dat2$readDepth))/sd(long_dat2$readDepth)

#make phyloseq objects
long_phy.alpha <- phyloseq(sample_data(long_dat2),
 tax_table(tax_table(master_phy.prune)),
 otu_table(otu_table(master_phy.prune)))
long_phy.alpha <- prune_taxa(taxa_sums(long_phy.alpha) > 0, long_phy.alpha)

long_phy.beta <- phyloseq(sample_data(long_dat2),
 tax_table(tax_table(master_phy.prune.filt)),
 otu_table(otu_table(master_phy.prune.filt)))
long_phy.beta <- prune_taxa(taxa_sums(long_phy.beta) > 0, long_phy.beta)

#2. Parasites found in Wytham

## Dissection dataset summary of parasites found

Per gut section:

#summarise worm burdens across gut sections#
dissection_dat$Tapeworm_present <- as.numeric(dissection_dat$Tapeworm_present)

worm_section_sum <- ddply(dissection_dat, c("Gut_section"), summarise,
 syphacia_mean = mean(Syphacia_count),
 syphacia_se = sd(Syphacia_count) / sqrt(length(Syphacia_count)),
 hpoly_mean = mean(Hpoly_count),
 hpoly_se = sd(Hpoly_count) / sqrt(length(Hpoly_count)),
 trem_mean = mean(Trematode_count),
 trem_se = sd(Trematode_count) / sqrt(length(Trematode_count)),
 tape_sum = sum(Tapeworm_present)
 )
#worm_section_sum

worm_section_means <- worm_section_sum[,c(1,2,4,6)]
colnames(worm_section_means) <- c("Gut_section", "Syphacia spp.", "H.polygyrus", "C.vitta")
worm_section_means <- melt(worm_section_means, id.vars = "Gut_section", variable.name = "Parasite", value.name = "Mean")

worm_section_se <- worm_section_sum[,c(1,3,5,7)]
colnames(worm_section_se) <- c("Gut_section", "Syphacia spp.", "H.polygyrus", "C.vitta")
worm_section_se <- melt(worm_section_se, id.vars = "Gut_section", variable.name = "Parasite", value.name = "SE")

worm_section_sum2 <- merge(worm_section_means, worm_section_se, by=c("Gut_section", "Parasite"))
worm_section_sum2$upper_se <- worm_section_sum2$Mean + worm_section_sum2$SE
worm_section_sum2$lower_se <- worm_section_sum2$Mean - worm_section_sum2$SE
worm_section_sum2 <- worm_section_sum2[,c(1,2,3,5,6)]

# add tapeworm prevalence (calc CI's)
tapeworm_dat <- worm_section_sum[,c(1,8)]
names(tapeworm_dat) <-c("Gut_section", "Count")
tape_prevs <- as.data.frame(BinomCI(x=tapeworm_dat$Count, n=50, method="clopper-pearson"))
tape_prevs$Gut_section <- c("CAECUM", "COLON", "DUO", "ILEUM", "JEJ", "POO")
tape_prevs$Parasite <- "Hymenolepis sp."
names(tape_prevs) <- c("Mean", "lower_se", "upper_se", "Gut_section", "Parasite")
tape_prevs$Mean <- tape_prevs$Mean*100
tape_prevs$lower_se <- tape_prevs$lower_se*100
tape_prevs$upper_se <- tape_prevs$upper_se*100

#reshape raw data to long format; gut section, parasite, count
worm_sum_reshape <- dissection_dat[,c(34,36,40,41,43)]
worm_sum_reshape$Tapeworm_present[worm_sum_reshape$Tapeworm_present==1] <- 100
colnames(worm_sum_reshape) <- c("Gut_section", "Syphacia spp.","Hymenolepis sp.", "H.polygyrus", "C.vitta")
worm_sum_reshape <- melt(worm_sum_reshape, id.vars=c("Gut_section"), variable.name = "Parasite", value.name = "Count")
worm_sum_reshape$Count <- as.numeric(worm_sum_reshape$Count)

worm_section_sum3 <- rbind(worm_section_sum2, tape_prevs)
worm_sum_reshape$Gut_section <- factor(worm_sum_reshape$Gut_section, levels=c("DUO", "JEJ", "ILEUM", "CAECUM", "COLON"))
worm_section_sum3$Gut_section <- factor(worm_section_sum3$Gut_section, levels=c("DUO", "JEJ", "ILEUM", "CAECUM", "COLON"))
worm_sum_reshape$Parasite <- factor(worm_sum_reshape$Parasite, levels=c("Syphacia spp.","H.polygyrus", "C.vitta", "Hymenolepis sp."))
worm_section_sum3$Parasite <- factor(worm_section_sum3$Parasite, levels=c("Syphacia spp.","H.polygyrus", "C.vitta", "Hymenolepis sp."))

#plot raw points
worm_section_plot_raw <- ggplot( ) +
 geom_point(data=subset(worm_sum_reshape, Gut_section!="POO" & Parasite!="Hymenolepis sp."),
 aes(x=Gut_section, y=log(Count+1), fill=Parasite, colour=Parasite), position=position_jitterdodge())
worm_section_plot_raw

#add mean and se
worm_section_plot_means <- worm_section_plot_raw +
 geom_errorbar(data=subset(worm_section_sum3, Gut_section!="POO"),
 aes(x=Gut_section, ymax=log(upper_se+1), ymin=log(lower_se+1), fill=Parasite),
 position = position_dodge2(), colour="darkgrey", width=0.8) +
 geom_point(data=subset(worm_section_sum3, Gut_section!="POO"),
 aes(x=Gut_section, y=log(Mean+1), fill=Parasite),
 colour="black", shape=21, size=4, position=position_jitterdodge())
worm_section_plot_means

#add aesthetics
worm_section_final <- worm_section_plot_means + theme_light() + xlab("Gut section") +
 theme(axis.title = element_text(size=15), axis.text = element_text(size=12),
 legend.text = element_text(size=12), legend.title = element_text(size=15), strip.text = element_text(size=12)) +
 scale_colour_brewer(palette = "Set2") + scale_fill_brewer(palette = "Set2") +
 # Add second axis for tapeworm prevalence
 scale_y_continuous( name = "Worm burden", sec.axis = sec_axis( trans=~exp(.)-1, name="Prevalence (Hymenolepis sp.)") )

worm_section_final

Overall prevalence of parasites across animals

worm_total_sum <- ddply(subset(dissection_dat, Gut_section!="POO"), c("Animal_number"), summarise,
 syphacia_sum = sum(Syphacia_count),
 tapeworm_sum = sum(Tapeworm_present),
 hpoly_sum = sum(Hpoly_count),
 trem_sum = sum(Trematode_count)
 )

#summarise within-individual parasite diversity
worm_total_sum$parasite_diversity <- worm_total_sum$syphacia_sum + worm_total_sum$tapeworm_sum + worm_total_sum$hpoly_sum + worm_total_sum$trem_sum
#hist(worm_total_sum$parasite_diversity)
#nrow(subset(worm_total_sum, parasite_diversity>0))/50
#nrow(subset(worm_total_sum, parasite_diversity>1))/50

####(try to improve below code using apply functoins!?)
#convert to presence/absence
worm_total_sum$syphacia_sum[worm_total_sum$syphacia_sum>0] <- 1
worm_total_sum$tapeworm_sum[worm_total_sum$tapeworm_sum>0] <- 1
worm_total_sum$hpoly_sum[worm_total_sum$hpoly_sum>0] <- 1
worm_total_sum$trem_sum[worm_total_sum$trem_sum>0] <- 1

syphacia_count <- sum(worm_total_sum$syphacia_sum)
tape_count <- sum(worm_total_sum$tapeworm_sum)
hpoly_count <- sum(worm_total_sum$hpoly_sum)
trem_count <- sum(worm_total_sum$trem_sum)

worm_prevs_dissection <- data.frame(Parasite=c("Syphacia spp.", "Hymenolepis sp.",
 "H.polygyrus", "C.vitta"),
 Count=c(syphacia_count, tape_count,
 hpoly_count,trem_count))

worm_prevs_dissection2 <- cbind(worm_prevs_dissection, as.data.frame(BinomCI(x=worm_prevs_dissection$Count, n=50, method="clopper-pearson")))
worm_prevs_dissection2$Dataset <- "Dissections"

Correlations of parasites among/within gut sections

#convert dissection df to long format; mouse, gut section, parasite, abundance
diss_meta <- as(sample_data(dissection_phy.beta), 'data.frame')
diss_meta$Tapeworm_present <- as.factor(diss_meta$Tapeworm_present)
diss_long <- diss_meta %>% select(Animal_number, Gut_section, Syphacia=Syphacia_count, Hpolygyrus=Hpoly_count, Cvitta=Trematode_count,
 Hymenolepis=Tapeworm_present) %>%
 pivot_longer(cols = c("Syphacia", "Hpolygyrus", "Cvitta", "Hymenolepis"), names_to = "Parasite", values_to = "Abundance")

#same parasite across gut sections:
#plots per parasite, facetted by gut section combo
level_order <- c("DUO", "JEJ", "ILEUM", "CAECUM", "COLON", "POO")
diss_meta$Gut_section <- factor(diss_meta$Gut_section, levels = level_order)

syphacia_plots <- diss_meta %>% select(Animal_number, Gut_section, Syphacia=Syphacia_count) %>% filter(Gut_section!="POO") %>%
 pivot_wider(id_cols = "Animal_number", names_from = "Gut_section", values_from = "Syphacia") %>%
 relocate(DUO, .after = Animal_number) %>%
 GGally::ggpairs(columns = 2:6, title = "A) Syphacia counts", upper = "blank", switch = "both") +
 theme(axis.text.x = element_text(angle = 45))
Hpoly_plots <- diss_meta %>% select(Animal_number, Gut_section, Hpolygyrus=Hpoly_count) %>% filter(Gut_section!="POO") %>%
 pivot_wider(id_cols = "Animal_number", names_from = "Gut_section", values_from = "Hpolygyrus") %>%
 relocate(DUO, .after = Animal_number) %>%
 GGally::ggpairs(columns = 2:6, title = "B) H. polygyrus counts", upper = "blank", switch = "both")+
 theme(axis.text.x = element_text(angle = 45))
Cvitta_plots <- diss_meta %>% select(Animal_number, Gut_section, Cvitta=Trematode_count) %>% filter(Gut_section!="POO") %>%
 pivot_wider(id_cols = "Animal_number", names_from = "Gut_section", values_from = "Cvitta") %>%
 relocate(DUO, .after = Animal_number) %>%
 GGally::ggpairs(columns = 2:6, title = "C) C. vitta counts", upper = "blank", switch = "both")+
 theme(axis.text.x = element_text(angle = 45))

# ggsave2("Syphacia_abundance_across_gut_sections.jpeg",syphacia_plots, height = 7, width = 9)
# ggsave2("Hpoly_abundance_across_gut_sections.jpeg",Hpoly_plots, height = 7, width = 9)
# ggsave2("Cvitta_abundance_across_gut_sections.jpeg",Cvitta_plots, height = 7, width = 9)


#different parasites within gut sections:
#plots per gut section, facetted by parasite combo

duo_plots <- diss_meta %>% filter(Gut_section=="DUO") %>%
 select(Animal_number, Syphacia=Syphacia_count, Hpolygyrus=Hpoly_count, Cvitta=Trematode_count, Hymenolepis=Tapeworm_present) %>%
 GGally::ggpairs(columns = 2:5, title = "A) Duodenum counts", upper = "blank", switch = "both")+
 theme(axis.text.x = element_text(angle = 45))
jej_plots <- diss_meta %>% filter(Gut_section=="JEJ") %>%
 select(Animal_number, Syphacia=Syphacia_count, Hpolygyrus=Hpoly_count, Cvitta=Trematode_count, Hymenolepis=Tapeworm_present) %>%
 GGally::ggpairs(columns = 2:5, title = "B) Jejunum counts", upper = "blank", switch = "both")+
 theme(axis.text.x = element_text(angle = 45))
ileum_plots <- diss_meta %>% filter(Gut_section=="ILEUM") %>%
 select(Animal_number, Syphacia=Syphacia_count, Hpolygyrus=Hpoly_count, Cvitta=Trematode_count, Hymenolepis=Tapeworm_present) %>%
 GGally::ggpairs(columns = 2:5, title = "C) Ileum counts", upper = "blank", switch = "both")+
 theme(axis.text.x = element_text(angle = 45))
caecum_plots <- diss_meta %>% filter(Gut_section=="CAECUM") %>%
 select(Animal_number, Syphacia=Syphacia_count, Hpolygyrus=Hpoly_count, Cvitta=Trematode_count, Hymenolepis=Tapeworm_present) %>%
 GGally::ggpairs(columns = 2:5, title = "D) Caecum counts", upper = "blank", switch = "both")+
 theme(axis.text.x = element_text(angle = 45))
colon_plots <- diss_meta %>% filter(Gut_section=="COLON") %>%
 select(Animal_number, Syphacia=Syphacia_count, Hpolygyrus=Hpoly_count, Cvitta=Trematode_count, Hymenolepis=Tapeworm_present) %>%
 GGally::ggpairs(columns = 2:5, title = "E) Colon counts", upper = "blank", switch = "both")+
 theme(axis.text.x = element_text(angle = 45))

# ggsave("Parasite_counts_within_duodenum.jpeg", duo_plots)
# ggsave("Parasite_counts_within_jejunum.jpeg", jej_plots)
# ggsave("Parasite_counts_within_ileum.jpeg", ileum_plots)
# ggsave("Parasite_counts_within_caecum.jpeg", caecum_plots)
# ggsave("Parasite_counts_within_colon.jpeg", colon_plots)


#plot all model vars
covars_plots <- diss_meta %>% filter(!is.na(Tapeworm_present)) %>%
 select(Trap_location, Miseq_run, readDepth,
 Syphacia=Syphacia_count, Hpolygyrus=Hpoly_count,
 Cvitta=Trematode_count, Hymenolepis=Tapeworm_present) %>%
 mutate(Syphacia=log(Syphacia+1), Hpolygyrus=log(Hpolygyrus+1),
 Cvitta=log(Cvitta+1), Hymenolepis=factor(Hymenolepis)) %>%
 GGally::ggpairs( title = "A) Dissection dataset", upper = "blank", switch = "both") +
 theme(axis.text.x = element_text(angle = 45))
#ggsave2("Dissection_covariate_correlations_plot.jpeg", covars_plots)

## Longitudinal dataset summary of parasites found

long_dat3 <- subset(long_dat2, !is.na(Capillaria_EPG))#remove samples with no flotation data

#summary of recaptures per mouse
mouse_sum <- ddply(long_dat3, c("XPIT_tag"), summarise,
 n_obs=length(XPIT_tag)
 )

#within-sample parasite diversity
#hist(long_dat3$parasite_div)
#nrow(subset(long_dat3, parasite_div>0))/nrow(long_dat3)#0.6697
#nrow(subset(long_dat3, parasite_div>1))/nrow(long_dat3)#0.2489
#mean(long_dat3$parasite_div)#1.0181
#sd(long_dat3$parasite_div) / sqrt(length(long_dat3$parasite_div))#0.0644

#population-level prevalence
worm_prevs_long <- long_dat3[,c(52,53,54,55,56,58)] %>% summarise_all(~sum(., na.rm=T)) %>% t() %>% as.data.frame()
worm_prevs_long$Parasite <- rownames(worm_prevs_long)
names(worm_prevs_long)[names(worm_prevs_long)=="V1"] <- "Count"
worm_prevs_long$Parasite <- recode(worm_prevs_long$Parasite, Capillaria_pa="Capillaria sp.", Trichuris_pa="Trichuris sp.",
 Hpoly_pa="H.polygyrus", Syphacia_pa="Syphacia spp.",
 Eimeria_pa="Eimeria spp.", hymenolepid_pa="Hymenolepis sp.")

worm_prevs_long2 <- cbind(worm_prevs_long, as.data.frame(BinomCI(x=worm_prevs_long$Count, n=nrow(long_dat3), method="clopper-pearson")))
worm_prevs_long2$Dataset <- "Longitudinal"

Combine both datasets into one overall prevalence plot

worm_prevs_all <- rbind(worm_prevs_dissection2, worm_prevs_long2)

worm_prevs_all$Parasite <- factor(worm_prevs_all$Parasite,
 levels = c("Capillaria sp.", "Trichuris sp.","H.polygyrus", "Syphacia spp.",
 "Hymenolepis sp.", "C.vitta", "Eimeria spp."))

worm_prevs_plot <- ggplot(worm_prevs_all, aes(x=Parasite, y=est, fill=Dataset), group=Dataset)+
 geom_col(position=position_dodge(preserve = "single")) +
 geom_errorbar(aes(ymin=lwr.ci, ymax=upr.ci), position=position_dodge(preserve = "single"), colour="darkgrey", width=0.5) +
 theme_light() + ylab("Prevalence") +
 scale_fill_brewer(palette = "Set2") + theme(axis.title = element_text(size=15), axis.text = element_text(size=12),
 legend.text = element_text(size=12), legend.title = element_text(size=15))

worm_plots_comb <- grid.arrange(worm_prevs_plot, worm_section_final, nrow=2)
#ggsave2('Fig.1 Worms found both datasets.pdf', worm_plots_comb,width=15,height=10)
#ggsave2('Fig.1 Worms found both datasets.jpeg', worm_plots_comb,width=15,height=10)

proportion of different syphacia species ID’d

#names(dissection_dat)
syphacia_dat <- dissection_dat[,c(34, 37:39)]
syphacia_dat <- subset(syphacia_dat, Syphacia_stored>0)
syphacia_dat$Syphacia_stored_ID_Sstroma[is.na(syphacia_dat$Syphacia_stored_ID_Sstroma)] <- 0
syphacia_dat$Syphacia_stored_ID_Sfrederici[is.na(syphacia_dat$Syphacia_stored_ID_Sfrederici)] <- 0

syphacia_dat$prop_stroma <- syphacia_dat$Syphacia_stored_ID_Sstroma/syphacia_dat$Syphacia_stored
syphacia_dat$prop_fred <- syphacia_dat$Syphacia_stored_ID_Sfrederici/syphacia_dat$Syphacia_stored

syphacia_melt <- melt(syphacia_dat[,c(1,5,6)])
names(syphacia_melt) <- c("Gut_section", "Species", "Proportion")
syphacia_melt$Species <- fct_recode(syphacia_melt$Species, S.stroma="prop_stroma",
 S.frederici="prop_fred")

sample_size <- syphacia_dat %>% group_by(Gut_section) %>%
 dplyr::summarise(N_samp = sum(Syphacia_stored)
 )
sample_size$Proportion <- 0.99

ave_dat <- syphacia_melt %>% group_by(Gut_section, Species) %>%
 dplyr::summarise(ave = mean(Proportion),
 se = sd(Proportion) / sqrt(length(Proportion))
 )

gut_order <- c("DUO", "JEJ", "ILEUM", "CAECUM", "COLON")

syphacia_plot <- ggplot(data=ave_dat, group=Species,
 aes(x=factor(Gut_section, levels = gut_order), y=ave, fill=Species))+
 geom_col(position = "dodge") +
 geom_errorbar(aes(ymax=ave+se, ymin=ave-se, fill=Species), position="dodge",
 colour="darkgrey") +
 theme_light() +
 xlab("Gut section") + ylab("Mean proportion ID'd") +
 geom_text(data=sample_size,
 aes(x=Gut_section,y=Proportion,label=N_samp),
 inherit.aes=FALSE, size=6) +
 theme(axis.title = element_text(size=18), axis.text = element_text(size=16),
 legend.text = element_text(size=16, face = 'italic'),
 legend.title = element_text(size=18))

#ggsave2('FigS1_prop_syphacia_ID_plot.jpeg', syphacia_plot,width=15,height=10)

Correlations among covars

covars_plots_longdat <- long_dat3 %>%
 select(Miseq_run, readDepth,
 Syphacia=Syphacia_EPG, Hpolygyrus=Hpolygyrus_EPG, Aoncotheca=Capillaria_EPG,
 Hymenolepis=Hymenolepid_EPG, Trichuris=Trichuris_EPG, Eimeria=Eimeria_EPG_sum,
 ) %>%
 mutate(Syphacia=log(Syphacia+1), Hpolygyrus=log(Hpolygyrus+1), Aoncotheca=log(Aoncotheca+1),
 Hymenolepis=log(Hymenolepis+1), Trichuris=log(Trichuris+1), Eimeria=log(Eimeria+1),
 Miseq_run=factor(Miseq_run)) %>%
 GGally::ggpairs( title = "B) Longitudinal dataset", upper = "blank", switch = "both")+
 theme(axis.text.x = element_text(angle = 45))
#ggsave2("Longitudinal_covariate_correlations_plot.jpeg", covars_plots_longdat)

#3. Dissection data analyses

##Alpha diversity

General approach; microbiome richness ~ parasite*gut section (term for each parasite). Covars; read depth, miseq run, trap location, sex + random term for ID

#richness estimates using breakaway
ba <- breakaway(dissection_phy.alpha)
#make a dataframe of estimates (same order of samples as in physeq object)
rich_dat <- as.data.frame(summary(ba))
rich_dat2 <- rich_dat[,c(1,2,5)]
colnames(rich_dat2) <- c("richness_estimate", "rich_est_error", "Sequence_sample_code")

#add to main data for modelling
diss_meta <- as(sample_data(dissection_phy.alpha), 'data.frame')
diss_rich <- merge(diss_meta, rich_dat2, by="Sequence_sample_code")
diss_rich <- subset(diss_rich, Gut_section!="POO")
diss_rich$Syphacia_pa <- as.factor(diss_rich$Syphacia_pa)
diss_rich$Hpoly_pa <- as.factor(diss_rich$Hpoly_pa)
diss_rich$Tapeworm_present <- as.factor(diss_rich$Tapeworm_present)
diss_rich$Trematode_pa <- as.factor(diss_rich$Trematode_pa)

#remove missing data
diss_rich2 <- subset(diss_rich, !is.na(richness_estimate) & !is.na(Gut_section) &
 !is.na(Syphacia_pa) & !is.na(Hpoly_pa) & !is.na(Trematode_pa) &
 !is.na(Tapeworm_present) & !is.na(readDepth_z) & !is.na(Miseq_run) &
 !is.na(Trap_location) & !is.na(Animal_number))

#(NOTE; Hpoly and cvitta not found in every gut section therefore can't model these interactions
diss.rich.mod <- brm(richness_estimate ~ Hpoly_pa + Trematode_pa + Gut_section +
 Syphacia_pa*Gut_section + Tapeworm_present*Gut_section +
 readDepth_z + Miseq_run + Trap_location +
 (1|Animal_number),
 data = diss_rich2)

#pp_check(diss.rich.mod)
#plot(diss.rich.mod)
summary(diss.rich.mod)
#conditional_effects(diss.rich.mod)

#assess model fit with LOO-IC
# model.null <- brm(richness_estimate ~ 1+(1|Animal_number),
# data = diss_rich2)
# loo(diss.rich.mod, model.null)#elpd_diff=-58.6


#export results as table
#diss_results_tab <- as.data.frame(fixef(diss.rich.mod, summary=T))
#write.csv(diss_results_tab, 'dissection_richness_model1_tab.csv')

Now look at local/distant effects more closely; one model per gut section with local/distant parasite effects on diversity. Tapeworm presence/absence across whole gut used as couldn’t find any scoloexes, only proglottids

#reshape data into format; one row per animal, one column per parasite/gut section or diversity/ gut section combo, plus covars trap location, read depth, miseq run

diss_rich_melt <- melt(diss_rich[,c(5,7,35,37,41,42,44,74,77,78)], id.vars = c("Animal_number", "Gut_section"))
diss_rich_wide <- dcast(diss_rich_melt, Animal_number ~ Gut_section + variable)

diss_rich_wide <- diss_rich_wide %>%
 mutate_at(c("CAECUM_Syphacia_count","CAECUM_Hpoly_count",
 "CAECUM_Trematode_count", "CAECUM_richness_estimate",
 "CAECUM_readDepth_z" , "COLON_Syphacia_count",
 "COLON_Hpoly_count", "COLON_Trematode_count",
 "COLON_richness_estimate", "COLON_readDepth_z" ,
 "DUO_Syphacia_count","DUO_Hpoly_count" ,
 "DUO_Trematode_count","DUO_richness_estimate",
 "DUO_readDepth_z", "ILEUM_Syphacia_count",
 "ILEUM_Hpoly_count","ILEUM_Trematode_count",
 "ILEUM_richness_estimate","ILEUM_readDepth_z",
 "JEJ_Syphacia_count" , "JEJ_Hpoly_count" ,
 "JEJ_Trematode_count", "JEJ_richness_estimate",
 "JEJ_readDepth_z", "CAECUM_Tapeworm_present",
 "COLON_Tapeworm_present","DUO_Tapeworm_present",
 "ILEUM_Tapeworm_present","JEJ_Tapeworm_present"),
 as.numeric)

Duodenum model

#combine non-duo counts into 'non-local' per parasite
#names(diss_rich_wide)
duo_wide <- diss_rich_wide
duo_wide$Hpoly_nonlocal <- rowSums(duo_wide[,c(5,13,29,37)], na.rm = T)
duo_wide$Hpoly_nonlocal_pa <- ifelse(duo_wide$Hpoly_nonlocal>0, 1, 0)
duo_wide$DUO_Hpoly_pa <- ifelse(duo_wide$DUO_Hpoly>0, 1, 0)

duo_wide$Trem_nonlocal <- rowSums(duo_wide[,c(6,14,30,38)], na.rm = T)
duo_wide$Trem_nonlocal_pa <- ifelse(duo_wide$Trem_nonlocal>0, 1, 0)
duo_wide$DUO_Trematode_pa <- ifelse(duo_wide$DUO_Trematode>0, 1, 0)

duo_wide$Syphacia_nonlocal <- rowSums(duo_wide[,c(3,11,27,35)], na.rm = T)
duo_wide$Syphacia_nonlocal_pa <- ifelse(duo_wide$Syphacia_nonlocal>0, 1, 0)
duo_wide$DUO_Syphacia_pa <- ifelse(duo_wide$DUO_Syphacia>0, 1, 0)

duo_wide$Tapeworm_present <- rowSums(duo_wide[,c(4,12,20,28,36)], na.rm = T)
duo_wide$Tapeworm_present[duo_wide$Tapeworm_present>0] <- 1

#### model - presence/absence all parasites
duo_model <- brm(DUO_richness_estimate ~ factor(DUO_Hpoly_pa) + factor(Hpoly_nonlocal_pa)+
 factor(DUO_Syphacia_pa) + factor(Syphacia_nonlocal_pa) +
 factor(DUO_Trematode_pa) + factor(Trem_nonlocal_pa) +
 factor(Tapeworm_present) + DUO_Trap_location + DUO_readDepth_z +
 DUO_Miseq_run,
 data=duo_wide)

#pp_check(duo_model)
#plot(duo_model)
# model.null <- brm(DUO_richness_estimate ~ 1,
# data=duo_wide)
# loo(duo_model, model.null)#elpd_diff=-2.3

summary(duo_model)#only miseq run
#conditional_effects(duo_model)

#examine fixed effects and plot estimates and CIs
bayes.est.duo <- as.data.frame(fixef(duo_model))
bayes.est.duo$term <- rownames(bayes.est.duo)

#check with abundance of syphacia/hpoly
# duo_model2 <- brm(DUO_richness_estimate ~ log(DUO_Hpoly_count+1) + log(Hpoly_nonlocal+1)+
# log(DUO_Syphacia_count) + log(Syphacia_nonlocal+1) +
# factor(DUO_Trematode_pa) + factor(Trem_nonlocal_pa) +
# factor(Tapeworm_present) + DUO_Trap_location + DUO_readDepth_z +
# DUO_Miseq_run,
# data=duo_wide)
# summary(duo_model2)

Jejenum model

#combine non-duo counts into 'non-local' per parasite
jej_wide <- diss_rich_wide
jej_wide$Hpoly_nonlocal <- rowSums(jej_wide[,c(5,13,29,21)], na.rm = T)
jej_wide$Hpoly_nonlocal_pa <- ifelse(jej_wide$Hpoly_nonlocal>0, 1, 0)
jej_wide$JEJ_Hpoly_pa <- ifelse(jej_wide$JEJ_Hpoly>0, 1, 0)

jej_wide$Trem_nonlocal <- rowSums(jej_wide[,c(6,14,30,22)], na.rm = T)
jej_wide$Trem_nonlocal_pa <- ifelse(jej_wide$Trem_nonlocal>0, 1, 0)
jej_wide$JEJ_Trematode_pa <- ifelse(jej_wide$JEJ_Trematode>0, 1, 0)

jej_wide$Syphacia_nonlocal <- rowSums(jej_wide[,c(3,11,27,19)], na.rm = T)
jej_wide$Syphacia_nonlocal_pa <- ifelse(jej_wide$Syphacia_nonlocal>0, 1, 0)
jej_wide$JEJ_Syphacia_pa <- ifelse(jej_wide$JEJ_Syphacia>0, 1, 0)

jej_wide$Tapeworm_present <- rowSums(jej_wide[,c(4,12,20,28,36)], na.rm = T)
jej_wide$Tapeworm_present[jej_wide$Tapeworm_present>0] <- 1


#model
jej_model <- brm(JEJ_richness_estimate ~ factor(JEJ_Hpoly_pa) + factor(Hpoly_nonlocal_pa)+
 factor(JEJ_Syphacia_pa) + factor(Syphacia_nonlocal_pa) +
 factor(JEJ_Trematode_pa) + factor(Trem_nonlocal_pa) +
 factor(Tapeworm_present) + JEJ_Trap_location + JEJ_readDepth_z +
 JEJ_Miseq_run,
 data=jej_wide)

#pp_check(jej_model)
#plot(jej_model)
#model.null <- brm(JEJ_richness_estimate ~ 1,data=jej_wide)
#loo(jej_model, model.null)#elpd_diff=-7.5

summary(jej_model)
#conditional_effects(jej_model)

#examine fixed effects and plot estimates and CIs
bayes.est.jej <- as.data.frame(fixef(jej_model))
bayes.est.jej$term <- rownames(bayes.est.jej)

#model = abundance of syphacia/hpoly
# jej_model2 <- brm(JEJ_richness_estimate ~ log(JEJ_Hpoly_count+1) + log(Hpoly_nonlocal+1)+
# log(JEJ_Syphacia_count+1) + log(Syphacia_nonlocal+1) +
# factor(JEJ_Trematode_pa) + factor(Trem_nonlocal_pa) +
# factor(Tapeworm_present) + JEJ_Trap_location + JEJ_readDepth_z +
# JEJ_Miseq_run,
# data=jej_wide)
# summary(jej_model2)

Ileum model

#combine non-duo counts into 'non-local' per parasite
ileum_wide <- diss_rich_wide
ileum_wide$Hpoly_nonlocal <- rowSums(ileum_wide[,c(5,13,37,21)], na.rm = T)
ileum_wide$Hpoly_nonlocal_pa <- ifelse(ileum_wide$Hpoly_nonlocal>0, 1, 0)
ileum_wide$ILEUM_Hpoly_pa <- ifelse(ileum_wide$ILEUM_Hpoly>0, 1, 0)

ileum_wide$Trem_nonlocal <- rowSums(ileum_wide[,c(6,14,22,38)], na.rm = T)
ileum_wide$Trem_nonlocal_pa <- ifelse(ileum_wide$Trem_nonlocal>0, 1, 0)
ileum_wide$ILEUM_Trematode_pa <- ifelse(ileum_wide$ILEUM_Trematode>0, 1, 0)

ileum_wide$Syphacia_nonlocal <- rowSums(ileum_wide[,c(3,11,19,35)], na.rm = T)
ileum_wide$Syphacia_nonlocal_pa <- ifelse(ileum_wide$Syphacia_nonlocal>0, 1, 0)
ileum_wide$ILEUM_Syphacia_pa <- ifelse(ileum_wide$ILEUM_Syphacia>0, 1, 0)

ileum_wide$Tapeworm_present <- rowSums(ileum_wide[,c(4,12,20,28,36)], na.rm = T)
ileum_wide$Tapeworm_present[ileum_wide$Tapeworm_present>0] <- 1


#model
#(didn't find hpoly in ileum)
ileum_model <- brm(ILEUM_richness_estimate ~ factor(Hpoly_nonlocal_pa)+
 factor(ILEUM_Syphacia_pa) + factor(Syphacia_nonlocal_pa) +
 factor(ILEUM_Trematode_pa) + factor(Trem_nonlocal_pa) +
 factor(Tapeworm_present) + ILEUM_Trap_location + ILEUM_readDepth_z +
 ILEUM_Miseq_run,
 data=ileum_wide)

#pp_check(ileum_model)
#plot(ileum_model)
# model.null <- brm(ILEUM_richness_estimate ~ 1,
# data=ileum_wide)
# loo(ileum_model, model.null)#elpd_diff=-1.3

summary(ileum_model)
#conditional_effects(ileum_model)

#examine fixed effects and plot estimates and CIs
bayes.est.ileum <- as.data.frame(fixef(ileum_model))
bayes.est.ileum$term <- rownames(bayes.est.ileum)

#check with abundance of syphacia/hpoly
# ileum_model2 <- brm(ILEUM_richness_estimate ~ log(Hpoly_nonlocal+1)+
# log(ILEUM_Syphacia_count+1) + log(Syphacia_nonlocal+1) +
# factor(ILEUM_Trematode_pa) + factor(Trem_nonlocal_pa) +
# factor(Tapeworm_present) + ILEUM_Trap_location + ILEUM_readDepth_z +
# ILEUM_Miseq_run,
# data=ileum_wide)
# summary(ileum_model2)

Caecum model

caecum_wide <- diss_rich_wide
caecum_wide$Hpoly_nonlocal <- rowSums(caecum_wide[,c(13,37,21,29)], na.rm = T)
caecum_wide$Hpoly_nonlocal_pa <- ifelse(caecum_wide$Hpoly_nonlocal>0, 1, 0)
caecum_wide$CAECUM_Hpoly_pa <- ifelse(caecum_wide$CAECUM_Hpoly>0, 1, 0)

caecum_wide$Trem_nonlocal <- rowSums(caecum_wide[,c(14,22,38, 30)], na.rm = T)
caecum_wide$Trem_nonlocal_pa <- ifelse(caecum_wide$Trem_nonlocal>0, 1, 0)
caecum_wide$CAECUM_Trematode_pa <- ifelse(caecum_wide$CAECUM_Trematode>0, 1, 0)

caecum_wide$Syphacia_nonlocal <- rowSums(caecum_wide[,c(11,19,35, 27)], na.rm = T)
caecum_wide$Syphacia_nonlocal_pa <- ifelse(caecum_wide$Syphacia_nonlocal>0, 1, 0)
caecum_wide$CAECUM_Syphacia_pa <- ifelse(ileum_wide$CAECUM_Syphacia>0, 1, 0)

caecum_wide$Tapeworm_present <- rowSums(caecum_wide[,c(4,12,20,28,36)], na.rm = T)
caecum_wide$Tapeworm_present[caecum_wide$Tapeworm_present>0] <- 1


#model
#(didn't find hpoly in caecum#)
caecum_model <- brm(CAECUM_richness_estimate ~ factor(Hpoly_nonlocal_pa)+
 factor(CAECUM_Syphacia_pa) + factor(Syphacia_nonlocal_pa) +
 factor(CAECUM_Trematode_pa) + factor(Trem_nonlocal_pa) +
 factor(Tapeworm_present) + CAECUM_Trap_location + CAECUM_readDepth_z +
 CAECUM_Miseq_run,
 data=caecum_wide)

#pp_check(caecum_model_pa)
#plot(caecum_model)
# model.null <- brm(CAECUM_richness_estimate ~ 1,
# data=caecum_wide)
# loo(caecum_model, model.null)#elpd_diff=-1.3

summary(caecum_model)
#conditional_effects(caecum_model_pa)

#examine fixed effects and plot estimates and CIs
bayes.est.caecum <- as.data.frame(fixef(caecum_model))
bayes.est.caecum$term <- rownames(bayes.est.caecum)

#check with syphacia/hpoly abundance
# caecum_model2 <- brm(CAECUM_richness_estimate ~ log(Hpoly_nonlocal+1)+
# log(CAECUM_Syphacia_count+1) + log(Syphacia_nonlocal+1) +
# factor(CAECUM_Trematode_pa) + factor(Trem_nonlocal_pa) +
# factor(Tapeworm_present) + CAECUM_Trap_location + CAECUM_readDepth_z +
# CAECUM_Miseq_run,
# data=caecum_wide)
# summary(caecum_model2)

Colon model

colon_wide <- diss_rich_wide
colon_wide$Hpoly_nonlocal <- rowSums(colon_wide[,c(5,37,21,29)], na.rm = T)
colon_wide$Hpoly_nonlocal_pa <- ifelse(colon_wide$Hpoly_nonlocal>0, 1, 0)
colon_wide$COLON_Hpoly_pa <- ifelse(colon_wide$COLON_Hpoly>0, 1, 0)

colon_wide$Trem_nonlocal <- rowSums(colon_wide[,c(6,22,38, 30)], na.rm = T)
colon_wide$Trem_nonlocal_pa <- ifelse(colon_wide$Trem_nonlocal>0, 1, 0)
colon_wide$COLON_Trematode_pa <- ifelse(colon_wide$COLON_Trematode>0, 1, 0)

colon_wide$Syphacia_nonlocal <- rowSums(colon_wide[,c(3,19,35, 27)], na.rm = T)
colon_wide$Syphacia_nonlocal_pa <- ifelse(colon_wide$Syphacia_nonlocal>0, 1, 0)
colon_wide$COLON_Syphacia_pa <- ifelse(colon_wide$COLON_Syphacia>0, 1, 0)

colon_wide$Tapeworm_present <- rowSums(colon_wide[,c(4,12,20,28,36)], na.rm = T)
colon_wide$Tapeworm_present[colon_wide$Tapeworm_present>0] <- 1

#model
#(didn't find hpoly or cvitta in colon)
colon_model <- brm(COLON_richness_estimate ~ factor(Hpoly_nonlocal_pa)+
 factor(COLON_Syphacia_pa) + factor(Syphacia_nonlocal_pa) +
 factor(Trem_nonlocal_pa) + factor(Tapeworm_present) +
 COLON_Trap_location + COLON_readDepth_z +
 COLON_Miseq_run,
 data=colon_wide)

#pp_check(colon_model)
#plot(colon_model)
# model.null <- brm(COLON_richness_estimate ~ 1,data=colon_wide)
# loo(colon_model, model.null)#elpd_diff=-3.8
summary(colon_model)
#conditional_effects(colon_model)

#examine fixed effects and plot estimates and CIs
bayes.est.colon <- as.data.frame(fixef(colon_model))
bayes.est.colon $term <- rownames(bayes.est.colon )

#check abundance f syphacia/hpoly
# colon_model2 <- brm(COLON_richness_estimate ~ log(Hpoly_nonlocal+1)+
# log(COLON_Syphacia_count+1) + log(Syphacia_nonlocal+1) +
# factor(Trem_nonlocal_pa) + factor(Tapeworm_present) +
# COLON_Trap_location + COLON_readDepth_z +
# COLON_Miseq_run,
# data=colon_wide)
# summary(colon_model2)

Combine model output plots for all gut sections into one

bayes.est.duo$gut_section <- "Duodenum"
bayes.est.jej$gut_section <- "Jejunum"
bayes.est.ileum$gut_section <- "Ileum"
bayes.est.caecum$gut_section <- "Caecum"
bayes.est.colon$gut_section <- "Colon"

bayes.est.comb <- rbind(bayes.est.duo, bayes.est.jej, bayes.est.ileum,
 bayes.est.caecum, bayes.est.colon)

levels(as.factor(bayes.est.comb$term))
bayes.est.comb$term <- bayes.est.comb$term %>%
 fct_collapse("H.polygyrus (local)" = c( "factorDUO_Hpoly_pa1" , "factorJEJ_Hpoly_pa1" ),
 "H.polgyrus (non-local)"=c("factorHpoly_nonlocal_pa1" ),
 "Syphacia spp. (local)"= c( "factorDUO_Syphacia_pa1", "factorJEJ_Syphacia_pa1",
 "factorILEUM_Syphacia_pa1" ,"factorCAECUM_Syphacia_pa1",
 "factorCOLON_Syphacia_pa1"),
 "Syphacia spp. (non-local)"=c("factorSyphacia_nonlocal_pa1"),
 "C. vitta (local)" = c("factorDUO_Trematode_pa1","factorJEJ_Trematode_pa1" ,
 "factorILEUM_Trematode_pa1" , "factorCAECUM_Trematode_pa1"),
 "C. vitta (non-local)"=c("factorTrem_nonlocal_pa1"),
 "Hymenolepis sp. (whole gut)"= c("factorTapeworm_present1" ),
 "Read depth"= c("DUO_readDepth_z" ,"JEJ_readDepth_z" , "ILEUM_readDepth_z",
 "CAECUM_readDepth_z" ,"COLON_readDepth_z"),
 "MiSeq run"=c("DUO_Miseq_run4", "JEJ_Miseq_run4" , "ILEUM_Miseq_run4" ,
 "CAECUM_Miseq_run4","COLON_Miseq_run4"),
 "Trap location (2)"= c("DUO_Trap_location2" ,"JEJ_Trap_location2" ,
 "ILEUM_Trap_location2","CAECUM_Trap_location2" ,
 "COLON_Trap_location2" ),
 "Trap location (3)"= c("DUO_Trap_location3","JEJ_Trap_location3" ,
 "ILEUM_Trap_location3" ,"CAECUM_Trap_location3" ,
 "COLON_Trap_location3"),
 "Trap location (3 pines)"= c("DUO_Trap_location3Pines" ,"JEJ_Trap_location3Pines" ,
 "ILEUM_Trap_location3Pines" ,"CAECUM_Trap_location3Pines",
 "COLON_Trap_location3Pines"),
 "Trap location (4)"=c("DUO_Trap_location4" ,"JEJ_Trap_location4",
 "ILEUM_Trap_location4","CAECUM_Trap_location4" ,
 "COLON_Trap_location4"),
 "Trap location (main grid)"=c("DUO_Trap_locationMaingrid", "JEJ_Trap_locationMaingrid",
 "ILEUM_Trap_locationMaingrid", "CAECUM_Trap_locationMaingrid",
 "COLON_Trap_locationMaingrid"),
 "Trap location (SW1)"=c("DUO_Trap_locationSW1" , "JEJ_Trap_locationSW1",
 "ILEUM_Trap_locationSW1", "CAECUM_Trap_locationSW1",
 "COLON_Trap_locationSW1"),
 "Trap location (SW3)"=c("DUO_Trap_locationSW3", "JEJ_Trap_locationSW3",
 "ILEUM_Trap_locationSW3", "CAECUM_Trap_locationSW3",
 "COLON_Trap_locationSW3"),
 "Trap location (SW4)"=c("DUO_Trap_locationSW4", "JEJ_Trap_locationSW4",
 "ILEUM_Trap_locationSW4", "CAECUM_Trap_locationSW4",
 "COLON_Trap_locationSW4"))

bayes.est.comb$gut_section <- factor(bayes.est.comb$gut_section, levels = c("Duodenum", "Jejunum", "Ileum", "Caecum", "Colon"))

#change colours of significant terms error bars;
#duo - miseq run
#jej - hymenolepis whole gut
#ileum - c vitta non-local, miseq run
#caecum - c vitta non-local, read depth
#colon - nothing
bayes.est.comb$sig_terms <- "no"
bayes.est.comb$sig_terms[bayes.est.comb$gut_section=="Duodenum" & bayes.est.comb$term=="MiSeq run"] <- "yes"
bayes.est.comb$sig_terms[bayes.est.comb$gut_section=="Jejunum" & bayes.est.comb$term=="Hymenolepis sp. (whole gut)"] <- "yes"
bayes.est.comb$sig_terms[bayes.est.comb$gut_section=="Ileum" & bayes.est.comb$term=="MiSeq run"] <- "yes"
bayes.est.comb$sig_terms[bayes.est.comb$gut_section=="Ileum" & bayes.est.comb$term=="C. vitta (non-local)"] <- "yes"
bayes.est.comb$sig_terms[bayes.est.comb$gut_section=="Caecum" & bayes.est.comb$term=="C. vitta (non-local)"] <- "yes"
bayes.est.comb$sig_terms[bayes.est.comb$gut_section=="Caecum" & bayes.est.comb$term=="Read depth"] <- "yes"

#change order of terms appearing
#levels(as.factor(bayes.est.comb$term))
bayes.est.comb$term <- factor(bayes.est.comb$term, levels = c("Intercept", "MiSeq run",
 "Read depth", "Trap location (2)", "Trap location (3)", "Trap location (3 pines)",
 "Trap location (4)", "Trap location (main grid)", "Trap location (SW1)",
 "Trap location (SW3)", "Trap location (SW4)",
 "C. vitta (local)", "C. vitta (non-local)",
 "H.polygyrus (local)", "H.polgyrus (non-local)", "Syphacia spp. (local)" ,
 "Syphacia spp. (non-local)", "Hymenolepis sp. (whole gut)" ))

rich.plot.comb <- ggplot(subset(bayes.est.comb , term!="Intercept"),
 aes(x=Estimate, y=term, colour=sig_terms)) +
 geom_point() +
 geom_errorbar(aes(xmin=Q2.5, xmax=Q97.5)) +
 scale_colour_manual(values=c("black", "red")) +
 theme_light() +
 theme(axis.title = element_text(size=15), axis.text = element_text(size=12),
 strip.text = element_text(size=15), legend.position = "none") +
 ylab("Term") + xlab("Estimated association with microbial richness") +
 facet_wrap(~gut_section) +
 geom_vline(xintercept = 0, colour="blue")
#ggsave2('FigS3_Bayes.richness.term.estimates.and.CIs.gut.facet.jpeg', rich.plot.comb,width=15,height=10)

check the parasite richness effect on microbiome richness (found below in longitudinal study)

#one row per mouse, use faecal samples and total number of parasites found in whole gut
diss_meta <- as(sample_data(dissection_phy.alpha), 'data.frame')
diss_rich <- merge(diss_meta, rich_dat2, by="Sequence_sample_code")

diss_rich$Tapeworm_present <- as.numeric(diss_rich$Tapeworm_present)

para_div_sum <- diss_rich %>% filter(Gut_section!="POO") %>%
 group_by(Animal_number)%>%
 dplyr::summarise(Syphacia_tot = ifelse(sum(Syphacia_pa)>0,1,0),
 Hpoly_tot = ifelse(sum(Hpoly_pa)>0,1,0),
 Tapeworm_tot = ifelse(sum(Tapeworm_present)>0,1,0),
 Trem_tot = ifelse(sum(Trematode_pa)>0,1,0)) %>%
 mutate(para_div = Syphacia_tot + Hpoly_tot + Tapeworm_tot + Trem_tot)

poo_rich <- diss_rich %>% filter(Gut_section=="POO") %>%
 select(Animal_number, richness_estimate, readDepth_z, Trap_location, Miseq_run)

para_div_dat <- merge(para_div_sum, poo_rich, by="Animal_number")

##model
para_rich_mod <- brm(richness_estimate ~ para_div + Trap_location + Miseq_run + readDepth_z,
 data=para_div_dat)

#pp_check(para_rich_mod)
summary(para_rich_mod)

## Beta diversity

Most abundant bacterial families

#summarise family level abundances
diss_fam_melt <- dissection_phy.beta %>% tax_glom(taxrank = "Family") %>% psmelt()
reads_total <- sum(diss_fam_melt$Abundance)#overall read count=8667053

diss_fam_sum <- ddply(diss_fam_melt, c("Family"), summarise,
 relab = (sum(Abundance)/reads_total)*100
 )
head(arrange(diss_fam_sum, desc(relab)))

#repeat for faecal samples only for comparison to longitudinal dataset
poo_fam_melt <- dissection_phy.beta %>% subset_samples(Gut_section=="POO") %>% tax_glom(taxrank = "Family") %>% psmelt()
reads_total <- sum(poo_fam_melt$Abundance)#overall read count=1387306

poo_fam_sum <- ddply(poo_fam_melt, c("Family"), summarise,
 relab = (sum(Abundance)/reads_total)*100
 )
head(arrange(poo_fam_sum, desc(relab)))

RDA of overall parasite p/a on microbiome composition (with interactions for gut section), plus rda per gut section with local/non-local effects

#convert to relative abundance
dissection_phy.beta.relab <- transform_sample_counts(dissection_phy.beta, function(x) x/sum(x))

#summary(sample_data(dissection_phy.beta.relab))
sample_data(dissection_phy.beta.relab)$Syphacia_pa <- as.factor(sample_data(dissection_phy.beta.relab)$Syphacia_pa)
sample_data(dissection_phy.beta.relab)$Hpoly_pa <- as.factor(sample_data(dissection_phy.beta.relab)$Hpoly_pa)
sample_data(dissection_phy.beta.relab)$Trematode_pa <- as.factor(sample_data(dissection_phy.beta.relab)$Trematode_pa)
sample_data(dissection_phy.beta.relab)$Tapeworm_present <- as.factor(sample_data(dissection_phy.beta.relab)$Tapeworm_present)
sample_data(dissection_phy.beta.relab)$Tapeworm_present[sample_data(dissection_phy.beta.relab)$Tapeworm_present==""] <- NA

dissection_phy.beta.relab <- subset_samples(dissection_phy.beta.relab, Gut_section!="POO")

#overall model with interactions for gut section
#set.seed(12345)
whole_gut_rda <- ordinate(dissection_phy.beta.relab, method = "RDA",
 formula = ~ Syphacia_pa + Hpoly_pa + Trematode_pa + Tapeworm_present + Gut_section +
 Syphacia_pa:Gut_section + Hpoly_pa:Gut_section + Trematode_pa:Gut_section + Tapeworm_present:Gut_section +
 readDepth_z + Trap_location + Miseq_run + Condition(Animal_number),
 na.action=na.fail)

#full model summary
RsquareAdj(whole_gut_rda)
anova(whole_gut_rda)
anova(whole_gut_rda, by="margin")

#model without interactions to assess individual terms
whole_gut_rda2 <- ordinate(dissection_phy.beta.relab, method = "RDA",
 formula = ~ Syphacia_pa + Hpoly_pa + Trematode_pa + Tapeworm_present + Gut_section +
 readDepth_z + Trap_location + Miseq_run + Condition(Animal_number),
 na.action=na.fail)
anova(whole_gut_rda2, by="margin")

Local and non-local effects

Duodenum

# use previous dataframe already formatted for richness models and create phyloseq objects per gut section
#names(duo_wide)
duo_wide <- duo_wide[,c(1,16:24,42:51)]

duo_phy <- subset_samples(dissection_phy.beta.relab, Gut_section=="DUO")
duo_phy_meta <- as(sample_data(duo_phy), 'data.frame')
duo_merge <- merge(duo_phy_meta, duo_wide, by="Animal_number")
rownames(duo_merge) <- duo_merge$Sequence_sample_code

duo_phy <- phyloseq(sample_data(duo_merge), tax_table(tax_table(duo_phy)), otu_table(otu_table(duo_phy)))

#model
duo_rda <- ordinate(duo_phy, method = "RDA",
 formula = ~ factor(DUO_Syphacia_pa) + factor(DUO_Hpoly_pa) + factor(DUO_Trematode_pa) +
 factor(Hpoly_nonlocal_pa) + factor(Syphacia_nonlocal_pa) + factor(Trem_nonlocal_pa) +
 factor(Tapeworm_present.y) +
 readDepth_z + Miseq_run + Trap_location,
 na.action=na.fail)

RsquareAdj(duo_rda)
anova(duo_rda)
duo_tests <- anova(duo_rda, by="margin")

#extract p-values for FDR correction
duo_tests$gut_section <- "DUO"
duo_tests$term <- rownames(duo_tests)

#repeat with syphacia/hpoly abundance
# duo_rda2 <- ordinate(duo_phy, method = "RDA",
# formula = ~ Syphacia_count + Hpoly_count + factor(DUO_Trematode_pa) +
# factor(Tapeworm_present.y) +
# Hpoly_nonlocal + Syphacia_nonlocal + factor(Trem_nonlocal_pa) +
# readDepth_z + Miseq_run + Trap_location,
# na.action=na.fail)
# anova(duo_rda2, by="margin")

Jejenum

#names(jej_wide)
jej_wide <- jej_wide[,c(1,34:39,41,42:51)]

jej_phy <- subset_samples(dissection_phy.beta.relab, Gut_section=="JEJ")
jej_phy_meta <- as(sample_data(jej_phy), 'data.frame')
jej_merge <- merge(jej_phy_meta, jej_wide, by="Animal_number")
rownames(jej_merge) <- jej_merge$Sequence_sample_code
jej_phy <- phyloseq(sample_data(jej_merge), tax_table(tax_table(jej_phy)), otu_table(otu_table(jej_phy)))

#model
jej_rda <- ordinate(jej_phy, method = "RDA",
 formula = ~ factor(JEJ_Syphacia_pa) + factor(JEJ_Hpoly_pa) + factor(JEJ_Trematode_pa) +
 factor(Tapeworm_present.y) +
 factor(Hpoly_nonlocal_pa) + factor(Syphacia_nonlocal_pa) + factor(Trem_nonlocal_pa) +
 readDepth_z + Miseq_run + Trap_location,
 na.action=na.fail)

RsquareAdj(jej_rda)
anova(jej_rda)
jej_tests <- anova(jej_rda, by="margin")

#extract p-values for FDR correction
jej_tests$gut_section <- "JEJ"
jej_tests$term <- rownames(jej_tests)

#repeat with abundance of syphacia/hpoly
# jej_rda2 <- ordinate(jej_phy, method = "RDA",
# formula = ~ Syphacia_count + Hpoly_count + factor(JEJ_Trematode_pa) +
# factor(Tapeworm_present.y) +
# Hpoly_nonlocal + Syphacia_nonlocal + factor(Trem_nonlocal_pa) +
# readDepth_z + Miseq_run + Trap_location,
# na.action=na.fail)
# anova(jej_rda2, by="margin")

Ileum

#names(ileum_wide)
ileum_wide <- ileum_wide[,c(1,26:31,33,42:51)]

ileum_phy <- subset_samples(dissection_phy.beta.relab, Gut_section=="ILEUM")
ileum_phy_meta <- as(sample_data(ileum_phy), 'data.frame')
ileum_merge <- merge(ileum_phy_meta, ileum_wide, by="Animal_number")
rownames(ileum_merge) <- ileum_merge$Sequence_sample_code
ileum_phy <- phyloseq(sample_data(ileum_merge), tax_table(tax_table(ileum_phy)), otu_table(otu_table(ileum_phy)))

#model (don't include local hpoly term)
ileum_rda <- ordinate(ileum_phy, method = "RDA",
 formula = ~ factor(ILEUM_Syphacia_pa) + factor(ILEUM_Trematode_pa) +
 factor(Tapeworm_present.y) +
 factor(Hpoly_nonlocal_pa) + factor(Syphacia_nonlocal_pa) + factor(Trem_nonlocal_pa) +
 readDepth_z + Miseq_run + Trap_location,
 na.action=na.fail)

RsquareAdj(ileum_rda)
anova(ileum_rda)
ileum_tests <- anova(ileum_rda, by="margin")

#extract p-values for FDR correction
ileum_tests$gut_section <- "ILEUM"
ileum_tests$term <- rownames(ileum_tests)

#check syphacia/hply abundance
# ileum_rda2 <- ordinate(ileum_phy, method = "RDA",
# formula = ~ Syphacia_count + factor(ILEUM_Trematode_pa) +
# factor(Tapeworm_present.y) + +
# Hpoly_nonlocal + Syphacia_nonlocal + factor(Trem_nonlocal_pa) +
# readDepth_z + Miseq_run + Trap_location,
# na.action=na.fail)
# anova(ileum_rda2, by="margin")

Caecum

#names(caecum_wide)
caecum_wide <- caecum_wide[,c(1:7,42:51)]

caecum_phy <- subset_samples(dissection_phy.beta.relab, Gut_section=="CAECUM")
caecum_phy_meta <- as(sample_data(caecum_phy), 'data.frame')
caecum_merge <- merge(caecum_phy_meta, caecum_wide, by="Animal_number")
rownames(caecum_merge) <- caecum_merge$Sequence_sample_code
caecum_phy <- phyloseq(sample_data(caecum_merge), tax_table(tax_table(caecum_phy)), otu_table(otu_table(caecum_phy)))

#model (don't include local hpoly term)
caecum_rda <- ordinate(caecum_phy, method = "RDA",
 formula = ~ factor(CAECUM_Syphacia_pa) + factor(CAECUM_Trematode_pa) +
 factor(Tapeworm_present.y) +
 factor(Hpoly_nonlocal_pa) + factor(Syphacia_nonlocal_pa) + factor(Trem_nonlocal_pa) +
 readDepth_z + Miseq_run + Trap_location,
 na.action=na.fail)

RsquareAdj(caecum_rda)
anova(caecum_rda)
caecum_tests <- anova(caecum_rda, by="margin")

#extract p-values for FDR correction
caecum_tests$gut_section <- "CAECUM"
caecum_tests$term <- rownames(caecum_tests)

#check syphacia/hply abundance
# caecum_rda2 <- ordinate(caecum_phy, method = "RDA",
# formula = ~ Syphacia_count + factor(CAECUM_Trematode_pa) +
# factor(Tapeworm_present.y) + +
# Hpoly_nonlocal + Syphacia_nonlocal + factor(Trem_nonlocal_pa) +
# readDepth_z + Miseq_run + Trap_location,
# na.action=na.fail)
# anova(caecum_rda2, by="margin")

Colon

#names(colon_wide)
colon_wide <- colon_wide[,c(1,10:15,42:51)]

colon_phy <- subset_samples(dissection_phy.beta.relab, Gut_section=="COLON")
colon_phy_meta <- as(sample_data(colon_phy), 'data.frame')
colon_merge <- merge(colon_phy_meta, colon_wide, by="Animal_number")
rownames(colon_merge) <- colon_merge$Sequence_sample_code
colon_phy <- phyloseq(sample_data(colon_merge), tax_table(tax_table(colon_phy)), otu_table(otu_table(colon_phy)))

#model (don't include local hpoly OR CVITTA terms)
colon_rda <- ordinate(colon_phy, method = "RDA",
 formula = ~ factor(COLON_Syphacia_pa) + factor(Tapeworm_present.y) +
 factor(Hpoly_nonlocal_pa) + factor(Syphacia_nonlocal_pa) + factor(Trem_nonlocal_pa) +
 readDepth_z + Miseq_run + Trap_location,
 na.action=na.fail)

RsquareAdj(colon_rda)
anova(colon_rda)
colon_tests <- anova(colon_rda, by="margin")

#extract p-values for FDR correction
colon_tests$gut_section <- "COLON"
colon_tests$term <- rownames(colon_tests)

#Check syphacia/hpoly abundance
# colon_rda2 <- ordinate(colon_phy, method = "RDA",
# formula = ~ Syphacia_count + factor(Tapeworm_present.y) +
# Hpoly_nonlocal + Syphacia_nonlocal + factor(Trem_nonlocal_pa) +
# readDepth_z + Miseq_run + Trap_location,
# na.action=na.fail)
# anova(colon_rda2, by="margin")

Correct for multiple testing

tests_comb <- rbind(duo_tests, jej_tests, ileum_tests, caecum_tests, colon_tests)
tests_comb$term <- tests_comb$term %>%
 fct_collapse("Syphacia_local"= c("factor(DUO_Syphacia_pa)", "factor(JEJ_Syphacia_pa)",
 "factor(ILEUM_Syphacia_pa)", "factor(CAECUM_Syphacia_pa)",
 "factor(COLON_Syphacia_pa)"),
 "Hpoly_local" = c("factor(DUO_Hpoly_pa)", "factor(JEJ_Hpoly_pa)"),
 "Trematode_local" = c("factor(DUO_Trematode_pa)", "factor(JEJ_Trematode_pa)",
 "factor(ILEUM_Trematode_pa)", "factor(CAECUM_Trematode_pa)"))
levels(tests_comb$term)
p.adjust(subset(tests_comb, term=="Syphacia_local")$'Pr(>F)')
p.adjust(subset(tests_comb, term=="factor(Syphacia_nonlocal_pa)")$'Pr(>F)')
p.adjust(subset(tests_comb, term=="Hpoly_local")$'Pr(>F)')
p.adjust(subset(tests_comb, term=="factor(Hpoly_nonlocal_pa)")$'Pr(>F)')
p.adjust(subset(tests_comb, term=="factor(Tapeworm_present.y)")$'Pr(>F)')
p.adjust(subset(tests_comb, term=="Trematode_local")$'Pr(>F)')
p.adjust(subset(tests_comb, term=="factor(Trem_nonlocal_pa)")$'Pr(>F)')
p.adjust(subset(tests_comb, term=="readDepth_z")$'Pr(>F)')
p.adjust(subset(tests_comb, term=="Miseq_run")$'Pr(>F)')
p.adjust(subset(tests_comb, term=="Trap_location")$'Pr(>F)')

#4. Longitudinal data

##Alpha diversity

Microbiome richness ~ Overall parasite diversity

ba <- breakaway(long_phy.alpha)
rich_dat <- as.data.frame(summary(ba))
rich_dat2 <- rich_dat[,c(1,2,5)]
colnames(rich_dat2) <- c("richness_estimate", "rich_est_error", "Sequence_sample_code")

long_phy_merge <- merge(long_dat3, rich_dat2, by="Sequence_sample_code")

#basic stats on micrbiome data for this subset
# head(long_phy_merge)
#length(levels(long_phy_merge$XPIT_tag))#218
# mean(long_phy_merge$readDepth)
# sd(long_phy_merge$readDepth)/sqrt(nrow(long_phy_merge))
#mean(long_phy_merge$richness_estimate)
#sd(long_phy_merge$richness_estimate)/sqrt(nrow(long_phy_merge))
# range(long_phy_merge$richness_estimate)

#group multiple infections together; no infection - single infection - multiple infection
long_phy_merge$parasite_div[long_phy_merge$parasite_div>=2] <- 2

#model
model_div <- brm(richness_estimate ~ parasite_div + readDepth_z +
 Miseq_run + (1|XPIT_tag),
 data=long_phy_merge,
 family=gaussian(),
 iter = 5000)

#pp_check(model_div)
summary(model_div)
#plot(model_div)
#conditional_effects(model_div)
#bayes_R2(model_div)

#assess model fit using loo-ic
# model_div_null <- brm(richness_estimate ~ 1 + (1|XPIT_tag),
# data=long_phy_merge,
# family=gaussian())
# loo(model_div, model_div_null)#elpd_diff=-14.7

#get conditional effect plot output for parasite_div term and save as a ggplot
para_divv_effects <- conditional_effects(model_div, effects = "parasite_div")
#para_divv_effects$parasite_div

para_div_plot <- ggplot(para_divv_effects$parasite_div,
 aes(x=parasite_div, y=estimate__)) +
 geom_line(linewidth=1.5) +
 geom_ribbon(aes(ymin=lower__, ymax=upper__), alpha=0.35) +
 theme_light() +
 theme(axis.title = element_text(size=18),
 axis.text =element_text(size=16)) +
 xlab("Parasite diversity") +
 ylab("Microbial richness") +
 geom_jitter(data=long_phy_merge, aes(x=parasite_div, y=richness_estimate), alpha=0.5, width=0.2) +
 scale_x_continuous(breaks= c(0,1,2), labels = c("0", "1", "2>="))

#ggsave2('Fig.2 Microbiome and parasite diversity plot.pdf', para_div_plot)
#ggsave2('Fig.2 Microbiome and parasite diversity plot.jpeg', para_div_plot, width=12, height=8)

Microbiome richness ~ Each parasite presence

long_phy_merge$Capillaria_pa <- as.factor(long_phy_merge$Capillaria_pa)
long_phy_merge$Trichuris_pa <- as.factor(long_phy_merge$Trichuris_pa)
long_phy_merge$Hpoly_pa <- as.factor(long_phy_merge$Hpoly_pa)
long_phy_merge$Syphacia_pa <- as.factor(long_phy_merge$Syphacia_pa)
long_phy_merge$Eimeria_pa <- as.factor(long_phy_merge$Eimeria_pa)
long_phy_merge$hymenolepid_pa <- as.factor(long_phy_merge$hymenolepid_pa)

#model - presencce/absence of each parasite
model_div2 <- brm(richness_estimate ~ Capillaria_pa + Trichuris_pa +
 Hpoly_pa + Syphacia_pa + Eimeria_pa + hymenolepid_pa +
 readDepth_z + Miseq_run + (1|XPIT_tag),
 data=long_phy_merge)

#pp_check(model_div2)
summary(model_div2)

#plot model estimates
bayes.est.pa <- as.data.frame(fixef(model_div2))
bayes.est.pa$term <- rownames(bayes.est.pa)
bayes.est.pa$model <- "presence/absence"

Microbiome richness ~ Each parasite burden

#rescale read depth and EPGs to avoid scaling issues
long_phy_merge$Capillaria_EPG_z <- (long_phy_merge$Capillaria_EPG - mean(long_phy_merge$Capillaria_EPG))/sd(long_phy_merge$Capillaria_EPG)
long_phy_merge$Trichuris_EPG_z <- (long_phy_merge$Trichuris_EPG - mean(long_phy_merge$Trichuris_EPG))/sd(long_phy_merge$Trichuris_EPG)
long_phy_merge$Hpolygyrus_EPG_z <- (long_phy_merge$Hpolygyrus_EPG - mean(long_phy_merge$Hpolygyrus_EPG))/sd(long_phy_merge$Hpolygyrus_EPG)
long_phy_merge$Syphacia_EPG_z <- (long_phy_merge$Syphacia_EPG - mean(long_phy_merge$Syphacia_EPG))/sd(long_phy_merge$Syphacia_EPG)
long_phy_merge$Eimeria_EPG_z <- (long_phy_merge$Eimeria_EPG_sum - mean(long_phy_merge$Eimeria_EPG_sum))/sd(long_phy_merge$Eimeria_EPG_sum)
long_phy_merge$Hymenolepid_EPG_z <- (long_phy_merge$Hymenolepid_EPG - mean(long_phy_merge$Hymenolepid_EPG))/sd(long_phy_merge$Hymenolepid_EPG)


model_div3 <- brm(richness_estimate ~ Capillaria_EPG_z + Trichuris_EPG_z +
 Hpolygyrus_EPG_z +
 Syphacia_EPG_z + Eimeria_EPG_z + Hymenolepid_EPG_z +
 readDepth_z + Miseq_run + (1|XPIT_tag),
 data=long_phy_merge)

#pp_check(model_div3)
summary(model_div3) #read depth and trichuris seem important

#plot model estimates
bayes.est.abund <- as.data.frame(fixef(model_div3))
bayes.est.abund$term <- rownames(bayes.est.abund)
bayes.est.abund$model <- "abundance"

#plot both p/a and abundance results together
bayes.est.comb2 <- rbind(bayes.est.pa, bayes.est.abund)

bayes.est.comb2$term <- bayes.est.comb2$term %>%
 fct_collapse("Aoncotheca sp."=c("Capillaria_EPG_z", "Capillaria_pa1"),
 "Eimeria spp."=c("Eimeria_EPG_z", "Eimeria_pa1"),
 "H. polygyrus"=c("Hpoly_pa1", "Hpolygyrus_EPG_z"),
 "Hymenolepis sp."=c("Hymenolepid_EPG_z", "hymenolepid_pa1"),
 "Syphacia spp."=c("Syphacia_EPG_z", "Syphacia_pa"),
 "Trichuris sp."=c("Trichuris_EPG_z", "Trichuris_pa1"),
 "Read depth"="readDepth_z",
 "MiSeq run"="Miseq_run")

bayes.est.comb2$term <- factor(bayes.est.comb2$term, levels = c("Read depth", "MiSeq run", "Aoncotheca sp.",
 "Eimeria spp.", "H. polygyrus", "Hymenolepis sp.",
 "Syphacia spp.","Trichuris sp."))

facet_labels <- list("abundance"="Parasite egg/oocyst burden (EPG z-score)", "presence/absence"="Parasite presence/ absence")
facet_labeller <- function(variable,value){
 return(facet_labels[value])
}


bayes.richness.plot <- ggplot(subset(bayes.est.comb2, term!="Intercept"),
 aes(x=Estimate, y=term)) +
 geom_point(size=3) +
 geom_errorbar(aes(xmin=Q2.5, xmax=Q97.5, width=0.4)) +
 theme_light() +
 theme(axis.title = element_text(size=16), axis.text = element_text(size=14),
 strip.text = element_text(size=16)) +
 ylab("Term") + xlab("Estimated effect on microbial richness") +
 facet_grid(~model, labeller = facet_labeller) +
 geom_vline(xintercept = 0, colour="blue")

#ggsave2('bayes effects parasite microbial richness longitudinal.jpeg', bayes.richness.plot,width=15,height=10)

##Beta diversity

Summarise family-level abundances and compare to dissection poo samples

#summarise family level abundances
poo_fam_melt2 <- long_phy.beta %>% subset_samples(!is.na(Capillaria_EPG)) %>% tax_glom(taxrank = "Family") %>% psmelt()
reads_total <- sum(poo_fam_melt2$Abundance)#overall read count=8766058

poo_fam_sum2 <- ddply(poo_fam_melt2, c("Family"), summarise,
 relab = (sum(Abundance)/reads_total)*100
 )

arrange(poo_fam_sum, desc(relab))
arrange(poo_fam_sum2, desc(relab))

Microbiome composition ~ Parasite composition (Mantel)

#long_phy.beta#keep only samples with corresponding parasite data
long_phy_meta <- as(sample_data(long_phy.beta), 'data.frame')
long_phy_meta <- subset(long_phy_meta, !is.na(Capillaria_EPG))
#names(long_phy_meta)
parasite_matrix <- long_phy_meta[,c(32,33,35,40,46,65)]
empty_samples <- as.list(rownames(parasite_matrix)[rowSums(parasite_matrix)==0])#samples with 0 parasites detected
all_samples <- as.list(rownames(parasite_matrix))
keep_samples <- setdiff(all_samples,empty_samples)
parasite_matrix <- parasite_matrix[rownames(parasite_matrix)%in%keep_samples,]
parasite_matrix <- as.matrix(parasite_matrix)
D_para <- vegdist(parasite_matrix, method="bray")

long_phy.beta.parasub <- subset_samples(long_phy.beta, Sequence_sample_code%in%keep_samples)
D_mb <- distance(long_phy.beta.parasub, method="bray", type="samples")

#test
mantel(D_mb, D_para, method="pearson", permutations = 999)

Microbiome composition ~ Each parasite presence (pRDA)

#set.seed(12345)

rownames(long_phy_merge) <- long_phy_merge$Sequence_sample_code
long_phy_merge <- subset(long_phy_merge, !is.na(XPIT_tag))
long_phy.beta.parasub <- phyloseq(sample_data(long_phy_merge), tax_table(tax_table(long_phy.beta)), otu_table(otu_table(long_phy.beta)))
long_phy.beta.parasub <- transform(long_phy.beta.parasub, transform = "hellinger", target="OTU")

paraRDA <- ordinate(long_phy.beta.parasub, method="RDA", formula = ~ Capillaria_EPG_z + Trichuris_EPG_z + Hpolygyrus_EPG_z +
 Syphacia_EPG_z + Eimeria_EPG_z + Hymenolepid_EPG_z + Miseq_run + readDepth_z + Condition(XPIT_tag))


#RsquareAdj(paraRDA)
#anova(paraRDA)
anova(paraRDA, by="margin")

microbiome PC1 seasonality ~ Each parasite presence/abundance (GAMM)

#get PC1 scores from ordination of full wytham microbiome dataset
long_phy.beta.relab <- transform_sample_counts(long_phy.beta, function(x) x/sum(x))
sample_ord_bray_wyt <- ordinate(long_phy.beta.relab, method= "PCoA", distance= 'bray')
##extract first few axes as variables in metadata
axis_sub_wyt <- sample_ord_bray_wyt$vectors[,1:6]
axis_sub_wyt <- cbind(rownames(axis_sub_wyt), axis_sub_wyt)
colnames(axis_sub_wyt)[1] <- "Sequence_sample_code"
axis_sub_wyt <- as.data.frame(axis_sub_wyt)
physeq_met_wyt <- as(sample_data(long_phy.beta), "data.frame")
physeq_merge <- merge(physeq_met_wyt, axis_sub_wyt, by="Sequence_sample_code")
#summary(physeq_merge)
physeq_merge$Axis.1 <- as.numeric(physeq_merge$Axis.1)
physeq_merge$Year <- lubridate::year(physeq_merge$Collection_date)
physeq_merge$Year <- as.factor(physeq_merge$Year)
physeq_merge <- subset(physeq_merge, !is.na(XPIT_tag))

### PC1 GAMM (whole dataset)
library(mgcv)
model1 <- gamm(Axis.1 ~ s(Day_of_year, bs = "cc", k=24) + Year,
 data = physeq_merge,
 random=list(XPIT_tag=~1),
 na.action = na.exclude)
#plot(model1$gam)
#looks good

##plot of seasonal pc1 with gamm and raw points (highlighting subset of data used for parasite-microbiome analyses in different colour)
pdat <- expand.grid(Day_of_year=seq(0,365,14), Year="2017")
p <- predict(model1$gam, newdata = pdat, type = "response", se.fit = TRUE, level=1)
pdat$p <- p$fit
pdat$se <- p$se.fit

names(pdat)[names(pdat)=="p"] <- "Axis.1"
physeq_merge$Flotations_done <- ifelse(!is.na(physeq_merge$Capillaria_EPG), "Y", "N")

p1 <- ggplot(pdat, aes(x = Day_of_year, y = -Axis.1)) +
 geom_line(size=1) +
 geom_ribbon(aes(ymin=-Axis.1-se, ymax=-Axis.1+se), alpha=0.15) +
 geom_point(data=physeq_merge, alpha=0.3, aes(colour=Flotations_done)) +
 theme_bw() +
 ylab("PC1 (12.89% variance)") + xlab("Collection date") +
 theme(axis.text=element_text(size=13), axis.title=element_text(size=14)) +
 scale_fill_discrete(name = "Parasite data subset")+
 scale_x_continuous(breaks=cumsum(c(1,31,28,31,30,31,30,31,31,30,31,30,30)),
 labels =c("Jan","Feb","Mar","Apr","May","Jun","Jul","Aug",
 "Sep","Oct","Nov","Dec", "Jan"),
 expand = c(0,0))
p1

#ggsave("Microbiota_PC1_seasonal_gamm_higlighted_parasite_subset.jpeg", p1, width = 10, height = 6)

plot of seasonal patterns in parasite loads

physeq_merge_sub <- subset(physeq_merge, !is.na(Capillaria_EPG))

parasite_season_dat <- physeq_merge_sub %>%
 select(Day_of_year, Aoncotheca=Capillaria_EPG, Trichuris=Trichuris_EPG,
 Hpolygyrus=Hpolygyrus_EPG, Syphacia=Syphacia_EPG, Eimeria=Eimeria_EPG_sum,
 Hymenolepid=Hymenolepid_EPG) %>%
 pivot_longer(!Day_of_year, names_to = "Parasite", values_to = "EPG")

parasite_season_plot <- ggplot(parasite_season_dat, aes(x=Day_of_year, y=log(EPG+1))) +
 geom_point(alpha=0.5) + geom_smooth(method = "gam", formula = y ~ s(x, bs = "cc")) +
 facet_wrap(~Parasite, scales = "free_y") +
 theme_bw() +
 xlab("Day of the year")

#ggsave("Seasonality_parasite_EPG_facetted.jpeg", parasite_season_plot, width=10, height=7)

Add parasite terms to microbiota PC1 gamm

#rescale read depth and EPGs to avoid scaling issues
physeq_merge_sub$Capillaria_EPG_z <- (physeq_merge_sub$Capillaria_EPG - mean(physeq_merge_sub$Capillaria_EPG))/sd(physeq_merge_sub$Capillaria_EPG)
physeq_merge_sub$Trichuris_EPG_z <- (physeq_merge_sub$Trichuris_EPG - mean(physeq_merge_sub$Trichuris_EPG))/sd(physeq_merge_sub$Trichuris_EPG)
physeq_merge_sub$Hpolygyrus_EPG_z <- (physeq_merge_sub$Hpolygyrus_EPG - mean(physeq_merge_sub$Hpolygyrus_EPG))/sd(physeq_merge_sub$Hpolygyrus_EPG)
physeq_merge_sub$Syphacia_EPG_z <- (physeq_merge_sub$Syphacia_EPG - mean(physeq_merge_sub$Syphacia_EPG))/sd(physeq_merge_sub$Syphacia_EPG)
physeq_merge_sub$Eimeria_EPG_z <- (physeq_merge_sub$Eimeria_EPG_sum - mean(physeq_merge_sub$Eimeria_EPG_sum))/sd(physeq_merge_sub$Eimeria_EPG_sum)
physeq_merge_sub$Hymenolepid_EPG_z <- (physeq_merge_sub$Hymenolepid_EPG - mean(physeq_merge_sub$Hymenolepid_EPG))/sd(physeq_merge_sub$Hymenolepid_EPG)

#with parasite terms
model2 <- gamm(Axis.1 ~ s(Day_of_year, bs = "cc") +
 Year + Miseq_run + readDepth_z +
 Capillaria_EPG_z + Trichuris_EPG_z + Hpolygyrus_EPG_z +
 Syphacia_EPG_z + Eimeria_EPG_z + Hymenolepid_EPG_z,
 data = physeq_merge_sub,
 random=list(XPIT_tag=~1),
 na.action = na.exclude)

#plot(model2$gam, scale = 0)
summary(model2$gam)
#Rsq adj=0.485 with parasite terms
#anova(model2$lme)
anova(model2$gam)

#wihtout parasite terms
#with parasite terms
model2b <- gamm(Axis.1 ~ s(Day_of_year, bs = "cc") +
 Year + Miseq_run + readDepth_z ,
 data = physeq_merge_sub,
 random=list(XPIT_tag=~1),
 na.action = na.exclude)
summary(model2b$lme)
#plot(model2b$gam, scale = 0)
summary(model2b$gam)
#Rsq adj=0.462 without parasite terms
#anova(model2$lme)
#anova(model2$gam)
#AIC(model2, model2b)

#now run a gamm for trichuris and eimeria to see what seasonal patterns they show

model3 <- gamm(Trichuris_EPG_z ~ s(Day_of_year, bs = "cc", k=24) +Year,
 data = physeq_merge_sub,
 random=list(XPIT_tag=~1),
 na.action = na.exclude)
#plot(model3$gam)
summary(model3$gam)

model4 <- gamm(Eimeria_EPG_z ~ s(Day_of_year, bs = "cc", k=24) +Year,
 data = physeq_merge_sub,
 random=list(XPIT_tag=~1),
 na.action = na.exclude)
#plot(model4$gam)
summary(model4$gam)

#5. Supplementary faeces vs. gut section analysis

alpha diversity

#richness estimates using breakaway
ba <- breakaway(dissection_phy.alpha)
rich_dat <- as.data.frame(summary(ba))
rich_dat2 <- rich_dat[,c(1,2,5)]
colnames(rich_dat2) <- c("richness_estimate", "rich_est_error", "Sequence_sample_code")

#add to main data for modelling
diss_meta <- as(sample_data(dissection_phy.alpha), 'data.frame')
diss_rich <- merge(diss_meta, rich_dat2, by="Sequence_sample_code")
diss_rich$Gut_section <- relevel(diss_rich$Gut_section, ref = "POO")#make sure poo is reference category so all gut sections are compared to it in the model

poo.model1 <- brm(richness_estimate ~ Gut_section + readDepth_z + Miseq_run + Trap_location + (1|Animal_number),
 data = diss_rich)

#pp_check(poo.model1)
#plot(poo.model1)
summary(poo.model1)

#assess model fit with LOO-IC
# model.null <- brm(richness_estimate ~ 1+(1|Animal_number),
# data = diss_rich)
# loo(poo.model1, model.null)#elpd_diff=-77.1

#plot conditional effects of gut section
gut_effects <- conditional_effects(poo.model1, effects = "Gut_section")
gut_effects <- as.data.frame(gut_effects$Gut_section)
gut_effects$Gut_section<- factor(gut_effects$Gut_section, levels = c("DUO", "JEJ", "ILEUM", "CAECUM", "COLON", "POO"))

level_order_gut <-c("DUO", "JEJ", "ILEUM", "CAECUM", "COLON", "POO")

gut_rich_plot3 <- ggplot(gut_effects,aes(x=factor(Gut_section, levels = level_order_gut), y=estimate__)) +
 geom_violin(data=diss_rich, aes(x=factor(Gut_section, levels = level_order_gut), y=richness_estimate), alpha=0.4) +
 geom_point(data=diss_rich, aes(x=factor(Gut_section, levels = level_order_gut), y=richness_estimate), alpha=0.4) +
 geom_errorbar(aes(ymin=lower__, ymax=upper__), alpha=0.4, width=0.3) +
 geom_point(size=6, shape=21, fill="white") +
 theme_light() +
 theme(axis.title = element_text(size=20),
 axis.text =element_text(size=16)) +
 xlab("Sample type") +
 ylab("Microbial richness") +
 scale_x_discrete(labels = c("DUO", "JEJ", "ILEUM", "CAECUM", "COLON", "FAECES")) +
 theme(axis.title = element_text(size=15), axis.text = element_text(size=12),
 strip.text = element_text(size=15),
 axis.text.x = element_blank(), axis.title.x = element_blank())

#plot estimates and CIs

#examine fixed effects and plot estimates and CIs
bayes.est <- as.data.frame(fixef(poo.model1))
bayes.est$term <- rownames(bayes.est)

bayes.est$term <- recode(bayes.est$term , Gut_sectionCAECUM="Caecum",
 Gut_sectionCOLON="Colon",
 Gut_sectionDUO="Duodenum",
 Gut_sectionILEUM="Ileum",
 Gut_sectionJEJ="Jejenum",
 readDepth_z="read depth",
 Miseq_run2="Miseq run (2)",
 Trap_location2="Trap location (2)",
 Trap_location3="Trap location (3)",
 Trap_location3Pines="Trap location (3Pines)",
 Trap_location4="Trap location (4)",
 Trap_locationMaingrid="Trap location (Main grid)",
 Trap_locationSW1="Trap location (SW1)",
 Trap_locationSW3="Trap location (SW3)",
 Trap_locationSW4="Trap location (SW4)")

gut.rich.plot4 <- ggplot(subset(bayes.est, term!="Intercept"),
 aes(x=Estimate, y=term)) +
 geom_point(size=2) +
 geom_errorbar(aes(xmin=Q2.5, xmax=Q97.5, width=0.5)) +
 theme_light() +
 theme(axis.title = element_text(size=15), axis.text = element_text(size=12)) +
 ylab("Term") + xlab("Estimated association with microbial richness") +
 geom_vline(xintercept = 0, colour="blue")

beta diversity

Stacked barplot of composition per gut section (+poo)

#What Are the Names of the most abundant phyla?
 diss_famcollapse <- dissection_phy.beta %>% aggregate_taxa(level="Family")
 diss_top6fam = names(sort(taxa_sums(diss_famcollapse), TRUE)[1:6])

 #melt phyloseq to long format for plotting
diss_famGlom <- dissection_phy.beta %>%
 microbiome::transform(transform = "compositional") %>%
 tax_glom(taxrank = "Family") %>%
 psmelt() %>%
 arrange(Family)

#names(diss_famGlom)
all_tax_list <- as.list(unique(as.character(diss_famGlom$Family)))
diff_list <-as.character(setdiff(all_tax_list, diss_top6fam))
diss_famGlom$Family <- diss_famGlom$Family %>% fct_collapse(Other = diff_list)
diss_famGlom$Family <- fct_recode(diss_famGlom$Family, Muribaculaceae="Bacteroidales_S24-7_group")
diss_famGlom$Gut_section <- fct_recode(diss_famGlom$Gut_section, FAECES="POO")

level_order_gut <- c("DUO", "JEJ", "ILEUM", "CAECUM", "COLON", "FAECES")

gut_comp_plot <- ggplot(diss_famGlom, aes(x = factor(Gut_section, levels = level_order_gut), y = Abundance, fill = Family)) +
 geom_bar(stat = "identity", position="fill") +
 ylab("Relative Abundance") + xlab("Gut section") +
 theme_light() +
 theme(axis.title = element_text(size=15), axis.text = element_text(size=12),
 legend.text = element_text(size=10), legend.title = element_text(size=12), strip.text = element_text(size=12)) +
 scale_fill_brewer(palette = "Pastel2")

#combine with richness plot per gut section for main text figure
library(ggpubr)
poo.gut.plotcomb <- ggarrange(gut_rich_plot3,gut_comp_plot,
 labels = c("A)", "B)"),
 ncol = 1, nrow = 2,
 common.legend = TRUE, legend = "right")

#ggsave2('Fig2_faecal_and_gut_sample_comparisons.jpeg', poo.gut.plotcomb)

PERMANOVA

dissection_phy.beta.relab <- transform_sample_counts(dissection_phy.beta, function(x) x/sum(x))
D_mb <- distance(dissection_phy.beta.relab, method="bray", type="samples")
M <- as(sample_data(dissection_phy.beta.relab), 'data.frame')

#set.seed(12345)
poo.perm <- adonis2(D_mb ~ Gut_section + Trap_location +
 Miseq_run + readDepth_z, strata = M$Animal_number,
 data=M,
 by="margin")

#test for differences in group dispersions
beta <- betadisper(D_mb, M$Gut_section)
permutest(beta)

PERMANOVA - poo v colon only (compare variance explained by sample type/ mouse ID)

colon_poo_phy <- subset_samples(dissection_phy.beta.relab, Gut_section%in%c("COLON", "POO"))

D_mb <- distance(colon_poo_phy, method="bray", type="samples")
M <- as(sample_data(colon_poo_phy), 'data.frame')

#set.seed(123456)
poo.perm2 <- adonis2(D_mb ~ Gut_section + Animal_number + readDepth_z,
 data=M,
 by="margin")

#test for differences in group dispersions
beta <- betadisper(D_mb, M$Gut_section)
permutest(beta)
boxplot(beta)

beta <- betadisper(D_mb, M$Animal_number)
permutest(beta)

Procrustes analysis - colon v poo

#prepare pca's of colon and poo samples (check animal ID is the naming vairable)

#check which animals have both colon and poos sampels matched
animal_list <- rownames(subset(as.data.frame(summary(sample_data(colon_poo_phy)$Animal_number)), summary(sample_data(colon_poo_phy)$Animal_number)==2))
#change orientation of out table
colon_poo_phy <- phyloseq(sample_data(sample_data(colon_poo_phy)), tax_table(tax_table(colon_poo_phy)),
 otu_table(t(otu_table(colon_poo_phy))))
#taxa_are_rows(colon_poo_phy)

poo_phy <- subset_samples(colon_poo_phy, Gut_section%in%c("POO")& Animal_number%in%animal_list)
sample_names(poo_phy) <- sample_data(poo_phy)$Animal_number
poo_phy <- prune_taxa(taxa_sums(poo_phy) > 0, poo_phy)
poo_ord <- ordinate(poo_phy, method= "RDA", distance= "bray", type="samples")

colon_phy <- subset_samples(colon_poo_phy, Gut_section%in%c("COLON") & Animal_number%in%animal_list)
sample_names(colon_phy) <- sample_data(colon_phy)$Animal_number
colon_phy <- prune_taxa(taxa_sums(colon_phy) > 0, colon_phy)
colon_ord <- ordinate(colon_phy, method= "RDA", distance= "bray", type="samples")

#run the procrustes
proc <- procrustes(poo_ord, colon_ord)
procplot <- plot(proc)

#test significance
proctest <- protest(poo_ord, colon_ord, scores = "sites")
proctest

Colon v faeces permutation tests

D <- distance(colon_poo_phy, method = "bray", type = "samples" )
D <- as.data.frame(melt(as.matrix(D)))
D$Sample_pair <- paste(D$Var1, D$Var2, sep="-")
M <- as(sample_data(colon_poo_phy), 'data.frame')

colnames(D)[1] <- "Sequence_sample_code"
D$ID.A <-"NA"
D$ID.A <- M[match(D$Sequence_sample_code, M$Sequence_sample_code), "Animal_number"]
D$gut.A <-"NA"
D$gut.A <- M[match(D$Sequence_sample_code, M$Sequence_sample_code), "Gut_section"]
D$date.A <-"NA"
D$date.A <- M[match(D$Sequence_sample_code, M$Sequence_sample_code), "Collection_date"]
colnames(D)[1] <- "Var1"
colnames(D)[2] <- "Sequence_sample_code"
D$ID.B <-"NA"
D$ID.B <- M[match(D$Sequence_sample_code, M$Sequence_sample_code), "Animal_number"]
D$gut.B <-"NA"
D$gut.B <- M[match(D$Sequence_sample_code, M$Sequence_sample_code), "Gut_section"]
D$date.B <-"NA"
D$date.B <- M[match(D$Sequence_sample_code, M$Sequence_sample_code), "Collection_date"]
colnames(D)[2] <- "Var2"

#within-individual colon v poo
D_colon_poo <- D[D$ID.A==D$ID.B,]
D_colon_poo <- D_colon_poo[D_colon_poo$gut.A!=D_colon_poo$gut.B,]
D_colon_poo$comparison <- "colon-faeces"

#between individual colon-colon
D_colon_colon <- D[D$ID.A!=D$ID.B,]
D_colon_colon <- D_colon_colon[D_colon_colon$date.A==D_colon_colon$date.B,]
D_colon_colon <- subset(D_colon_colon, gut.A=="COLON" & gut.B=="COLON")
D_colon_colon$comparison <- "colon-colon"
#between-individual poo-poo
D_poo_poo <- D[D$ID.A!=D$ID.B,]
D_poo_poo <- D_poo_poo[D_poo_poo$date.A==D_poo_poo$date.B,]
D_poo_poo <- subset(D_poo_poo, gut.A=="POO" & gut.B=="POO")
D_poo_poo$comparison <- "faeces-faeces"

#combine
D_comb <- rbind(rbind(D_colon_poo, D_colon_colon),D_poo_poo)
D_comb$comparison <- as.factor(D_comb$comparison)

#test
#set.seed(1235)
#1. colon-colon vs colon-poo
D_comb_sub1 <- subset(D_comb, comparison%in%c("colon-colon", "colon-faeces"))
u.obs <- wilcox.test(value~comparison, data=D_comb_sub1)
u.obs.stat <- u.obs$statistic#
nperm <-1000
perm1 <- data.frame(permutation=1:nperm, u.stat=NA)
lose<-"value"

for(i in 1:nperm) {
 d <- D_comb_sub1[,!names(D_comb_sub1)%in% lose]
 d$dist.rand <- sample(D_comb_sub1$value, length(D_comb_sub1$value), replace = F)
 x <- wilcox.test(dist.rand ~ comparison, data=d)
 perm1$u.stat[i] <- x$statistic
}

hist(perm1$u.stat)
abline(v=u.obs$statistic, col="red", lwd=2, lty=2)

perm1$result <- ifelse(perm1$u.stat>u.obs$statistic,1,0)
p<- 2*(mean(perm1$result))
p#0

#2. faeces-faeces vs colon-poo
D_comb_sub2 <- subset(D_comb, comparison%in%c("faeces-faeces", "colon-faeces"))
u.obs <- wilcox.test(value~comparison, data=D_comb_sub2)
u.obs.stat <- u.obs$statistic#
nperm <-1000
perm1 <- data.frame(permutation=1:nperm, u.stat=NA)
lose<-"value"

for(i in 1:nperm) {
 d <- D_comb_sub2[,!names(D_comb_sub2)%in% lose]
 d$dist.rand <- sample(D_comb_sub2$value, length(D_comb_sub2$value), replace = F)
 x <- wilcox.test(dist.rand ~ comparison, data=d)
 perm1$u.stat[i] <- x$statistic
}

hist(perm1$u.stat)
abline(v=u.obs$statistic, col="red", lwd=2, lty=2)

perm1$result <- ifelse(perm1$u.stat<u.obs$statistic,1,0)
p<- 2*(mean(perm1$result))
p#0


#plot
colon_poo_plot <- ggplot(D_comb, aes(x=comparison, y=value)) +
 geom_point(alpha=0.2)+
 geom_violin(colour="grey", fill="grey",alpha=0.5)+
 theme_light() +
 ylab("Bray-Curtis distance") + xlab("Comparison") +
 theme(axis.title = element_text(size=16),
 axis.text = element_text(size=14)) +
 scale_x_discrete(labels=c("colon-colon \n different mouse", "colon-faeces \n same mouse", "faeces-faeces \n different mouse"))+
 geom_bracket(xmin = c("colon-colon", "colon-faeces"),
 xmax = c("colon-faeces", "faeces-faeces"),
 y.position = c(1.05, 1.1), label = c("**", "**"))

#ggsave2('FigS9_Colon_poo_permutations_procrustes_plots.jpeg', colon_poo_plot)
