# Supplementary material for: Parasite–gut microbiota associations in wild wood mice (Apodemus sylvaticus)
Source: Front Microbiol. 2024 Nov 18;15:1440427. doi: 10.3389/fmicb.2024.1440427 (PMC11608965; doi:10.3389/fmicb.2024.1440427)
Supplement: Supplementary file 1 [file Data_Sheet_1.zip › 1440427_Knowles_Supplementary_Methods_and_Results.DOCX]

Parasite-gut microbiota associations in wild wood mice (*Apodemus sylvaticus*)

Marsh, K.J.^1^, Raulo, A.R.^2^, Webster, J.P.^1^, Knowles, S.C.L.^1,2^*

**Supplementary Materials**

**Methods**


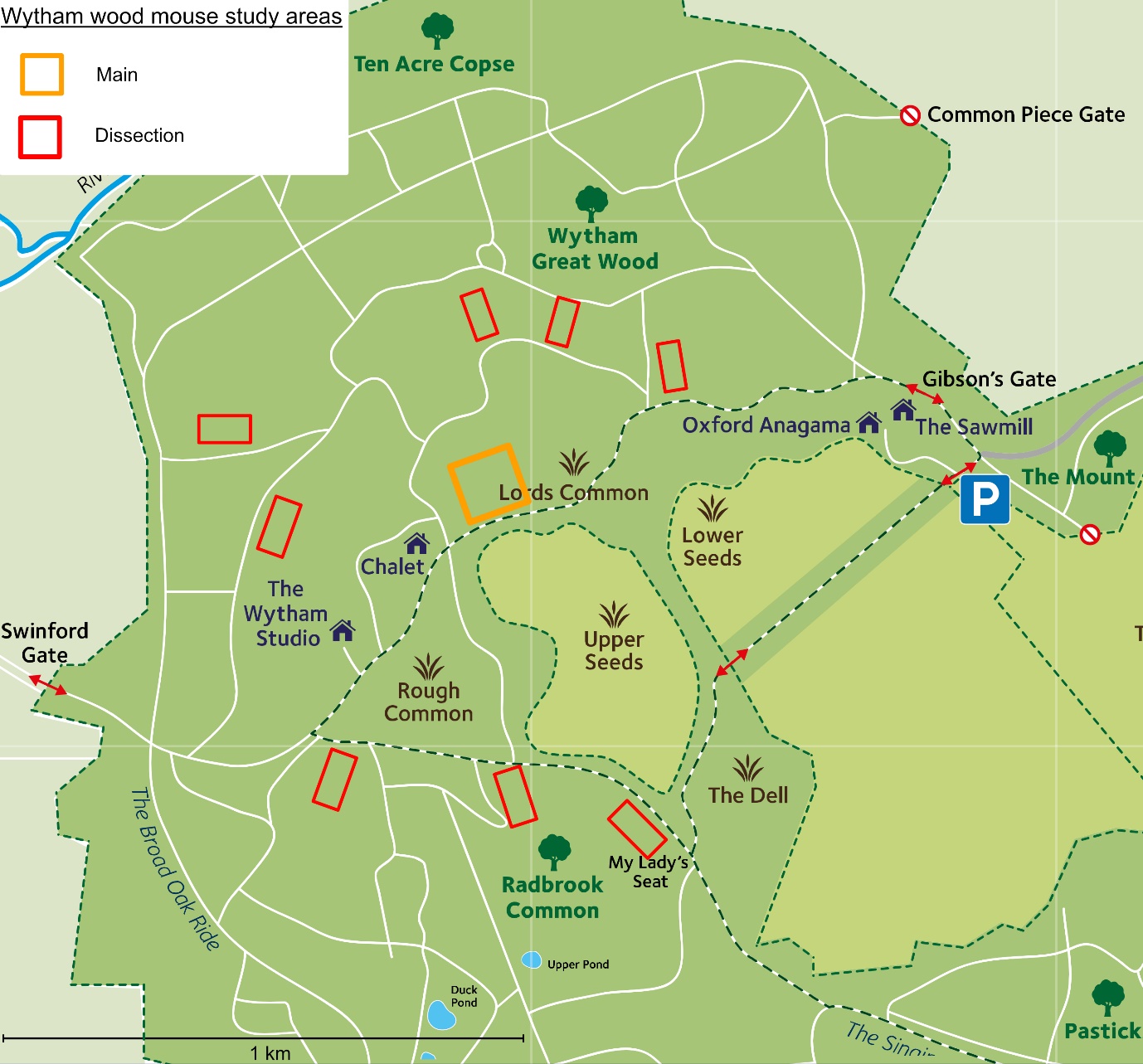


**Figure S1.** A map of Wytham woods showing the locations of trapping grids used for the non-invasive longitudinal study (main) and the dissection grids. These grids were sampled fortnightly between October 2015-18 (main) and 2017-18 (dissection).

**Figure S2. Correlations among covariates in each dataset.** Data on parasite burden and microbiome profiles were obtained from mice trapped between October 2015-18 in Wytham woods. Correlations among covariates used to model associations among parasites and the microbiota are shown, with the distributions of each term displayed along the diagonal. A) Worm counts (log+1 transformed, Hymenolepis presence/absence) and microbiome profiles per gut section in n=50 mice quantified in a dissection study. B) Parasite burden (log+1 eggs/oocysts per gram) and microbiome profiles of n=221 faecal samples from 105 mice collected in a longitudinal study.


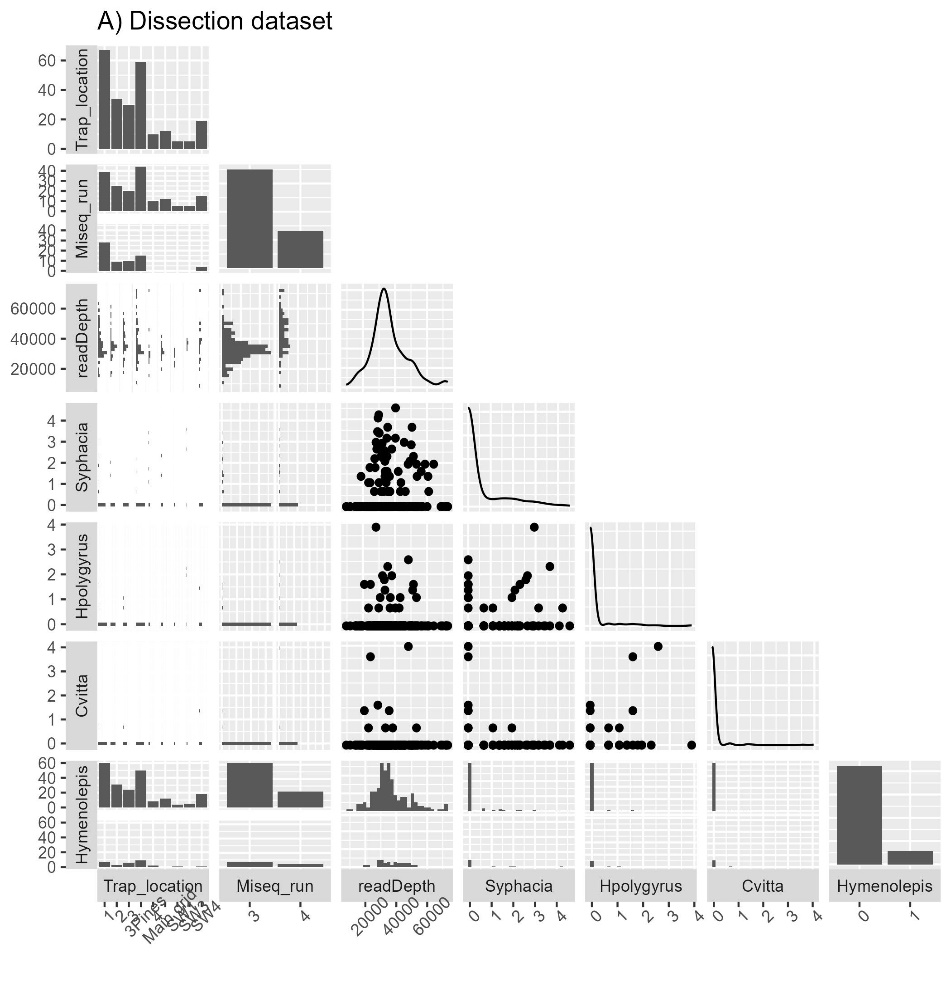

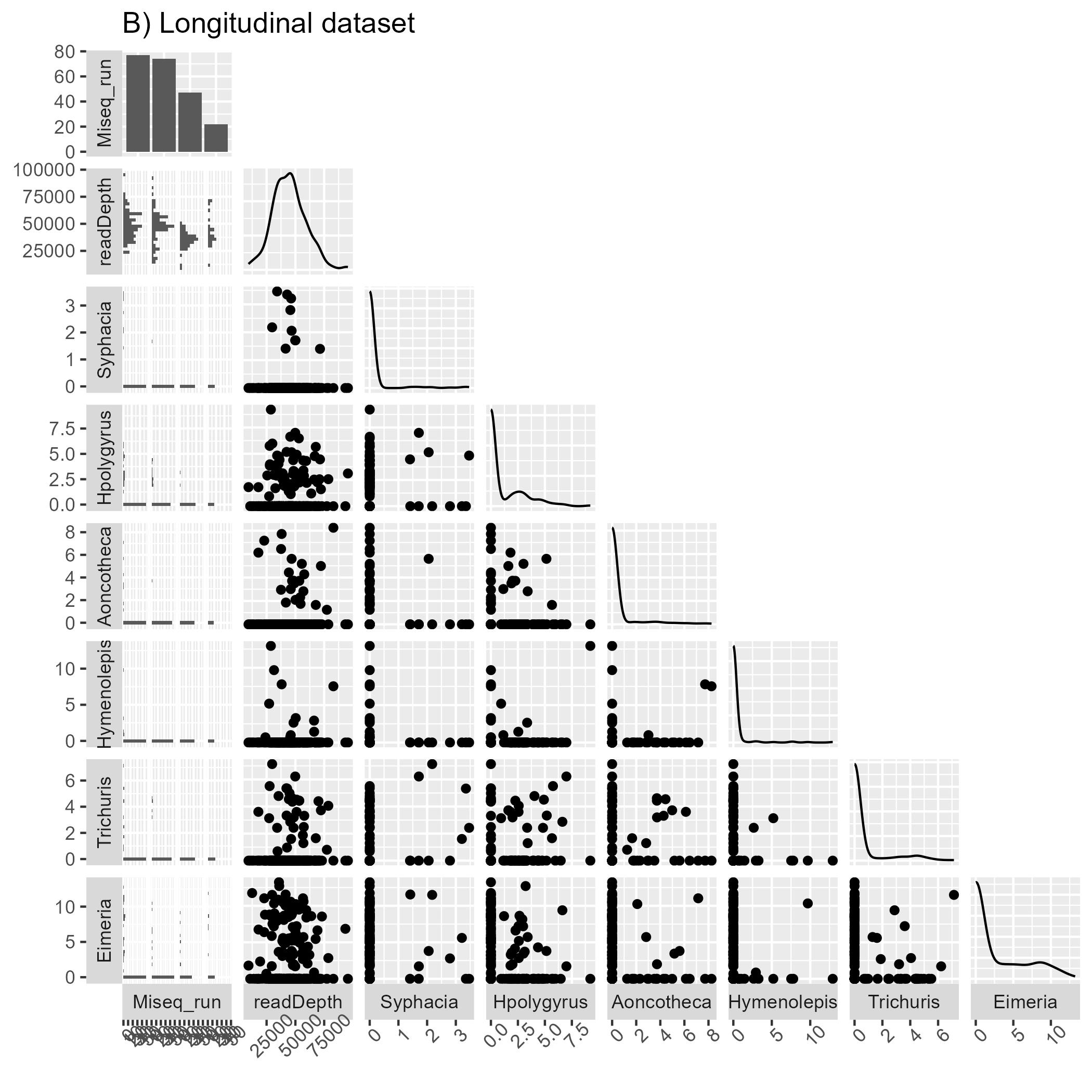


**Table S1. Taxonomy of putative contaminant ASVs identified in decontam.** A total of 159 ASVs from 35 bacterial families were identified as contaminants using prevalence in negative controls in the R package ‘decontam’. The families that these contaminant ASVs belonged to, and the total number of ASVs assigned to these families overall are shown.

| **Family** | **Number of contaminant ASVs** | **Number of total ASVs** |
| --- | --- | --- |
| Lachnospiraceae | 65 | 3097 |
| Ruminococcaceae | 36 | 1600 |
| Clostridiales (vadin BB60) | 8 | 312 |
| Muribaculaceae | 6 | 1039 |
| Lactobacillaceae | 5 | 285 |
| Enterobacteriaceae | 3 | 116 |
| Bacillaceae | 2 | 20 |
| Bifidobacteriaceae | 2 | 19 |
| Comamonadaceae | 2 | 30 |
| Coriobacteriaceae | 2 | 205 |
| Prevotellaceae | 2 | 153 |
| Pseudomonadaceae | 2 | 28 |
| Actinomycetaceae | 1 | 8 |
| Coxiellaceae | 1 | 15 |
| Family XI | 1 | 11 |
| Family XIII | 1 | 87 |
| Flavobacteriaceae | 1 | 23 |
| Hydrogenophilaceae | 1 | 2 |
| Leuconostocaceae | 1 | 9 |
| Listeriaceae | 1 | 6 |
| Microbacteriaceae | 1 | 62 |
| Moraxellaceae | 1 | 15 |
| Mycoplasmataceae | 1 | 36 |
| Pasteurellaceae | 1 | 9 |
| Porphyromonadaceae | 1 | 39 |
| Propionibacteriaceae | 1 | 16 |
| Rhodobacteraceae | 1 | 28 |
| Rickettsiales (incertae sedis) | 1 | 37 |
| Rikenellaceae | 1 | 297 |
| Staphylococcaceae | 1 | 16 |
| Streptococcaceae | 1 | 56 |
| Thermaceae | 1 | 6 |
| Unknown Family | 1 | 58 |
| Veillonellaceae | 1 | 32 |
| YNPFFP1 | 1 | 1 |

**Results**


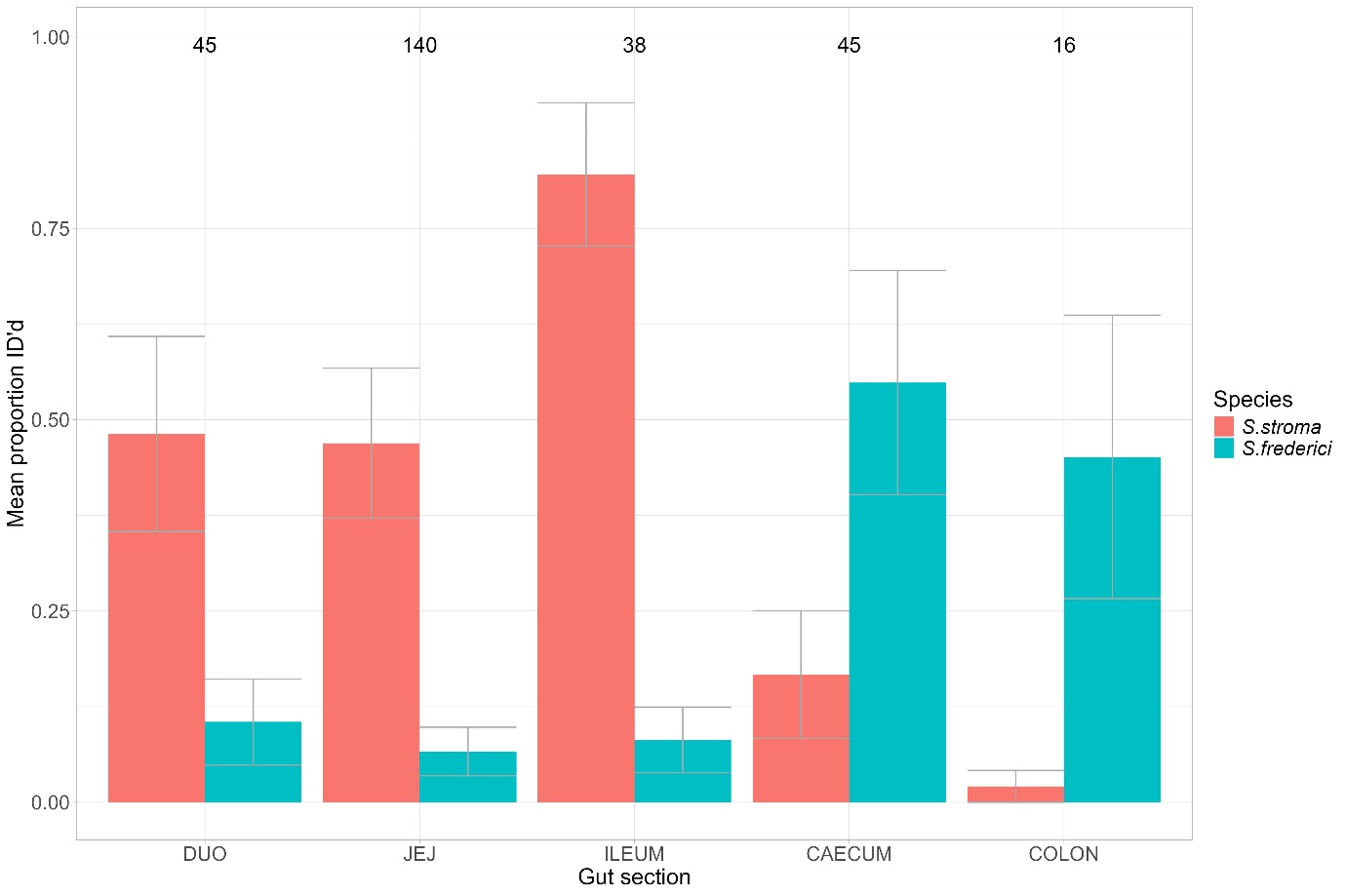
**Figure S3. The niches of two *Syphacia* species within the wood mouse gut.** Worm counts from dissections of the GI tract of wood mice (n=50) were split by gut section (Duodenum, jejunum, ileum, caecum and colon). When *Syphacia* was present, a subset of worms were stored per mouse/gut section for further identification to species level (total number of worms per gut section used for identification shown above bars). The average proportion of *Syphacia* that were identified as *S. stroma* (red) and *S. frederici* (blue) are shown with the standard error of the mean.


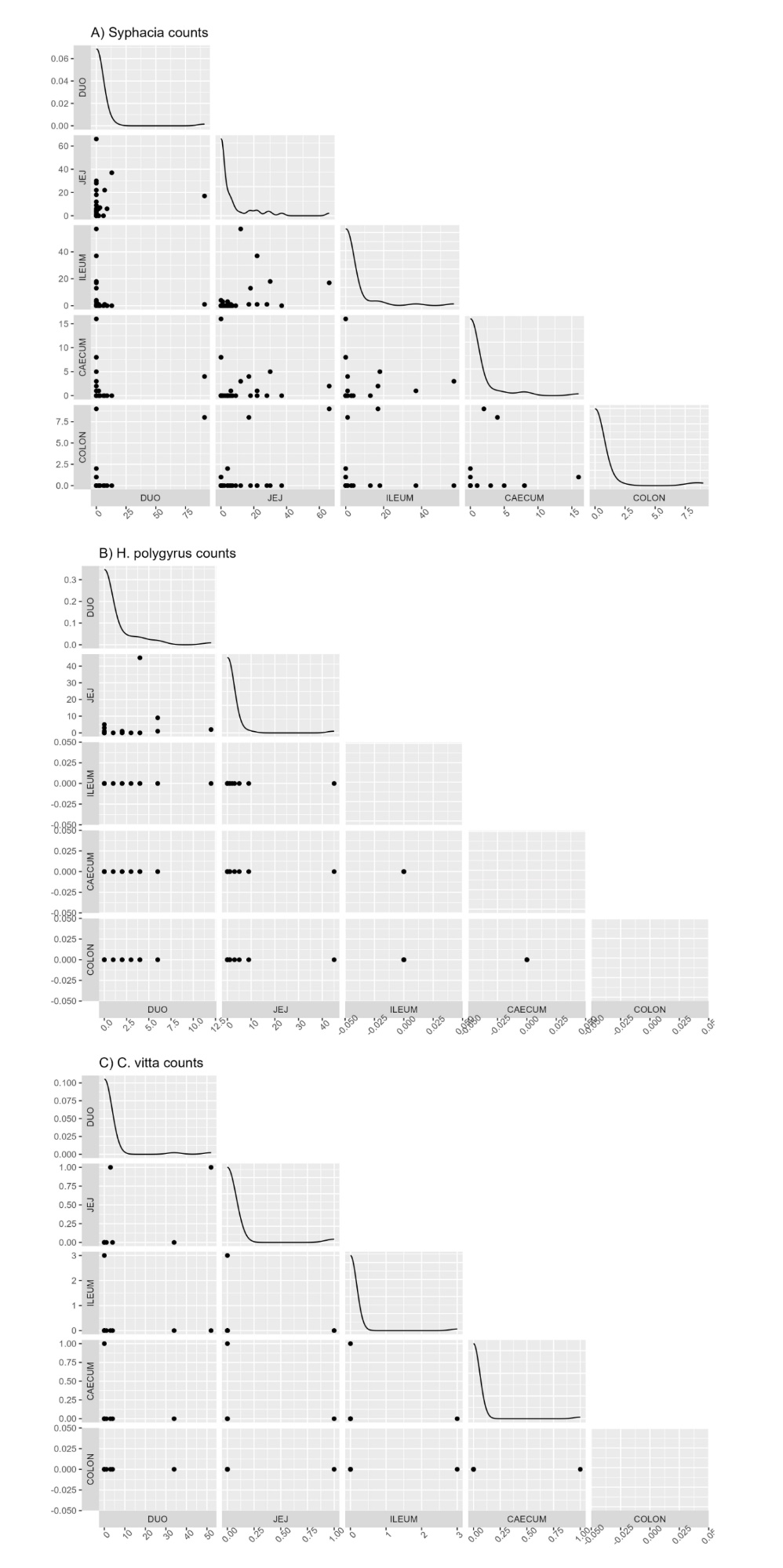


**Figure S4. Correlations in parasites counts across gut sections.** Worm counts from dissections of the GI tract of wood mice (n=50) were split by gut section (Duodenum, jejunum, ileum, caecum and colon). The correlations between counts among gut sections per mouse for a) Syphacia spp. b) H. polygyrus and c) C. viita are shown, with the distributions of counts per gut section along the diagonal.


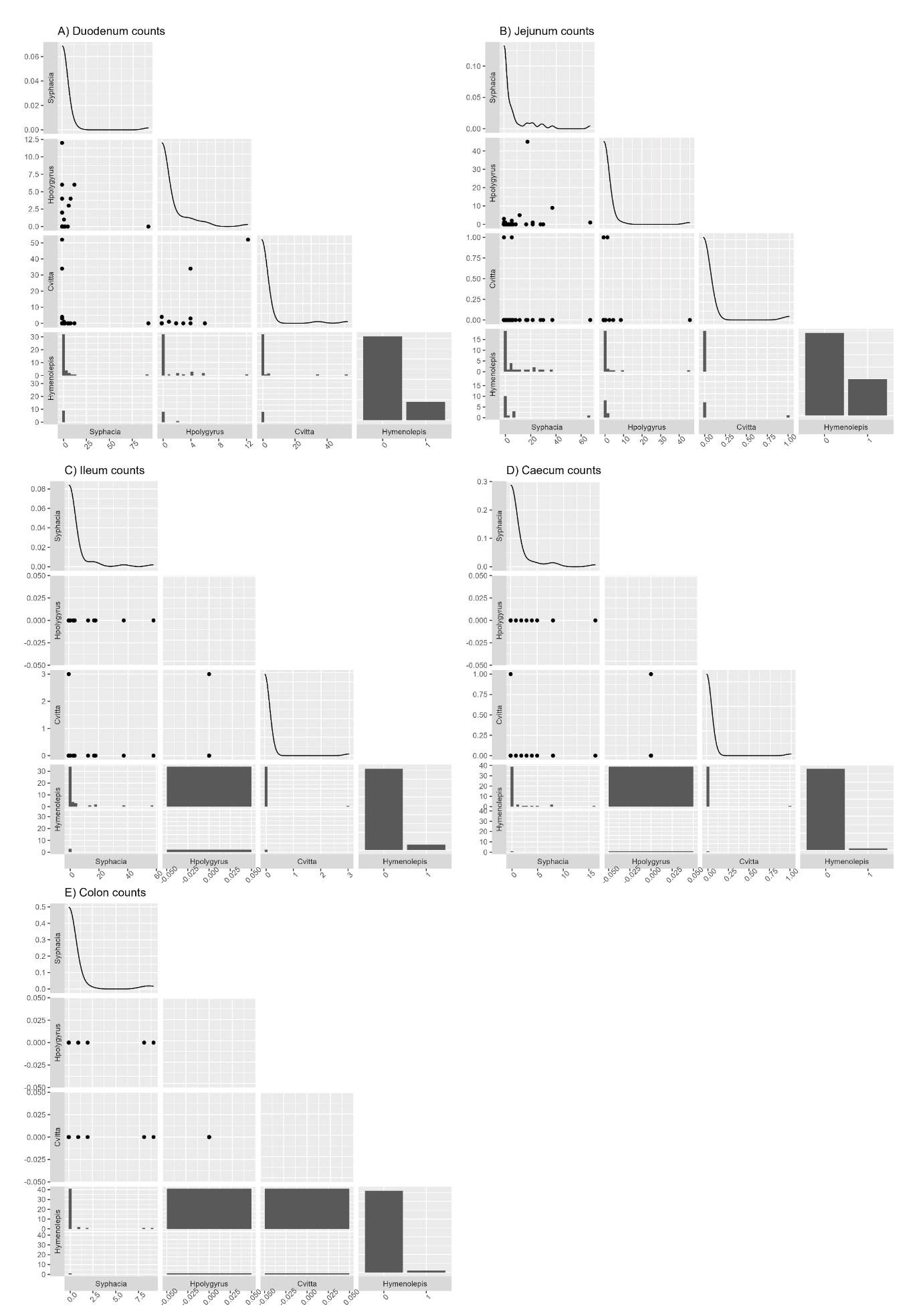


**Figure S5. Correlations among parasite counts within gut sections.** Worm counts from dissections of the GI tract of wood mice (n=50) were split by gut section (Duodenum, jejunum, ileum, caecum and colon). The correlations between counts of parasites within gut sections a) Duodenum b) Jejunum c) Ileum d) cecum and e) colon are shown, with the distributions of counts (presence/absence of Hymenolepis) per gut section along the diagonal.


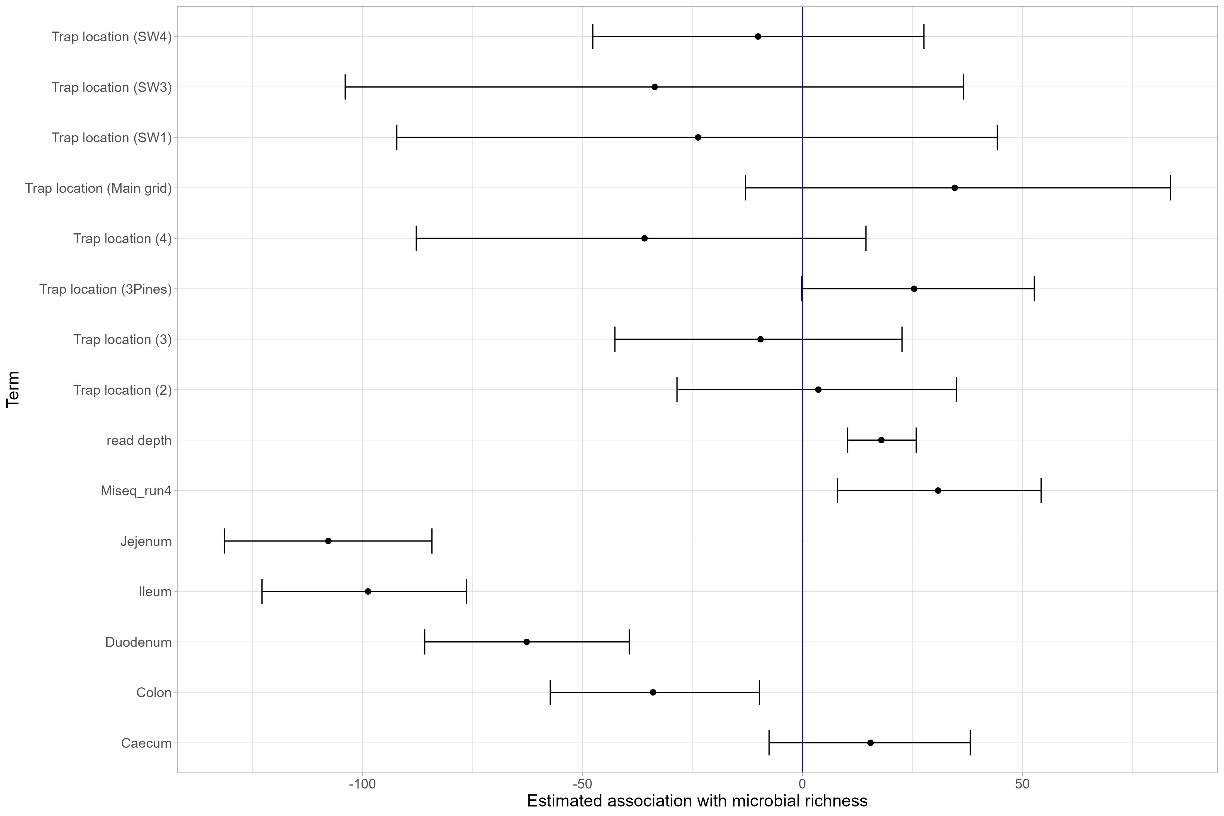


**Figure S6. Microbial (ASV) richness along the gastrointestinal tract.** Richness of the bacterial microbiota in different sections of the GI tract was estimated from a dissection study of wild wood mice (n=50). Samples were taken from each gut section (duodenum, jejunum, ileum, caecum and colon), as well as faecal samples from the bedding material of the same mice for comparison. A Bayesian mixed model was used to assess differences in microbial richness according to sample type (sampling location along the gut, or faecal sample) as well as covariates. The posterior means of each term in the Bayesian model with 95% credible intervals are shown. Faecal samples were used as the reference category for comparison with each gut section.


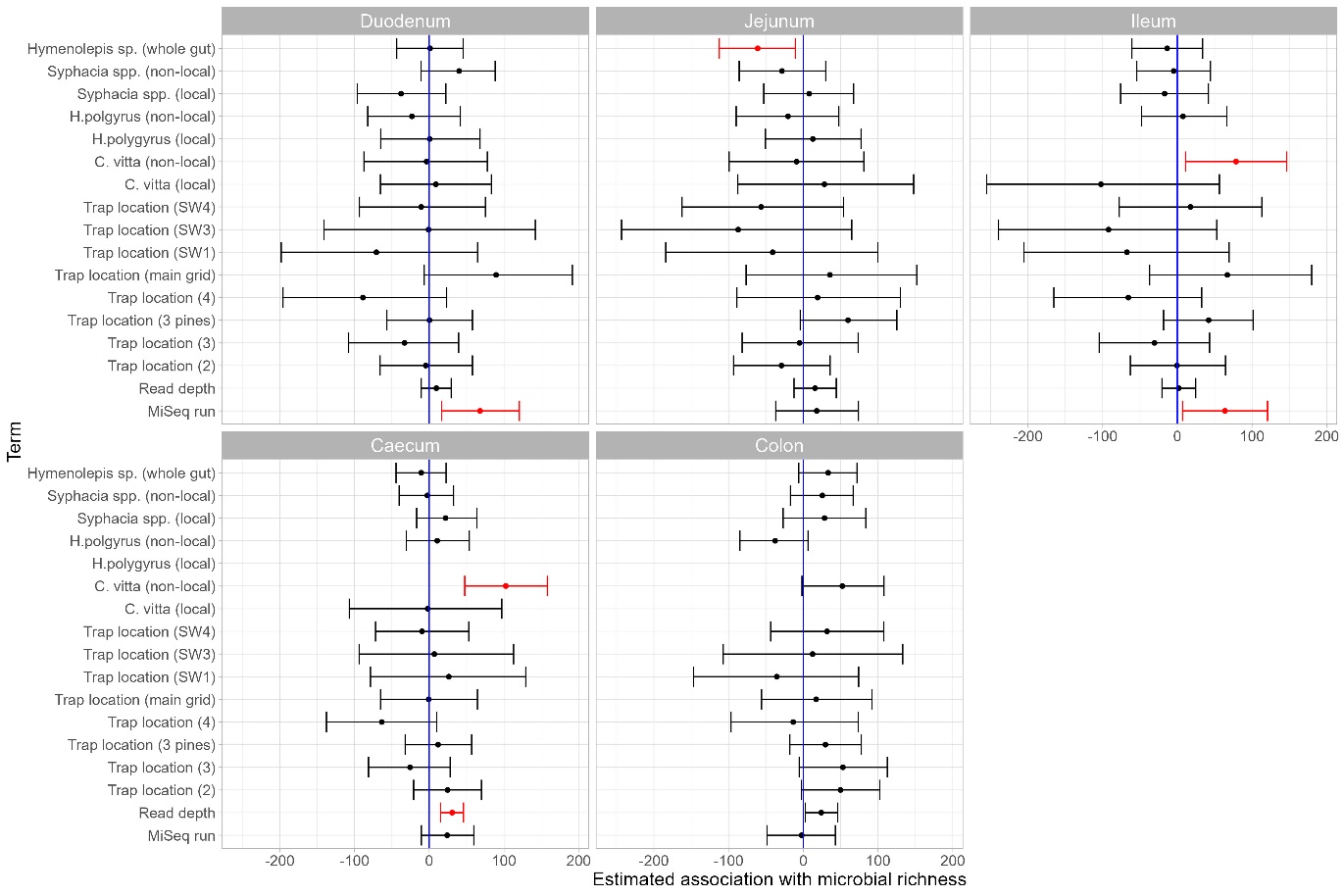


**Figure S7. Local and non-local associations between gut microbial richness and parasite infection.** A series of Bayesian regression models were run, one per gut section (duodenum, jejunum, ileum, caecum and colon) to assess the associations between parasite presence and microbial richness in wood mice (n=50). In each model, parasite presence was divided into ‘local’ in the gut section being tested and ‘non-local’ across all other gut sections. Trap location, MiSeq run and read depth were included as covariates. Model estimates and 95% credible intervals (CIs) are shown for each term. Terms with CIs not overlapping 0 are considered potentially important and shown with red bars.

**Figure S8. The similarity in microbiota composition between colon and faecal samples.** Bray-Curtis distances between colon and faecal samples from a dissection study of wild wood mice (n=50). Samples were taken from the colon and faecal samples from the bedding material of the same mice for comparison (‘colon-faeces’). The colon and faecal samples taken from the same mouse were more similar (lower Bray-Curtis dissimilarity) than samples of the same type taken from different mice caught at the same time (‘colon-colon’ / ‘faeces-faeces’). Significant differences between groups was tested by permutational Wilcoxon tests and indicated by ** (p<0.001). A procrustes analysis was also performed on Bray-Curtis distances between paired colon and feacal samples from 39 mice (m_12_^2^=0.809, p=0.005).


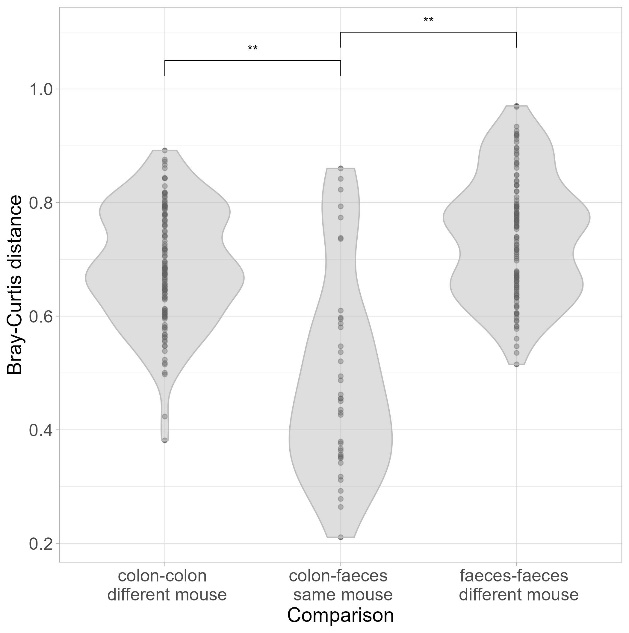

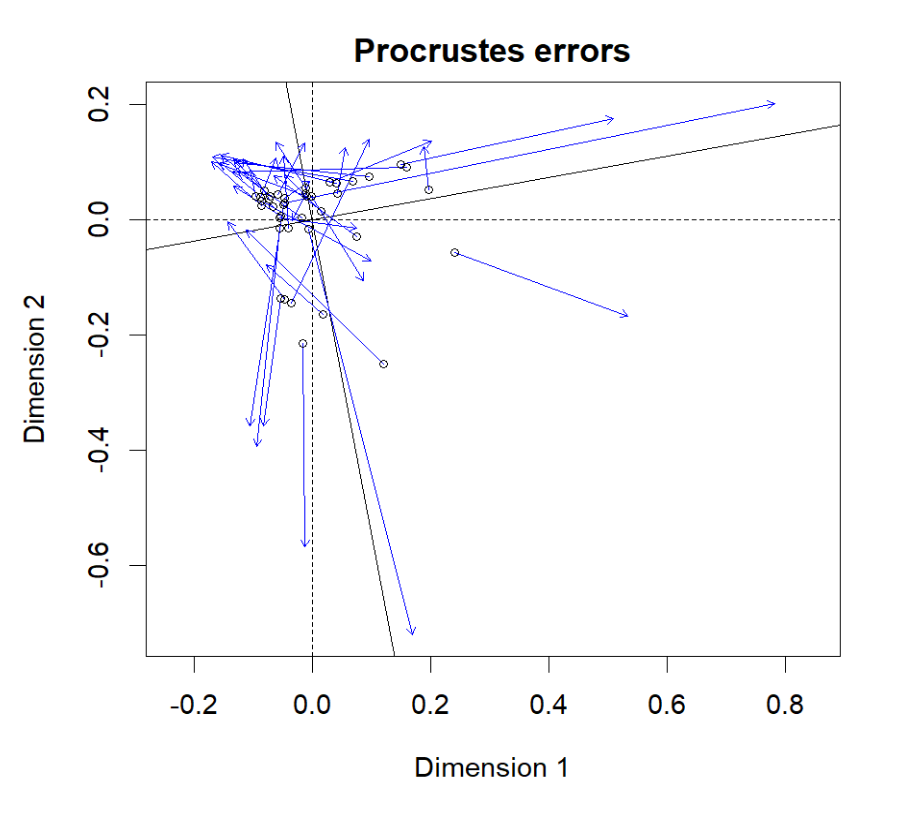


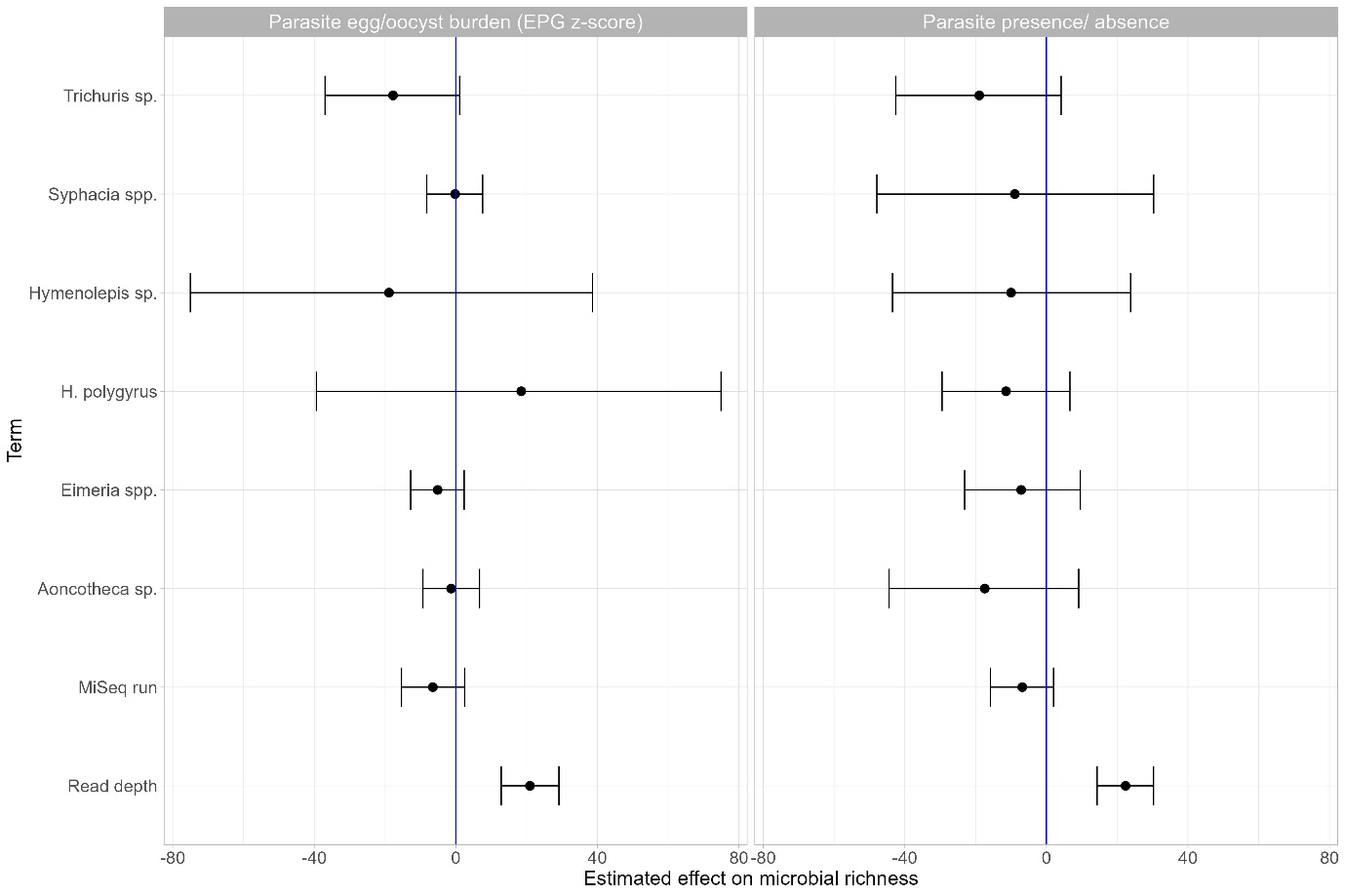


**Figure S9. Associations between gut parasite infection and microbial richness in the longitudinal study.** Bayesian mixed models were used to assess the relationship between parasite infection and microbial richness of faecal samples collected from wood mice (n=221). One model used quantitative measures of egg/oocyst burdens (z-scores of EPG, including zeros) as predictors, and the other used parasite presence/absence. Read depth and MiSeq run were included as covariates, with individual ID used as a random factor. Model estimates and 95% credible intervals are shown for each term.


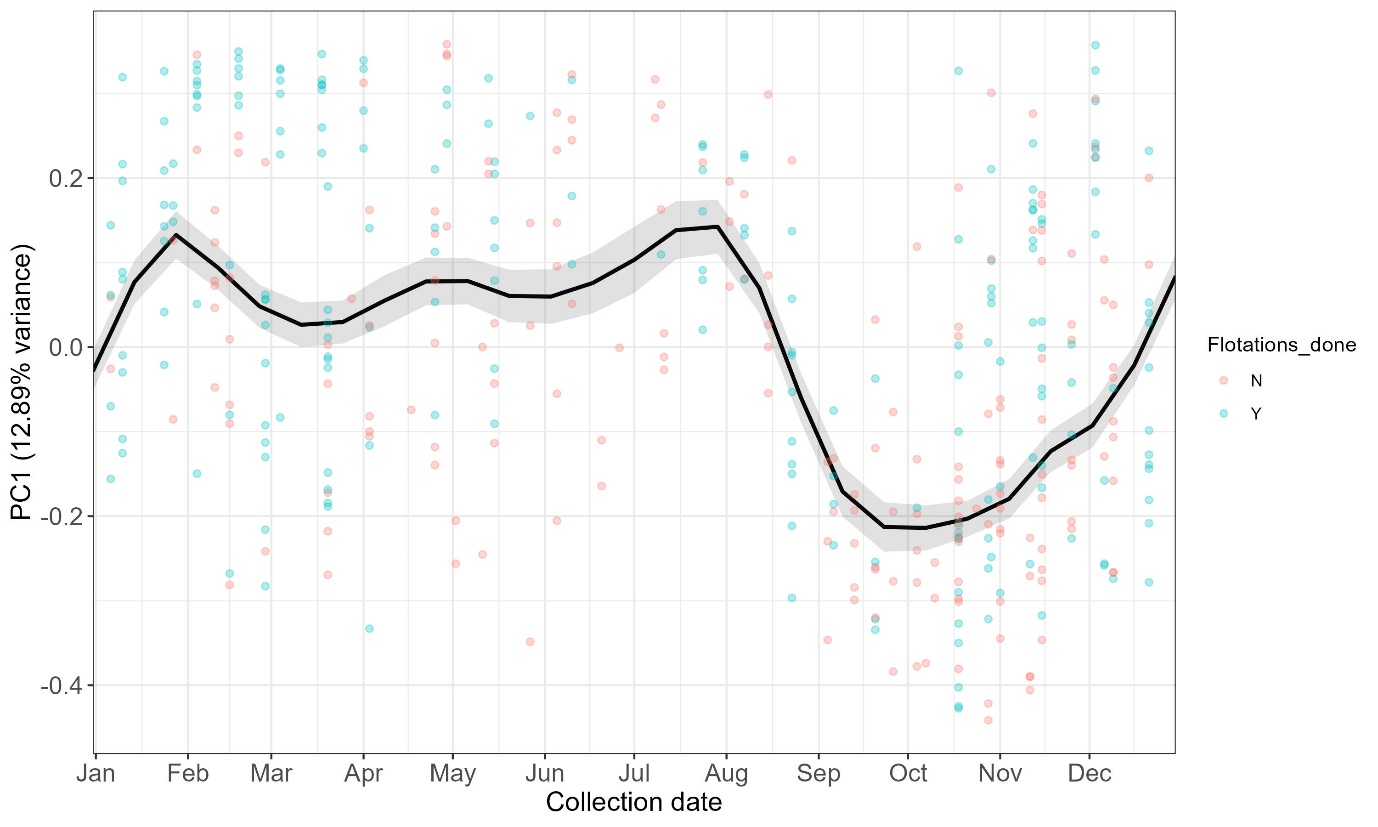


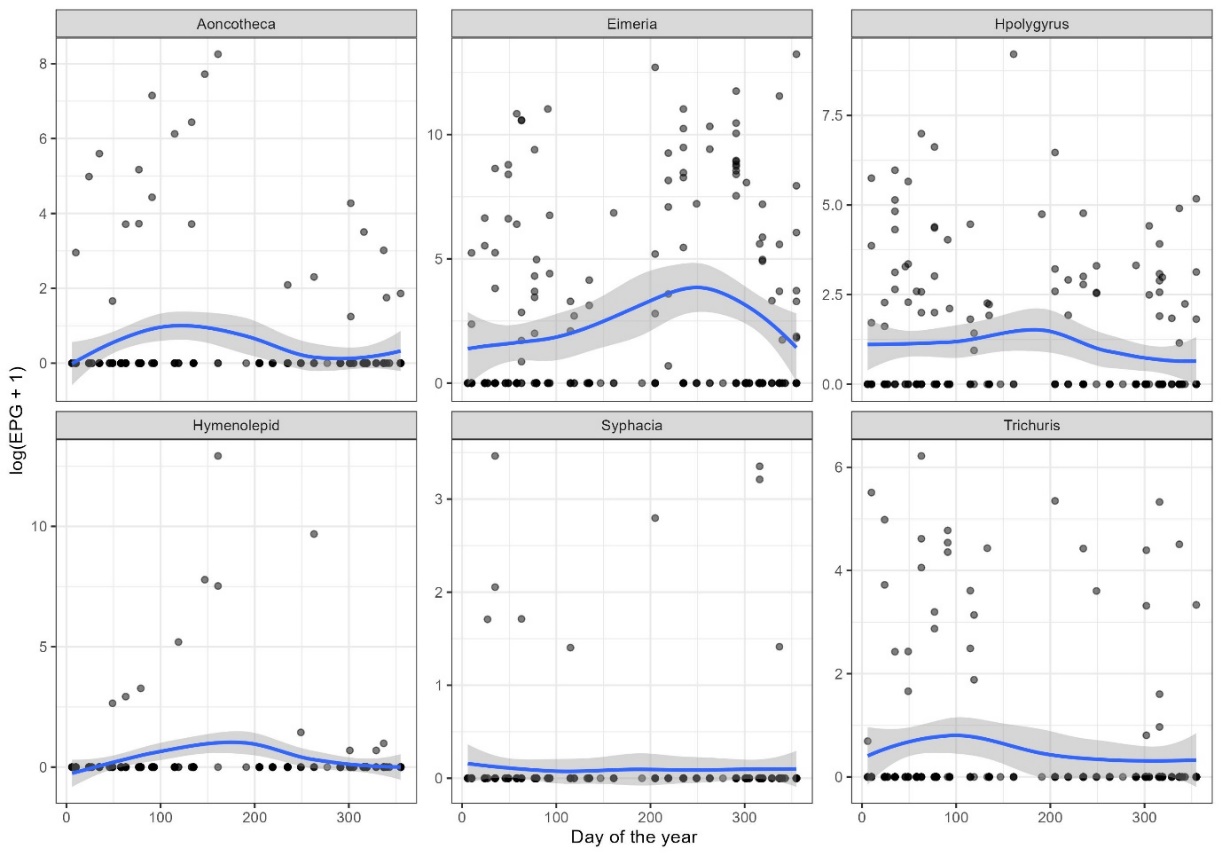
**Figure S10. Seasonality in the gut microbiota of wood mice in Wytham woods.** Faecal samples were collected from live-trapped mice between October 2015-18 and the microbiota profiled using 16S amplicon sequencing (n=448). The first axis of a principle coordinates analyses on Bray-Curtis distances among these samples (PC1) was used as the response in a general additive mixed model of seasonality in this variable. Day of the year (cyclic cubic spline_ was the main predictor, with year included as a covariate and mouse ID as a random term to control for repeated measures. Fitted values and their standard error from this model are plotted along with raw values. The colour of the data points indicate whether faecal flotations were also conducted on the samples and used in analyses of microbiome-parasite associations (n=223).

**Figure S11. Seasonality in parasite burdens of wild wood mice.** Faecal samples were collected from live-caught mice between October 2015-18 and subsequently used for faecal flotations to quantify parasite burdens as eggs per gram (EPG; n=221). The seasonality in parasite burden (log(EPG+1) per parasite species is shown with a loess smoothing function.

**Table S2. Results of a Bayesian mixed model assessing the association between gut microbial richness and parasite infection.** The gastrointestinal (GI) tract of wood mice (n=50) was divided into five sections (duodenum, jejunum, ileum, caecum and colon), with microbial richness and parasite burden quantified per gut section. A Bayesian mixed model was used to assess the association between microbial richness and parasite presence throughout the GI tract with an interaction term for gut section: each parasite presence. Animal ID was used as a random term (as 5 gut section per animal were used), with trapping location, read depth and MiSeq run included as covariates. This table shows the parameter estimates and 95% credible intervals (CI’s) from the model, with the following reference categories per term; Trap location (1), Gut section (caecum), MiSeq run (1), parasite absence (0). Terms with CI’s not overlapping 0 are highlighted with bold font.

|  | Model term | Posterior mean | Lower 95% CI | Upper 95% CI |
| --- | --- | --- | --- | --- |
|  | Intercept | 222.8259 | 195.5528 | 251.7486 |
| Gut section | **Duodenum** | **-68.7889** | **-96.0174** | **-41.7037** |
|  | **Jejenum** | **-106.773** | **-137.809** | **-76.1815** |
|  | **Ileum** | **-114.531** | **-141.235** | **-89.3464** |
|  | **Colon** | **-50.0766** | **-74.8536** | **-25.5812** |
| Parasite presence | *H. polygyrus* | -8.97548 | -41.0406 | 23.35671 |
|  | *C. vitta* | -30.5652 | -74.1495 | 13.24457 |
|  | *Syphacia* spp. | -3.42144 | -46.3253 | 39.68803 |
|  | *Hymenolepis* sp. | -55.0725 | -168.038 | 66.5083 |
| Gut section: parasite presence interaction | Duodenum: *Syphacia* spp. | -20.9999 | -82.8553 | 42.06943 |
|  | Jejenum: *Syphacia* spp. | -5.21344 | -57.7611 | 50.25646 |
|  | Ileum: *Syphacia* spp. | -14.7306 | -70.6429 | 42.48768 |
|  | Colon: *Syphacia* spp. | -6.79023 | -74.5937 | 57.07713 |
|  | Duodenum: *Hymenolepis* sp. | 42.03383 | -84.0088 | 165.9286 |
|  | Jejenum: *Hymenolepis* sp. | 17.08811 | -106.908 | 136.8009 |
|  | Ileum: *Hymenolepis* sp. | 100.9718 | -37.7325 | 233.9099 |
|  | Colon: *Hymenolepis* sp. | 10.74746 | -141.976 | 160.3679 |
| Covariates | **Read depth** | **17.09294** | **8.363813** | **25.82172** |
|  | **Miseq run** | **29.00717** | **1.349525** | **55.88484** |
|  | Trap location (2) | -2.07854 | -36.8354 | 32.61431 |
|  | Trap location (3) | -9.32871 | -47.3275 | 26.31955 |
|  | Trap location (3Pines) | 24.54379 | -6.50114 | 55.16594 |
|  | Trap location (4) | -50.1751 | -105.718 | 3.680875 |
|  | Trap location (Main grid) | 27.17594 | -23.9439 | 76.68698 |
|  | Trap location (SW1) | -56.208 | -134.334 | 22.2954 |
|  | Trap location (SW3) | -47.7658 | -127.019 | 30.59713 |
|  | Trap location (SW4) | -23.3765 | -66.0218 | 19.93382 |

**Table S3. The local and non-local associations between parasite abundance and gut section-specific microbial composition full results.** Results are from a series of partial redundancy analyses (pRDAs) predicting gut microbial composition per gut section in 50 wood mice. The overall model included interaction terms for gut section: parasite presence/absence and a conditional term for individual ID to control for repeated measures. The significance of each individual parasite term and gut section was determined in a model without interaction terms. Models per gut section included parasite presence (species-specific local and non-local presence) as predictors. False discovery rate (FDR) adjusted p-values are shown for gut section-specific models. P-values <0.1 marked with an . , <0.05 with * and p<0.01 with **.

| Model | Term | F | df | p-value | Adjusted p-value |
| --- | --- | --- | --- | --- | --- |
| Overall  F_(21,170)_=6.32, P=0.001,  Adjusted R^2^=0.252 | *Syphacia* spp. presence : Gut section | 1.35 | 4 | 0.198 | n/a |
|  | *H.polygyrus* presence : Gut section | 3.22 | 1 | 0.052 . | n/a |
|  | *Hymenolepis* sp. presence: Gut section | 1.36 | 4 | 0.189 | n/a |
|  | *C. vitta* presence : Gut section | 0.43 | 3 | 0.886 | n/a |
|  | *Syphacia* spp. presence | 0.37 | 1 | 0.776 | n/a |
|  | *H. polygyrus* presence | 0.98 | 1 | 0.336 | n/a |
|  | *Hymenolepis* spp. presence | 1.39 | 1 | 0222 | n/a |
|  | *C. vitta* presence | 1.36 | 1 | 0.234 | n/a |
|  | **Gut section** | **23.14** | **4** | **0.001 **** | n/a |
|  | Read depth | 2.39 | 1 | 0.072 . | n/a |
| Duodenum  F_(17,30)_=2.24, p=0.001, adjusted R^2^=0.311 | *Syphacia* spp. local presence | 1.86 | 1 | 0.125 | 0.625 |
|  | *Syphacia* spp. non-local presence | 1.68 | 1 | 0.162 | 0.648 |
|  | *H. polygyrus* local presence | 1.83 | 1 | 0.154 | 0.308 |
|  | *H. polygyrus* non-local presence | 0.79 | 1 | 0.453 | 1.000 |
|  | *Hymenolepis* sp. (overall) presence | 0.19 | 1 | 0.963 | 1.000 |
|  | *C. vitta* local presence | 0.71 | 1 | 0.532 | 1.000 |
|  | *C. vitta* non-local presence | 2.51 | 1 | 0.070 . | 0.350 |
|  | Read depth | 2.49 | 1 | 0.080 . | 0.320 |
|  | MiSeq run | 0.53 | 1 | 0.664 | 1.000 |
|  | **Trap location** | **2.39** | **8** | **0.004 **** | **0.020 *** |
| Jejenum  F_(17,30)_ =1.63, p=0.038, adjusted R^2^=0.185 | *Syphacia* spp. local presence | 0.11 | 1 | 0.989 | 1.000 |
|  | *Syphacia* spp. non-local presence | 2.44 | 1 | 0.075 . | 0.375 |
|  | *H. polygyrus* local presence | 0.32 | 1 | 0.819 | 0.819 |
|  | *H. polygyrus* non-local presence | 0.82 | 1 | 0.456 | 1.000 |
|  | *Hymenolepis* sp. (overall) presence | 0.76 | 1 | 0.479 | 1.000 |
|  | *C. vitta* local presence | 0.36 | 1 | 0.781 | 1.000 |
|  | *C. vitta* non-local presence | 1.60 | 1 | 0.165 | 0.660 |
|  | Read depth | 1.93 | 1 | 0.109 | 0.327 |
|  | MiSeq run | 1.23 | 1 | 0.277 | 1.000 |
|  | Trap location | 1.73 | 8 | 0.062 . | 0.248 |
| Ileum  F_(16,32)_=1.65, p=0.043, adjusted R^2^=0.179 | *Syphacia* spp. local presence | 0.57 | 1 | 0.575 | 1.000 |
|  | *Syphacia* spp. non-local presence | 0.60 | 1 | 0.545 | 1.000 |
|  | *H. polygyrus* non-local presence | 0.47 | 1 | 0.650 | 1.000 |
|  | *Hymenolepis* sp. (overall) presence | 1.69 | 1 | 0.171 | 0.855 |
|  | *C. vitta* local presence | 0.94 | 1 | 0.400 | 1.000 |
|  | *C. vitta* non-local presence | 0.60 | 1 | 0.518 | 1.000 |
|  | Read depth | 4.44 | 1 | 0.020 * | 0.100 |
|  | MiSeq run | 1.13 | 1 | 0.310 | 1.000 |
|  | Trap location | 1.67 | 8 | 0.093 . | 0.279 |
| Caecum  F_(16,32)_=0.99, p=0.509, adjusted R^2^=0.00 | *Syphacia* spp. local presence | 0.69 | 1 | 0.756 | 1.000 |
|  | *Syphacia* spp. non-local presence | 0.61 | 1 | 0.817 | 1.000 |
|  | *H. polygyrus* non-local presence | 1.15 | 1 | 0.300 | 1.000 |
|  | *Hymenolepis* sp. (overall) presence | 0.81 | 1 | 0.600 | 1.000 |
|  | *C. vitta* local presence | 0.39 | 1 | 0.910 | 1.000 |
|  | *C. vitta* non-local presence | 0.49 | 1 | 0.941 | 1.000 |
|  | Read depth | 0.81 | 1 | 0.581 | 0.682 |
|  | MiSeq run | 0.73 | 1 | 0.701 | 1.000 |
|  | Trap location | 1.02 | 8 | 0.402 | 0.407 |
| Colon  F_(15,31)_=1.07, p=0.394, adjusted R^2^=0.021 | *Syphacia* spp. local presence | 0.30 | 1 | 0.924 | 1.000 |
|  | *Syphacia* spp. non-local presence | 0.77 | 1 | 0.513 | 1.000 |
|  | *H. polygyrus* non-local presence | 0.28 | 1 | 0.948 | 1.000 |
|  | *Hymenolepis* sp. (overall) presence | 0.31 | 1 | 0.929 | 1.000 |
|  | *C. vitta* non-local presence | 0.71 | 1 | 0.547 | 1.000 |
|  | Read depth | 1.04 | 1 | 0.348 | 0.696 |
|  | MiSeq run | 0.54 | 1 | 0.703 | 1.000 |
|  | Trap location | 1.41 | 8 | 0.152 | 0.304 |

**Table S4. Relative abundances of bacterial families in faecal samples across the two datasets.** 16S microbiota profiles of faecal samples were obtained in two related studies of wood mice, a dissection study (n=50 mice, 1 sample each) and a longitudinal study (n=221 samples from 105 mice). The total relative abundance of the top 10 bacterial families summed across all faecal samples in each dataset are shown.

| Family | Dissections | Longitudinal |
| --- | --- | --- |
| Lachnospiraceae | 26.70 | 18.26 |
| Muribaculaceae | 29.43 | 37.83 |
| Lactobacillaceae | 25.83 | 23.37 |
| Ruminococcaceae | 8.35 | 7.57 |
| Prevotellaceae | 0.70 | 1.50 |
| Bifidobacteriaceae | 0.16 | 2.47 |
| Rikenellaceae | 2.03 | 1.89 |
| Clostridiales (vadin BB60) | 1.40 | 0.90 |
| Helicobacteraceae | 1.23 | 0.75 |
| Coriobacteriaceae | 1.11 | 1.36 |

**Table S5. Associations between parasite diversity and faecal microbial diversity in the dissection study.** Results are from a Bayesian linear model assessing the effect of the total number of parasite species (parasite richness) detected in the dissection study on faecal microbial richness of each wood mouse (n=42). Trapping location, MiSeq run and read depth (z-transformed) were included as covariates. Posterior means and 95% credible intervals are shown.

| Model term | Posterior mean | Lower CI | Upper CI |
| --- | --- | --- | --- |
| Parasite richness | 0.85 | -18.42 | 20.33 |
| MiSeq run | 7.11 | -41.64 | 55.04 |
| Read depth | **43.64** | **18.18** | **69.14** |
| Trap location (2) | 12.76 | -45.57 | 73.19 |
| Trap location (3) | -0.62 | -60.33 | 58.81 |
| Trap location (3 pines) | 3.07 | -51.21 | 56.48 |
| Trap location (4) | 2.83 | -84.02 | 90.01 |
| Trap location (SW1) | **124.44** | **8.64** | **243.01** |
| Trap location (SW3) | 26.22 | -93.11 | 147.63 |
| Trap location (SW4) | 8.34 | -66.5 | 84.61 |

**Table S6. Associations between individual parasite infections and faecal microbiota composition (Bray-Curtis dissimilarity)**. Results are from a partial redundancy analysis (pRDA) assessing the effect of burden of each parasite detected (z-score of EPG) on faecal microbiome composition in a longitudinal study (n=214). Host ID was including as a conditional term to control for repeated measures. P-values <0.1 are marked with ., <0.05 with * and <0.01 with **.

| Model term | F | df | p-value |
| --- | --- | --- | --- |
| *Aoncotheca* sp. | 0.81 | 1 | 0.629 |
| *T. muris* | 0.987 | 1 | 0.478 |
| *H. polygyrus* | 1.19 | 1 | 0.296 |
| *Syphacia* spp. | 0.91 | 1 | 0.568 |
| *Eimeria* spp. | 1.73 | 1 | 0.065 . |
| *Hymenolepis* sp. | 0.73 | 1 | 0.757 |
| Read depth | 1.31 | 1 | 0.111 |
| MiSeq run | 0.94 | 1 | 0.523 |

**Table S7. Associations between parasite infections and the first axis of microbiota compositional variation (PC1), which shows strong seasonal variation**. Results are from a GAMM assessing the effect of burden of each parasite detected on faecal microbiota PC1 values in a longitudinal study (n=215). A cyclic cubic spline for day of the year was included to model seasonality in PC1. Host ID was including as a random term to control for repeated measures P-values <0.05 are marked with * and p<0.01 with **.

| Model term | F | df | p-value |
| --- | --- | --- | --- |
| *Aoncotheca* sp. | 0.11 | 1 | 0.743 |
| *T. muris* | 4.79 | 1 | 0.039 * |
| *H. polygyrus* | 0.08 | 1 | 0.778 |
| *Syphacia* spp. | 1.35 | 1 | 0.247 |
| *Eimeria* spp. | 7.89 | 1 | 0.005 * |
| *Hymenolepis* sp. | 1.43 | 1 | 0.705 |
| Read depth | 4.34 | 1 | 0.038 * |
| MiSeq run | 1.75 | 1 | 0.676 |
| s(Day of the year) | 8.97 | 5.93(edf) | <0.001 ** |
